# Supplementary material for: The Spread of SARS-CoV-2 Variant Omicron with a Doubling Time of 2.0–3.3 Days Can Be Explained by Immune Evasion
Source: Viruses. 2022 Jan 30;14(2):294. doi: 10.3390/v14020294 (PMC8875689; doi:10.3390/v14020294)
Supplement: Supplementary file 1 [file viruses-14-00294-s001.zip › Table_S3--GISAID-Gauteng-acknowledgments.pdf]

We gratefully acknowledge the following Authors from the Originating laboratories responsible for obtaining the specimens, as well as the Submitting laboratories where the genome data were generated and shared via GISAID, on which this research is based.

All Submitters of data may be contacted directly via www.gisaid.org

Authors are sorted alphabetically.

Accession ID	Originating Laboratory	Submitting Laboratory	Authors
EPI_ISL_2444137, EPI_ISL_2444138, EPI_ISL_2444139, EPI_ISL_2444140, EPI_ISL_2444141, EPI_ISL_2444142, EPI_ISL_2444143, EPI_ISL_2444144, EPI_ISL_2444145, EPI_ISL_2444146, EPI_ISL_2444147, EPI_ISL_2444148, EPI_ISL_2444149, EPI_ISL_2444150, EPI_ISL_2444151, EPI_ISL_2444152			
see above	AAMPATH LABORATORIES	National Institute for Communicable Diseases of the National Health Laboratory Service	Amoako DG; Bhiman JN; Ismail A; Mahlangu B; Mohale T; Ntuli N; Scheepers C
EPI_ISL_7886687, EPI_ISL_7886688, EPI_ISL_7886689, EPI_ISL_7886690	AHRI - Alex Sigal lab	KRISP, KZn Research Innovation and Sequencing Platform	Arisha Maharaj; Bernstein Mallory; Cele Sandile; Giandhari J; Karim Farina; Khan Khadija; Lessells R; Moir M; Naidoo Y; Pillay S; Ramphal U; Ramphal Y; San JE; Sigal Alex; Tegally H; Tshiabuila D; Wilkinson E; de Oliveira T
EPI_ISL_3838578, EPI_ISL_3838579, EPI_ISL_3838580, EPI_ISL_3838581, EPI_ISL_3838583, EPI_ISL_3838584, EPI_ISL_3838586, EPI_ISL_3838594, EPI_ISL_3838595, EPI_ISL_3838596, EPI_ISL_3838597, EPI_ISL_3838598, EPI_ISL_4651621, EPI_ISL_4651622, EPI_ISL_4651629, EPI_ISL_4651637, EPI_ISL_4651641, EPI_ISL_4651646, EPI_ISL_4651652, EPI_ISL_4651660, EPI_ISL_4651664, EPI_ISL_4651665, EPI_ISL_4651673, EPI_ISL_4651678, EPI_ISL_4651692, EPI_ISL_4651700, EPI_ISL_4651701, EPI_ISL_4651703, EPI_ISL_4651714, EPI_ISL_4651719, EPI_ISL_4651720, EPI_ISL_4651727, EPI_ISL_4651741, EPI_ISL_4651742, EPI_ISL_4651748, EPI_ISL_4651751, EPI_ISL_4651771, EPI_ISL_4651782, EPI_ISL_4651786, EPI_ISL_4651795, EPI_ISL_4651796, EPI_ISL_4651805, EPI_ISL_4651809, EPI_ISL_4651816, EPI_ISL_4651823, EPI_ISL_4651831, EPI_ISL_4651832, EPI_ISL_4651843, EPI_ISL_4651849, EPI_ISL_4651854, EPI_ISL_4651875, EPI_ISL_4651888, EPI_ISL_4651914, EPI_ISL_4651930, EPI_ISL_4652093, EPI_ISL_4652103, EPI_ISL_4652108, EPI_ISL_4652117, EPI_ISL_4652127, EPI_ISL_4652135, EPI_ISL_4652141, EPI_ISL_4652145, EPI_ISL_4652146, EPI_ISL_4652153, EPI_ISL_4652158, EPI_ISL_4652159, EPI_ISL_4652169, EPI_ISL_4652170, EPI_ISL_4652175, EPI_ISL_4652180, EPI_ISL_4652181, EPI_ISL_4652185, EPI_ISL_4652186, EPI_ISL_4652191, EPI_ISL_4652194, EPI_ISL_4652195, EPI_ISL_4652201, EPI_ISL_5416273, EPI_ISL_5416289, EPI_ISL_5416298, EPI_ISL_5416299, EPI_ISL_5416344, EPI_ISL_5416371, EPI_ISL_5416508, EPI_ISL_5416509, EPI_ISL_5416510, EPI_ISL_5416511, EPI_ISL_5416512, EPI_ISL_5416513, EPI_ISL_5416514, EPI_ISL_5416515, EPI_ISL_5416516, EPI_ISL_5416517, EPI_ISL_5416518, EPI_ISL_5416519, EPI_ISL_5416520, EPI_ISL_5416522, EPI_ISL_5476869, EPI_ISL_5510394, EPI_ISL_5603071, EPI_ISL_5603072, EPI_ISL_5603073, EPI_ISL_5603075, EPI_ISL_5603076, EPI_ISL_5603077, EPI_ISL_5603078, EPI_ISL_5603079, EPI_ISL_5603080, EPI_ISL_5603081, EPI_ISL_5603082, EPI_ISL_5603083, EPI_ISL_5603084, EPI_ISL_5603120, EPI_ISL_5603121, EPI_ISL_5603130, EPI_ISL_5603139, EPI_ISL_5603156, EPI_ISL_5603159, EPI_ISL_5603161, EPI_ISL_5603166, EPI_ISL_5603172, EPI_ISL_5603183, EPI_ISL_6203619, EPI_ISL_6327724, EPI_ISL_6327725, EPI_ISL_6327726, EPI_ISL_6327727, EPI_ISL_6327728, EPI_ISL_6327729, EPI_ISL_6327730, EPI_ISL_6327731, EPI_ISL_6327836, EPI_ISL_6327850, EPI_ISL_6327862, EPI_ISL_6327865, EPI_ISL_6327880, EPI_ISL_6327881, EPI_ISL_6327882, EPI_ISL_6327896, EPI_ISL_6327897, EPI_ISL_6327902, EPI_ISL_6327913, EPI_ISL_6327917, EPI_ISL_6327920, EPI_ISL_6327931, EPI_ISL_6327938, EPI_ISL_6327962, EPI_ISL_6327970, EPI_ISL_6327977, EPI_ISL_6327987, EPI_ISL_6327996, EPI_ISL_6900030, EPI_ISL_6900031, EPI_ISL_6900033, EPI_ISL_6900037, EPI_ISL_6900042, EPI_ISL_6900044, EPI_ISL_6900050, EPI_ISL_6900051, EPI_ISL_6900052, EPI_ISL_6900055, EPI_ISL_6900058, EPI_ISL_6900059, EPI_ISL_6900061, EPI_ISL_6900062, EPI_ISL_6900063, EPI_ISL_6900064, EPI_ISL_6900065, EPI_ISL_6900072, EPI_ISL_6900076, EPI_ISL_6900078, EPI_ISL_6900079, EPI_ISL_6900080, EPI_ISL_6900081, EPI_ISL_6900082, EPI_ISL_6900083, EPI_ISL_6900084, EPI_ISL_6900087			
see above	AMPATH	National Institute for Communicable Diseases of the National Health Laboratory Service	Amoako DG; Bhiman JN; Everatt J; Ismail A; Mahlangu B; Mnguni A; Mohale T; Ntuli N; Scheepers C
EPI_ISL_2086984, EPI_ISL_2086985, EPI_ISL_2086986, EPI_ISL_2086996, EPI_ISL_2086997, EPI_ISL_2086998, EPI_ISL_2086999, EPI_ISL_2087000, EPI_ISL_2087002, EPI_ISL_2087005, EPI_ISL_2087006, EPI_ISL_2087008, EPI_ISL_2087009, EPI_ISL_2087010, EPI_ISL_2087012, EPI_ISL_2087013, EPI_ISL_2087014, EPI_ISL_2087018, EPI_ISL_2087019, EPI_ISL_2087022, EPI_ISL_2087024			
see above	AMPATH LABORATORIES	National Institute for Communicable Diseases of the National Health Laboratory Service	Amoako DG; Bhiman JN; Ismail A; Mahlangu B; Mohale T; Ntuli N; Scheepers C
EPI_ISL_4029909, EPI_ISL_4029924, EPI_ISL_4029937, EPI_ISL_4029951, EPI_ISL_4029966, EPI_ISL_4029981, EPI_ISL_4029982, EPI_ISL_4029988, EPI_ISL_4029992, EPI_ISL_4029993, EPI_ISL_4029998, EPI_ISL_4030002, EPI_ISL_4030004, EPI_ISL_4030005, EPI_ISL_4030006, EPI_ISL_4030007, EPI_ISL_4030008, EPI_ISL_4030009, EPI_ISL_4030010, EPI_ISL_4030015, EPI_ISL_4030062, EPI_ISL_4030063, EPI_ISL_4030064, EPI_ISL_4030066, EPI_ISL_4030067			
see above	AMPATH LABORATORY	National Institute for Communicable Diseases of the National Health Laboratory Service	Amoako DG; Bhiman JN; Everatt J; Ismail A; Mahlangu B; Mnguni A; Mohale T; Ntuli N; Scheepers C
EPI_ISL_5917889, EPI_ISL_5917982, EPI_ISL_5917984, EPI_ISL_5918032, EPI_ISL_5918041, EPI_ISL_5918047, EPI_ISL_5918050, EPI_ISL_5918074, EPI_ISL_5918090, EPI_ISL_5918092, EPI_ISL_5918101, EPI_ISL_5918107, EPI_ISL_5919986, EPI_ISL_5919994, EPI_ISL_5920002, EPI_ISL_5920016, EPI_ISL_5920102, EPI_ISL_5920133, EPI_ISL_5920137, EPI_ISL_5920155, EPI_ISL_5920159, EPI_ISL_5920164, EPI_ISL_5920170, EPI_ISL_5920196, EPI_ISL_5920204, EPI_ISL_5920214			
see above	Ampath	National Institute for Communicable Diseases of the National Health Laboratory Service	Amoako DG; Bhiman JN; Everatt J; Ismail A; Mahlangu B; Mnguni A; Mohale T; Ntuli N; Scheepers C
EPI_ISL_2178779, EPI_ISL_2178791, EPI_ISL_2178792, EPI_ISL_2193354, EPI_ISL_2193355, EPI_ISL_2193356, EPI_ISL_2193357, EPI_ISL_2193358, EPI_ISL_2193359, EPI_ISL_2193360, EPI_ISL_2193361, EPI_ISL_6914011, EPI_ISL_6914012, EPI_ISL_6914013, EPI_ISL_6914015, EPI_ISL_6914016, EPI_ISL_6914017, EPI_ISL_6914018, EPI_ISL_6914020, EPI_ISL_6914021, EPI_ISL_6914022, EPI_ISL_6914023, EPI_ISL_6914024, EPI_ISL_6914025, EPI_ISL_6914026, EPI_ISL_6914027, EPI_ISL_6914029, EPI_ISL_6914030, EPI_ISL_6914031, EPI_ISL_6914032, EPI_ISL_6914033, EPI_ISL_6914034, EPI_ISL_6914036			
see above	Ampath Laboratories	National Institute for Communicable Diseases of the National Health Laboratory Service	Amoako DG; Bhiman JN; Everatt J; Glass A; Gottberg A; Ismail A; Mahlangu B; Marshall T; Mnguni A; Mohale T; Ntuli N; Scheepers C; Viana R; Wolter N
EPI_ISL_2893300, EPI_ISL_2893301, EPI_ISL_2893302, EPI_ISL_2893303, EPI_ISL_2893304, EPI_ISL_2893305, EPI_ISL_2893306, EPI_ISL_2893307, EPI_ISL_2893308, EPI_ISL_2893309, EPI_ISL_2893310, EPI_ISL_2893311, EPI_ISL_2893312, EPI_ISL_2893313, EPI_ISL_2893314			
see above	BARC - Sisonke	KRISP, KZN Research Innovation and Sequencing Platform	Giandhari Jennifer; Naidoo Yeshnee; Pillay Sureshnee; San James; Sisonke; Tegally Houriiyah; Tshabuila Derek; Wilkinson Eduan; Yajna Ramphal; de Oliveira Tulio
EPI_ISL_2710306, EPI_ISL_2710307, EPI_ISL_2710308, EPI_ISL_2710309, EPI_ISL_2710310, EPI_ISL_2710312, EPI_ISL_2710313, EPI_ISL_2710314, EPI_ISL_2710315, EPI_ISL_2710316, EPI_ISL_2710317, EPI_ISL_2710318, EPI_ISL_2710319, EPI_ISL_2710320, EPI_ISL_2710321			
see above	BARC / Lancet	KRISP, KZN Research Innovation and Sequencing Platform	Giandhari Jennifer; Naidoo Yeshnee; Pillay Sureshnee; San James; Sisonke; Tegally Houriiyah; Tshabuila Derek; Wilkinson Eduan; Yajna Ramphal; de Oliveira Tulio
EPI_ISL_5510285, EPI_ISL_5510294, EPI_ISL_5510298, EPI_ISL_5510351, EPI_ISL_5510367, EPI_ISL_5510370, EPI_ISL_5510378, EPI_ISL_5510383, EPI_ISL_5510396, EPI_ISL_5510397, EPI_ISL_5510401, EPI_ISL_5510407, EPI_ISL_5510410, EPI_ISL_5510411, EPI_ISL_5510445, EPI_ISL_5510447, EPI_ISL_5510448, EPI_ISL_5510452, EPI_ISL_5510453, EPI_ISL_5510462, EPI_ISL_5510471, EPI_ISL_5510490, EPI_ISL_5510491			
see above	Baragwanath	National Institute for Communicable Diseases of the National Health Laboratory Service	Amoako DG; Bhiman JN; Everatt J; Ismail A; Mahlangu B; Mnguni A; Mohale T; Ntuli N; Scheepers C
EPI_ISL_2695506	Bertha gxowa hospital	National Institute for Communicable Diseases of the National Health Laboratory Service	Amoako DG; Bhiman JN; Everatt J; Ismail A; Mahlangu B; Mnguni A; Mohale T; Ntuli N; Scheepers C
EPI_ISL_5510365, EPI_ISL_5510443	Braamfontein Central	National Institute for Communicable Diseases of the National Health Laboratory Service	Amoako DG; Bhiman JN; Everatt J; Ismail A; Mahlangu B; Mnguni A; Mohale T; Ntuli N; Scheepers C
EPI_ISL_2086880, EPI_ISL_2086881, EPI_ISL_2086882, EPI_ISL_2086883, EPI_ISL_2086884, EPI_ISL_2086885, EPI_ISL_2086886, EPI_ISL_2086887, EPI_ISL_2086888, EPI_ISL_2086889, EPI_ISL_2086890, EPI_ISL_2086891, EPI_ISL_2086892, EPI_ISL_2086893, EPI_ISL_2086894, EPI_ISL_2086895, EPI_ISL_2086896, EPI_ISL_2086897, EPI_ISL_2086898, EPI_ISL_2086899, EPI_ISL_2086900, EPI_ISL_2086901, EPI_ISL_2086902, EPI_ISL_2086903, EPI_ISL_2285233, EPI_ISL_2285234, EPI_ISL_2285235, EPI_ISL_2285236, EPI_ISL_2285237, EPI_ISL_2285238, EPI_ISL_2285239, EPI_ISL_2285337, EPI_ISL_2285338			
see above	CHARLOTTE MAXEKE JOHANNESBURG ACADEMIC HOSPITAL	National Institute for Communicable Diseases of the National Health Laboratory Service	Amoako DG; Bhiman JN; Ismail A; Mahlangu B; Mohale T; Ntuli N; Scheepers C
EPI_ISL_2694627, EPI_ISL_2694628, EPI_ISL_2694629, EPI_ISL_2694630, EPI_ISL_2694631, EPI_ISL_2694633, EPI_ISL_2694634, EPI_ISL_2694635, EPI_ISL_2694636, EPI_ISL_2694637, EPI_ISL_2694638, EPI_ISL_2694639, EPI_ISL_2694640, EPI_ISL_2694641, EPI_ISL_2694642, EPI_ISL_2694643, EPI_ISL_2694644, EPI_ISL_2694645, EPI_ISL_2694646, EPI_ISL_2694647, EPI_ISL_2694648, EPI_ISL_2694649, EPI_ISL_2694650, EPI_ISL_2694651, EPI_ISL_2694652, EPI_ISL_2694653, EPI_ISL_2694654, EPI_ISL_2694655, EPI_ISL_2694656, EPI_ISL_2694657, EPI_ISL_2694658, EPI_ISL_2694659, EPI_ISL_2694660, EPI_ISL_2694661, EPI_ISL_2694662, EPI_ISL_2694663, EPI_ISL_2694664, EPI_ISL_2694665, EPI_ISL_2694666, EPI_ISL_2694667, EPI_ISL_2694668, EPI_ISL_2694669, EPI_ISL_2694670, EPI_ISL_2694671, EPI_ISL_2694672, EPI_ISL_2694673, EPI_ISL_2694674, EPI_ISL_2694675, EPI_ISL_2694676, EPI_ISL_2694677, EPI_ISL_2709879, EPI_ISL_2709880, EPI_ISL_2709881, EPI_ISL_2709882, EPI_ISL_2709883, EPI_ISL_2709884, EPI_ISL_2709887, EPI_ISL_2709888, EPI_ISL_2709889, EPI_ISL_2709890, EPI_ISL_2709891, EPI_ISL_2709892, EPI_ISL_2709893, EPI_ISL_2709894, EPI_ISL_2709895, EPI_ISL_2709896, EPI_ISL_2709897, EPI_ISL_2709898, EPI_ISL_2709899, EPI_ISL_2709900, EPI_ISL_2709901, EPI_ISL_2709902, EPI_ISL_2709903, EPI_ISL_2709904, EPI_ISL_2709905, EPI_ISL_2709906, EPI_ISL_2709907, EPI_ISL_2709908, EPI_ISL_2709909, EPI_ISL_2709910, EPI_ISL_2709911, EPI_ISL_2709912, EPI_ISL_2709913, EPI_ISL_2709914, EPI_ISL_2709915, EPI_ISL_2709917, EPI_ISL_2709918, EPI_ISL_2709919, EPI_ISL_2709920, EPI_ISL_2709921, EPI_ISL_2709922, EPI_ISL_2709923, EPI_ISL_2709924, EPI_ISL_2709925, EPI_ISL_2709926, EPI_ISL_2709927, EPI_ISL_2709928, EPI_ISL_2709929, EPI_ISL_2709930, EPI_ISL_2709931, EPI_ISL_2709932, EPI_ISL_2709933, EPI_ISL_2709934, EPI_ISL_2709935, EPI_ISL_2709936, EPI_ISL_2709937, EPI_ISL_2709938, EPI_ISL_2709939, EPI_ISL_2709940, EPI_ISL_2709941, EPI_ISL_2709942, EPI_ISL_5510470			
see above	CHRIS HANI BARAGWANATH	National Institute for Communicable Diseases of the National Health Laboratory Service	Amoako DG; Bhiman JN; Everatt J; Ismail A; Mahlangu B; Mnguni A; Mohale T; Ntuli N; Scheepers C
EPI_ISL_5510372	CHRIS HANI BARAGWANATH HOSPITAL	National Institute for Communicable Diseases of the National Health Laboratory Service	Amoako DG; Bhiman JN; Everatt J; Ismail A; Mahlangu B; Mnguni A; Mohale T; Ntuli N; Scheepers C
EPI_ISL_2086904, EPI_ISL_2086905, EPI_ISL_2086906, EPI_ISL_2086907, EPI_ISL_2086908, EPI_ISL_2086910, EPI_ISL_2086911, EPI_ISL_2086912, EPI_ISL_2086913, EPI_ISL_2086915, EPI_ISL_2086921, EPI_ISL_2086922, EPI_ISL_2086923, EPI_ISL_2086924, EPI_ISL_2087030, EPI_ISL_2087031, EPI_ISL_2087032, EPI_ISL_2087033, EPI_ISL_2087034, EPI_ISL_2087035, EPI_ISL_2087036, EPI_ISL_2087037, EPI_ISL_2087038, EPI_ISL_2087039, EPI_ISL_2087040, EPI_ISL_2087041, EPI_ISL_2087042, EPI_ISL_2087043, EPI_ISL_2087044, EPI_ISL_2087045, EPI_ISL_2087046, EPI_ISL_2087047, EPI_ISL_2153516, EPI_ISL_2153517, EPI_ISL_2153518, EPI_ISL_2153519, EPI_ISL_2153520, EPI_ISL_2153521, EPI_ISL_2153522, EPI_ISL_2153523, EPI_ISL_2153525, EPI_ISL_2153526, EPI_ISL_2153527, EPI_ISL_2153528, EPI_ISL_2153529, EPI_ISL_2153530, EPI_ISL_2153531, EPI_ISL_2153532, EPI_ISL_2153533, EPI_ISL_2153534, EPI_ISL_2153535, EPI_ISL_2153536, EPI_ISL_2153537, EPI_ISL_2273005, EPI_ISL_2273006, EPI_ISL_2273007, EPI_ISL_2273008, EPI_ISL_2273009, EPI_ISL_2273010, EPI_ISL_2273011, EPI_ISL_2273012, EPI_ISL_2273013, EPI_ISL_2273014, EPI_ISL_2273015, EPI_ISL_2273016, EPI_ISL_2273017, EPI_ISL_2273018, EPI_ISL_2273019, EPI_ISL_2273020, EPI_ISL_2273021, EPI_ISL_2273022, EPI_ISL_2273023, EPI_ISL_2273024, EPI_ISL_2273025, EPI_ISL_2273026, EPI_ISL_2273027, EPI_ISL_2273028, EPI_ISL_2273029, EPI_ISL_2273030, EPI_ISL_2273031, EPI_ISL_2273032, EPI_ISL_2273033, EPI_ISL_2273034, EPI_ISL_2273035, EPI_ISL_2273036, EPI_ISL_2273037, EPI_ISL_2273038, EPI_ISL_2273039, EPI_ISL_2273040, EPI_ISL_2273041, EPI_ISL_2273042, EPI_ISL_2273043, EPI_ISL_2273044, EPI_ISL_2273045, EPI_ISL_2273046, EPI_ISL_2273047, EPI_ISL_2273048, EPI_ISL_2273049, EPI_ISL_2273050, EPI_ISL_2273051, EPI_ISL_2273052, EPI_ISL_2273053, EPI_ISL_2273054, EPI_ISL_2273055, EPI_ISL_2273056, EPI_ISL_2285249, EPI_ISL_2285250, EPI_ISL_2382225, EPI_ISL_2382226, EPI_ISL_2382227, EPI_ISL_2382228, EPI_ISL_2382229, EPI_ISL_2382230, EPI_ISL_2382231, EPI_ISL_2382232, EPI_ISL_2382233, EPI_ISL_2382234, EPI_ISL_2382235, EPI_ISL_2382236, EPI_ISL_2382237, EPI_ISL_2382238, EPI_ISL_2382239, EPI_ISL_2382240, EPI_ISL_2382241, EPI_ISL_2382242, EPI_ISL_2382243, EPI_ISL_2382244, EPI_ISL_2382245, EPI_ISL_2382246, EPI_ISL_2382247, EPI_ISL_2382248, EPI_ISL_2382249, EPI_ISL_2382250, EPI_ISL_2382251, EPI_ISL_2382252, EPI_ISL_2382253, EPI_ISL_2382254, EPI_ISL_2382255, EPI_ISL_2382256, EPI_ISL_2382257, EPI_ISL_2382258, EPI_ISL_2382259, EPI_ISL_2382676, EPI_ISL_2382677, EPI_ISL_2382678, EPI_ISL_2382679, EPI_ISL_2382680, EPI_ISL_2382681, EPI_ISL_2382682, EPI_ISL_2382683, EPI_ISL_2382684, EPI_ISL_2382685, EPI_ISL_2382686, EPI_ISL_2382687, EPI_ISL_2382688, EPI_ISL_2382689, EPI_ISL_2382690, EPI_ISL_2382691, EPI_ISL_2382692, EPI_ISL_2382693, EPI_ISL_2382694, EPI_ISL_2382695, EPI_ISL_2382696, EPI_ISL_2382697, EPI_ISL_2382698, EPI_ISL_2442366, EPI_ISL_2442367, EPI_ISL_2442368, EPI_ISL_2442369, EPI_ISL_2454623, EPI_ISL_2454624, EPI_ISL_2454625, EPI_ISL_2454626, EPI_ISL_2454627, EPI_ISL_2454628, EPI_ISL_2454629, EPI_ISL_2454630, EPI_ISL_2454631, EPI_ISL_2454632, EPI_ISL_2454633, EPI_ISL_2454634, EPI_ISL_2454635, EPI_ISL_2454636, EPI_ISL_2454637, EPI_ISL_2454638, EPI_ISL_2454639, EPI_ISL_2454640, EPI_ISL_2454641, EPI_ISL_2454642, EPI_ISL_2454643, EPI_ISL_2454644, EPI_ISL_2454645, EPI_ISL_2454646, EPI_ISL_2454647, EPI_ISL_2454648, EPI_ISL_2454649, EPI_ISL_2454650, EPI_ISL_2454651, EPI_ISL_2454652, EPI_ISL_2454653, EPI_ISL_2454654, EPI_ISL_2454655, EPI_ISL_2454656, EPI_ISL_2454657, EPI_ISL_2454658, EPI_ISL_2454659, EPI_ISL_2582812, EPI_ISL_2582815, EPI_ISL_2582818, EPI_ISL_2582822, EPI_ISL_2582825, EPI_ISL_2662468, EPI_ISL_2662469, EPI_ISL_2662470, EPI_ISL_2662471, EPI_ISL_2662472, EPI_ISL_2662473, EPI_ISL_2662474, EPI_ISL_2662492, EPI_ISL_2662493, EPI_ISL_2662494, EPI_ISL_2662495, EPI_ISL_2662496, EPI_ISL_2662497, EPI_ISL_2662498, EPI_ISL_2662499, EPI_ISL_2662500, EPI_ISL_2662501, EPI_ISL_2662502, EPI_ISL_2662503, EPI_ISL_2662504, EPI_ISL_2662505, EPI_ISL_2662506, EPI_ISL_2662507, EPI_ISL_2662508, EPI_ISL_2662509, EPI_ISL_2662510, EPI_ISL_2662554, EPI_ISL_2662555, EPI_ISL_2662618, EPI_ISL_2662619, EPI_ISL_2662620, EPI_ISL_2662621, EPI_ISL_2662622, EPI_ISL_2662623, EPI_ISL_2662624, EPI_ISL_2662625, EPI_ISL_2662626, EPI_ISL_2662627, EPI_ISL_2662628, EPI_ISL_2662629, EPI_ISL_2662630, EPI_ISL_2662631, EPI_ISL_2662632, EPI_ISL_2662633, EPI_ISL_2662634, EPI_ISL_2662635, EPI_ISL_2662636, EPI_ISL_2662637, EPI_ISL_2662638, EPI_ISL_2662639, EPI_ISL_2662640, EPI_ISL_2662641, EPI_ISL_2662642, EPI_ISL_2662643, EPI_ISL_2662644, EPI_ISL_2662645, EPI_ISL_2662646, EPI_ISL_2662647, EPI_ISL_2662648, EPI_ISL_2662649, EPI_ISL_2662650, EPI_ISL_2662651, EPI_ISL_2662652, EPI_ISL_2662653, EPI_ISL_2662654, EPI_ISL_2662655, EPI_ISL_2662656, EPI_ISL_2662657, EPI_ISL_2662658, EPI_ISL_2662659, EPI_ISL_2662660, EPI_ISL_2662661, EPI_ISL_2827112, EPI_ISL_2827156, EPI_ISL_2827157, EPI_ISL_3024646, EPI_ISL_3024647, EPI_ISL_3024648, EPI_ISL_3024649, EPI_ISL_3024650, EPI_ISL_3024651, EPI_ISL_3024652, EPI_ISL_3024653, EPI_ISL_3024654, EPI_ISL_3024655, EPI_ISL_3024656, EPI_ISL_3024657, EPI_ISL_3024658, EPI_ISL_3024659, EPI_ISL_3024660, EPI_ISL_3024661, EPI_ISL_3024662, EPI_ISL_3024663, EPI_ISL_3024664, EPI_ISL_3024665, EPI_ISL_3024666, EPI_ISL_3024667, EPI_ISL_3024668, EPI_ISL_3024669, EPI_ISL_3024670, EPI_ISL_3024671, EPI_ISL_3024672, EPI_ISL_3024673, EPI_ISL_3024674, EPI_ISL_3024675, EPI_ISL_3024676, EPI_ISL_3024677, EPI_ISL_3024678, EPI_ISL_3024679, EPI_ISL_3024680, EPI_ISL_3024681, EPI_ISL_3030439, EPI_ISL_3030440, EPI_ISL_3451304, EPI_ISL_3451305, EPI_ISL_3451306, EPI_ISL_3451307, EPI_ISL_3451308, EPI_ISL_3451309, EPI_ISL_3451310, EPI_ISL_3451311, EPI_ISL_3451312, EPI_ISL_3451313, EPI_ISL_3451314, EPI_ISL_3451315, EPI_ISL_3451316, EPI_ISL_3451317, EPI_ISL_3451318, EPI_ISL_3451319, EPI_ISL_3451320, EPI_ISL_3451321, EPI_ISL_3451322, EPI_ISL_3451323, EPI_ISL_3451324, EPI_ISL_3451325, EPI_ISL_3451326, EPI_ISL_3451327, EPI_ISL_3451328, EPI_ISL_3451329, EPI_ISL_3451439, EPI_ISL_3451440, EPI_ISL_3451441, EPI_ISL_3451442, EPI_ISL_3838343, EPI_ISL_3838344, EPI_ISL_3838345, EPI_ISL_3838346, EPI_ISL_3838347, EPI_ISL_3838348, EPI_ISL_3838349, EPI_ISL_3838350, EPI_ISL_3838351, EPI_ISL_3838352, EPI_ISL_3838353, EPI_ISL_3838354, EPI_ISL_3838355, EPI_ISL_3838356, EPI_ISL_3838503, EPI_ISL_3838504, EPI_ISL_3838505, EPI_ISL_3838506, EPI_ISL_3838507, EPI_ISL_3838508, EPI_ISL_3838509, EPI_ISL_3838559, EPI_ISL_3838560, EPI_ISL_3838561, EPI_ISL_3838562, EPI_ISL_3838563, EPI_ISL_3838564, EPI_ISL_3838565, EPI_ISL_5416339, EPI_ISL_5416353, EPI_ISL_5416354, EPI_ISL_5416362, EPI_ISL_5416368, EPI_ISL_5416475, EPI_ISL_6201546, EPI_ISL_6201554, EPI_ISL_6201569, EPI_ISL_6201919, EPI_ISL_6202099, EPI_ISL_6202172, EPI_ISL_6202591, EPI_ISL_6327743, EPI_ISL_6327744, EPI_ISL_6327838, EPI_ISL_6327839, EPI_ISL_6327847, EPI_ISL_6327869, EPI_ISL_6327879, EPI_ISL_6327925, EPI_ISL_6327947, EPI_ISL_6327973, EPI_ISL_6693527, EPI_ISL_6693528, EPI_ISL_6693529, EPI_ISL_6693530, EPI_ISL_6693531, EPI_ISL_6693532, EPI_ISL_6693533, EPI_ISL_6693534, EPI_ISL_6693535, EPI_ISL_6693536, EPI_ISL_6693537, EPI_ISL_6693538, EPI_ISL_6693539, EPI_ISL_6693540, EPI_ISL_6693541, EPI_ISL_6693542, EPI_ISL_6693546, EPI_ISL_6693563, EPI_ISL_6693565, EPI_ISL_6693571, EPI_ISL_6693575, EPI_ISL_6693581, EPI_ISL_6693588, EPI_ISL_6693597, EPI_ISL_6693608, EPI_ISL_6693614, EPI_ISL_6693617, EPI_ISL_6693620, EPI_ISL_6693628, EPI_ISL_6693630, EPI_ISL_6900016, EPI_ISL_6900017, EPI_ISL_6900019, EPI_ISL_6900020, EPI_ISL_6906406, EPI_ISL_6906448, EPI_ISL_6906474, EPI_ISL_6906491, EPI_ISL_7971610, EPI_ISL_7971611, EPI_ISL_7971613, EPI_ISL_7971614, EPI_ISL_7971615, EPI_ISL_7971646, EPI_ISL_7971647, EPI_ISL_7971648, EPI_ISL_7971649, EPI_ISL_7971650, EPI_ISL_7971651, EPI_ISL_7971652, EPI_ISL_7971653, EPI_ISL_7971654, EPI_ISL_7971674, EPI_ISL_7971675, EPI_ISL_7971676, EPI_ISL_7971677, EPI_ISL_7971678, EPI_ISL_7971679, EPI_ISL_7971680, EPI_ISL_7971681, EPI_ISL_7971682, EPI_ISL_7971683, EPI_ISL_7971684, EPI_ISL_7971685, EPI_ISL_7971686, EPI_ISL_7971687, EPI_ISL_7971688, EPI_ISL_7971689, EPI_ISL_7971690, EPI_ISL_7971691, EPI_ISL_7971692, EPI_ISL_8182816, EPI_ISL_8182817, EPI_ISL_8182818, EPI_ISL_8182819, EPI_ISL_8182820, EPI_ISL_8182821, EPI_ISL_8182822, EPI_ISL_8182823, EPI_ISL_8182824, EPI_ISL_8182825, EPI_ISL_8182826, EPI_ISL_8182827, EPI_ISL_8182828, EPI_ISL_8182829, EPI_ISL_8182857, EPI_ISL_8182858, EPI_ISL_8182859, EPI_ISL_8182860, EPI_ISL_8182861, EPI_ISL_8182862, EPI_ISL_8182863, EPI_ISL_8182885, EPI_ISL_8182886, EPI_ISL_8182887, EPI_ISL_8182888, EPI_ISL_8182889, EPI_ISL_8182890			
see above	CHRIS HANI BARAGWANATH LABORATORY	National Institute for Communicable Diseases of the National Health Laboratory Service	Amoako DG; Bhiman JN; Everatt J; Ismail A; Kekana D; Mahlangu B; Mnguni A; Mohale T; Ntuli N; Scheepers C; Wolter N

EPI_ISL_2463311	Charlotte Maxeke	National Institute for Communicable Diseases of the National Health Laboratory Service	Amoako DG; Bhiman JN; Everatt J; Glass A; Sisonke Team; Viana R
EPI_ISL_7337463, EPI_ISL_7337464, EPI_ISL_7337465, EPI_ISL_7337466, EPI_ISL_7337467, EPI_ISL_7337468, EPI_ISL_7337469, EPI_ISL_7337470, EPI_ISL_7337471, EPI_ISL_7337473, EPI_ISL_7337474, EPI_ISL_7337475, EPI_ISL_7337476, EPI_ISL_7337477, EPI_ISL_7337478, EPI_ISL_7337479, EPI_ISL_7337480, EPI_ISL_7337481, EPI_ISL_7337482, EPI_ISL_7337483, EPI_ISL_7337484, EPI_ISL_7337485, EPI_ISL_7337486, EPI_ISL_7337487, EPI_ISL_7337488, EPI_ISL_7337489, EPI_ISL_7337490, EPI_ISL_7337495, EPI_ISL_7337496, EPI_ISL_7337497, EPI_ISL_7337498, EPI_ISL_7337499, EPI_ISL_7337500, EPI_ISL_7337501, EPI_ISL_7337502, EPI_ISL_7337503, EPI_ISL_7337504, EPI_ISL_7337505, EPI_ISL_7337506, EPI_ISL_7337507, EPI_ISL_7337508, EPI_ISL_7337509, EPI_ISL_7337510, EPI_ISL_7337511			
see above	Charlotte Maxeke Johannesburg Academic Hospital	National Institute for Communicable Diseases of the National Health Laboratory Service	Amoako DG; Bhiman JN; Everatt J; Ismail A; Mahlangu B; Mnguni A; Mohale T; Ntuli N; Scheepers C
EPI_ISL_940075, EPI_ISL_940076, EPI_ISL_940077, EPI_ISL_940078, EPI_ISL_940079, EPI_ISL_940080, EPI_ISL_940081, EPI_ISL_940082, EPI_ISL_940083, EPI_ISL_940084, EPI_ISL_940085, EPI_ISL_940086, EPI_ISL_940087, EPI_ISL_940088, EPI_ISL_940089, EPI_ISL_940090, EPI_ISL_940091, EPI_ISL_940092, EPI_ISL_940093, EPI_ISL_940094, EPI_ISL_940095, EPI_ISL_940096, EPI_ISL_940097, EPI_ISL_940098, EPI_ISL_940099, EPI_ISL_940100, EPI_ISL_940101, EPI_ISL_940102, EPI_ISL_940103, EPI_ISL_940104, EPI_ISL_940106, EPI_ISL_940107, EPI_ISL_940109, EPI_ISL_940110, EPI_ISL_940111, EPI_ISL_940112, EPI_ISL_940113, EPI_ISL_940114, EPI_ISL_940115, EPI_ISL_940116, EPI_ISL_940117, EPI_ISL_940118, EPI_ISL_940119, EPI_ISL_940120, EPI_ISL_940121, EPI_ISL_940122, EPI_ISL_940123, EPI_ISL_940124, EPI_ISL_940126, EPI_ISL_940127, EPI_ISL_940128, EPI_ISL_940129, EPI_ISL_940130, EPI_ISL_940131, EPI_ISL_940132, EPI_ISL_940133, EPI_ISL_940134, EPI_ISL_940135, EPI_ISL_940136, EPI_ISL_940137, EPI_ISL_1239267, EPI_ISL_1239268, EPI_ISL_1239269, EPI_ISL_1239270, EPI_ISL_1239271, EPI_ISL_1239272, EPI_ISL_1239273, EPI_ISL_1239274, EPI_ISL_1239275, EPI_ISL_1239276, EPI_ISL_1239277, EPI_ISL_1239278, EPI_ISL_1239279, EPI_ISL_1239280, EPI_ISL_1239281, EPI_ISL_1239282, EPI_ISL_1239283, EPI_ISL_1239284, EPI_ISL_1239285, EPI_ISL_1239287, EPI_ISL_1239288, EPI_ISL_1239289, EPI_ISL_1239290, EPI_ISL_1239291, EPI_ISL_1239292, EPI_ISL_1239293, EPI_ISL_1239294, EPI_ISL_1239295, EPI_ISL_1239296, EPI_ISL_1239297, EPI_ISL_1239298, EPI_ISL_1239299, EPI_ISL_1239300, EPI_ISL_1239301, EPI_ISL_1239302, EPI_ISL_1239303, EPI_ISL_1239304, EPI_ISL_1239305, EPI_ISL_1239306, EPI_ISL_1239307, EPI_ISL_1239308, EPI_ISL_1239309, EPI_ISL_1239310, EPI_ISL_1239311, EPI_ISL_1239312, EPI_ISL_1239313, EPI_ISL_1239314, EPI_ISL_1239315, EPI_ISL_1239316, EPI_ISL_1239317, EPI_ISL_1239318, EPI_ISL_1239319, EPI_ISL_1239320, EPI_ISL_1239321, EPI_ISL_1239322, EPI_ISL_1239323, EPI_ISL_1239324, EPI_ISL_1239325, EPI_ISL_1239326, EPI_ISL_1239328, EPI_ISL_1239329			
see above	Charlotte Maxeke Johannesburg Academic Hospital, National Health Laboratory Services, Gauteng, South Africa	National Institute for Communicable Diseases of the National Health Laboratory Service	Allam M; Amoako DG; Bhiman JN; Ismail A; Mahlangu B; Mohale T; Ntuli N; Scheepers C
EPI_ISL_4474433, EPI_ISL_4474434, EPI_ISL_4474435, EPI_ISL_4474436, EPI_ISL_4474437, EPI_ISL_4474438, EPI_ISL_4474439, EPI_ISL_4474440, EPI_ISL_4474441, EPI_ISL_4474442, EPI_ISL_4474443			
see above	Charlotte Maxeke Johannesburg hospital	CERI, Centre for Epidemic Response and Innovation	Emmanuel S; Florette Treurnicht; Giandhari J; Kathleen Subramoney; Naidoo Yeshnee; Pillay S; Tegally H; Tshabuila Derek; Wilkinson E; Yajna Ramphal; de Oliveira T
EPI_ISL_3118689, EPI_ISL_3118690, EPI_ISL_3118691, EPI_ISL_3118692, EPI_ISL_3118693, EPI_ISL_3118694, EPI_ISL_3118695, EPI_ISL_3118696			
see above	Chris Hani Baragwanath Academic Hospital POC laboratory	KRISP, KZn Research Innovation and Sequencing Platform	Brown MJ; Emmanuel SJ; Giandhari J; Lessells R; Omar S; Pillay S; Tegally H; Wadula J; Wilkinson E; Yajna R; de Oliveira T
EPI_ISL_1239330, EPI_ISL_1239331, EPI_ISL_1239332, EPI_ISL_1239333, EPI_ISL_1239334, EPI_ISL_1239335, EPI_ISL_1239336, EPI_ISL_1239337, EPI_ISL_1239338, EPI_ISL_1239339, EPI_ISL_1239340, EPI_ISL_1239341, EPI_ISL_1239342, EPI_ISL_1239343, EPI_ISL_1239344, EPI_ISL_1239345, EPI_ISL_1239346, EPI_ISL_1239347, EPI_ISL_1239348, EPI_ISL_1239349, EPI_ISL_1239350, EPI_ISL_1239351, EPI_ISL_1239352, EPI_ISL_1239353, EPI_ISL_1239354, EPI_ISL_1239355, EPI_ISL_1239356, EPI_ISL_1239357, EPI_ISL_1239358, EPI_ISL_1239360			
see above	Chris Hani Baragwanath Hospital, National Health Laboratory Services, Gauteng, South Africa	National Institute for Communicable Diseases of the National Health Laboratory Service	Amoako DG; Bhiman JN; Ismail A; Mahlangu B; Mohale T; Ntuli N; Scheepers C
EPI_ISL_5917986, EPI_ISL_5917992, EPI_ISL_5918009, EPI_ISL_7337512, EPI_ISL_7337513, EPI_ISL_7337514, EPI_ISL_7337515, EPI_ISL_7337516, EPI_ISL_7337517, EPI_ISL_7337518, EPI_ISL_7337519, EPI_ISL_7337520, EPI_ISL_7337521, EPI_ISL_7337522, EPI_ISL_7337523, EPI_ISL_7337524, EPI_ISL_7337525, EPI_ISL_7337527, EPI_ISL_7337528, EPI_ISL_7337529, EPI_ISL_7337530, EPI_ISL_7337531, EPI_ISL_7337532, EPI_ISL_7337533, EPI_ISL_7337534, EPI_ISL_7337536, EPI_ISL_7337537, EPI_ISL_7337538, EPI_ISL_7337539, EPI_ISL_7337540, EPI_ISL_7337541, EPI_ISL_7337542, EPI_ISL_7337543, EPI_ISL_7337544, EPI_ISL_7337545, EPI_ISL_7337546, EPI_ISL_7337547, EPI_ISL_7337548, EPI_ISL_7337549, EPI_ISL_7337550, EPI_ISL_7337551, EPI_ISL_7337553, EPI_ISL_7337558, EPI_ISL_7337559, EPI_ISL_7337560, EPI_ISL_7337561, EPI_ISL_7337562, EPI_ISL_7337563, EPI_ISL_7337564, EPI_ISL_7456412			
see above	Chris Hani Baragwanath Laboratory	National Institute for Communicable Diseases of the National Health Laboratory Service	Amoako DG; Bhiman JN; Everatt J; Ismail A; Mahlangu B; Mnguni A; Mohale T; Ntuli N; Scheepers C; Wolter N
EPI_ISL_3342336, EPI_ISL_3342338, EPI_ISL_3342339, EPI_ISL_3342340, EPI_ISL_3342342, EPI_ISL_3342344, EPI_ISL_3342346, EPI_ISL_3342347, EPI_ISL_3342348, EPI_ISL_3411472			
see above	DGM Laboratory	National Institute for Communicable Diseases of the National Health Laboratory Service	Amoako DG; Bhiman JN; Everatt J; Ismail A; Mahlangu B; Mnguni A; Mohale T; Ntuli N; Scheepers C
EPI_ISL_2153483, EPI_ISL_2153485, EPI_ISL_2153486, EPI_ISL_2153487, EPI_ISL_2153488, EPI_ISL_2153489, EPI_ISL_2153490, EPI_ISL_2153491, EPI_ISL_2153493, EPI_ISL_2153494, EPI_ISL_2153495, EPI_ISL_2153496, EPI_ISL_2153497, EPI_ISL_2153498, EPI_ISL_2153499, EPI_ISL_2153500, EPI_ISL_2153501, EPI_ISL_2153502, EPI_ISL_2153503, EPI_ISL_2153504, EPI_ISL_2153505, EPI_ISL_2153506, EPI_ISL_2153507, EPI_ISL_2153508, EPI_ISL_2153509, EPI_ISL_2153510, EPI_ISL_2153511, EPI_ISL_2153512, EPI_ISL_2153513, EPI_ISL_2153514, EPI_ISL_2153515, EPI_ISL_2285251, EPI_ISL_2285252, EPI_ISL_2582809, EPI_ISL_2662511, EPI_ISL_2662512, EPI_ISL_2662513, EPI_ISL_2662514, EPI_ISL_2662515, EPI_ISL_2662516, EPI_ISL_2662517, EPI_ISL_2662518, EPI_ISL_2662519, EPI_ISL_2662520, EPI_ISL_2662521, EPI_ISL_2662522, EPI_ISL_2662523, EPI_ISL_2662524, EPI_ISL_2662525, EPI_ISL_2662526, EPI_ISL_2662527, EPI_ISL_2662528, EPI_ISL_2662538, EPI_ISL_2662539, EPI_ISL_2662540, EPI_ISL_2662541, EPI_ISL_2662542, EPI_ISL_2662543, EPI_ISL_2662544, EPI_ISL_2662545, EPI_ISL_2662546, EPI_ISL_2662547, EPI_ISL_2662548, EPI_ISL_2662549, EPI_ISL_2662551, EPI_ISL_2662552			
see above	DR GEORGE MUKHARI LABORATORY	National Institute for Communicable Diseases of the National Health Laboratory Service	Amoako DG; Bhiman JN; Everatt J; Ismail A; Mahlangu B; Mnguni A; Mohale T; Ntuli N; Scheepers C
EPI_ISL_2382663	Dr George Mukhari Academic Hospital	National Institute for Communicable Diseases of the National Health Laboratory Service	Amoako DG; Bhiman JN; Ismail A; Mahlangu B; Mohale T; Ntuli N; Scheepers C
EPI_ISL_2695588, EPI_ISL_2695589, EPI_ISL_2695590, EPI_ISL_2695591, EPI_ISL_2695592, EPI_ISL_2695593, EPI_ISL_2695594, EPI_ISL_2695595, EPI_ISL_2695596, EPI_ISL_2695597, EPI_ISL_2695598, EPI_ISL_2695599, EPI_ISL_2695600, EPI_ISL_2695601, EPI_ISL_2695602, EPI_ISL_2695603, EPI_ISL_2695604, EPI_ISL_2695605, EPI_ISL_2695606, EPI_ISL_2695727, EPI_ISL_2695728, EPI_ISL_2695729, EPI_ISL_2695730, EPI_ISL_2695731, EPI_ISL_2695732, EPI_ISL_2695733, EPI_ISL_2695734, EPI_ISL_2695735, EPI_ISL_2695736, EPI_ISL_2695737, EPI_ISL_2695738, EPI_ISL_2695739, EPI_ISL_2695740, EPI_ISL_2695741, EPI_ISL_2695742, EPI_ISL_2695743, EPI_ISL_2695744, EPI_ISL_2695745, EPI_ISL_2695746, EPI_ISL_2695747, EPI_ISL_2695748, EPI_ISL_2695749, EPI_ISL_2695750, EPI_ISL_2695751, EPI_ISL_2709863, EPI_ISL_2709864, EPI_ISL_2709872, EPI_ISL_2709873, EPI_ISL_2709874, EPI_ISL_2709875, EPI_ISL_2709876, EPI_ISL_2709877, EPI_ISL_2709878, EPI_ISL_2709885, EPI_ISL_2709886			
see above	HELEN JOSEPH	National Institute for Communicable Diseases of the National Health Laboratory Service	Amoako DG; Bhiman JN; Everatt J; Ismail A; Mahlangu B; Mnguni A; Mohale T; Ntuli N; Scheepers C
EPI_ISL_2827072, EPI_ISL_2827073, EPI_ISL_2827074, EPI_ISL_2827075, EPI_ISL_2827076, EPI_ISL_2827077, EPI_ISL_2827078, EPI_ISL_2827079, EPI_ISL_2827080, EPI_ISL_2827081, EPI_ISL_2827082, EPI_ISL_2827083, EPI_ISL_2827084, EPI_ISL_2827085, EPI_ISL_2827086, EPI_ISL_2827087, EPI_ISL_2827088, EPI_ISL_2827089, EPI_ISL_2827090, EPI_ISL_2827091, EPI_ISL_2827092, EPI_ISL_2827093, EPI_ISL_2827094, EPI_ISL_2827095, EPI_ISL_2827096, EPI_ISL_2827097, EPI_ISL_2827098, EPI_ISL_2827099, EPI_ISL_2827100, EPI_ISL_2827101, EPI_ISL_2827102, EPI_ISL_2827103, EPI_ISL_2827104, EPI_ISL_2827105, EPI_ISL_2827106, EPI_ISL_2827107, EPI_ISL_2827108, EPI_ISL_2827109, EPI_ISL_2827110, EPI_ISL_2827111, EPI_ISL_2827113, EPI_ISL_2827114, EPI_ISL_2827115, EPI_ISL_2827116, EPI_ISL_2827117, EPI_ISL_2827118, EPI_ISL_2827119, EPI_ISL_2827120, EPI_ISL_2827121, EPI_ISL_2827122, EPI_ISL_2827123, EPI_ISL_2827124, EPI_ISL_2827125, EPI_ISL_2827126, EPI_ISL_2827127, EPI_ISL_2827128, EPI_ISL_2827129, EPI_ISL_2827130, EPI_ISL_2827131, EPI_ISL_2827132, EPI_ISL_2895772, EPI_ISL_2895773, EPI_ISL_2895774, EPI_ISL_2895775, EPI_ISL_2895776, EPI_ISL_2895777, EPI_ISL_2895778, EPI_ISL_2895779, EPI_ISL_2895780, EPI_ISL_2895781, EPI_ISL_2895782, EPI_ISL_2895783, EPI_ISL_2895784, EPI_ISL_2895785, EPI_ISL_2895786, EPI_ISL_2895787, EPI_ISL_2895788, EPI_ISL_2895789, EPI_ISL_2895790, EPI_ISL_2895791, EPI_ISL_2895792, EPI_ISL_2895793, EPI_ISL_2895794, EPI_ISL_2895795, EPI_ISL_2895796, EPI_ISL_2895797, EPI_ISL_2942284, EPI_ISL_2942285, EPI_ISL_2942286, EPI_ISL_2942287, EPI_ISL_2964647, EPI_ISL_2984812, EPI_ISL_2984813, EPI_ISL_2984814, EPI_ISL_2984815, EPI_ISL_2984816, EPI_ISL_2984817, EPI_ISL_2984818, EPI_ISL_2984819, EPI_ISL_2984820, EPI_ISL_2984821, EPI_ISL_2984822, EPI_ISL_2984823, EPI_ISL_2984824, EPI_ISL_2984825, EPI_ISL_2984826, EPI_ISL_2984827, EPI_ISL_2984828, EPI_ISL_2984829, EPI_ISL_2984830, EPI_ISL_2984831, EPI_ISL_2984832, EPI_ISL_2984833, EPI_ISL_2984834, EPI_ISL_2984835, EPI_ISL_2984893, EPI_ISL_2984894, EPI_ISL_2984895, EPI_ISL_2984896, EPI_ISL_2984897, EPI_ISL_2984898, EPI_ISL_2984899, EPI_ISL_2984900, EPI_ISL_2984901, EPI_ISL_2984902, EPI_ISL_2984903, EPI_ISL_2984904, EPI_ISL_2984905, EPI_ISL_2984906, EPI_ISL_2984907, EPI_ISL_2984908, EPI_ISL_2984909, EPI_ISL_2984910, EPI_ISL_2987234, EPI_ISL_2988404, EPI_ISL_2988405, EPI_ISL_2988408, EPI_ISL_2988409, EPI_ISL_2988878, EPI_ISL_3024575, EPI_ISL_3024576, EPI_ISL_3024577, EPI_ISL_3024578, EPI_ISL_3024579, EPI_ISL_3024580, EPI_ISL_3024581, EPI_ISL_3024582, EPI_ISL_3024583, EPI_ISL_3024584, EPI_ISL_3024585, EPI_ISL_3024586, EPI_ISL_3024587, EPI_ISL_3024588, EPI_ISL_3024589, EPI_ISL_3024590, EPI_ISL_3024591, EPI_ISL_3024592, EPI_ISL_3024593, EPI_ISL_3024594, EPI_ISL_3024595, EPI_ISL_3024596, EPI_ISL_3024597, EPI_ISL_3024598, EPI_ISL_3024599, EPI_ISL_3024600, EPI_ISL_3024601, EPI_ISL_3024602, EPI_ISL_3024603, EPI_ISL_3024604, EPI_ISL_3024605, EPI_ISL_3024606, EPI_ISL_3024607, EPI_ISL_3024608, EPI_ISL_3024609, EPI_ISL_3024610, EPI_ISL_3024611, EPI_ISL_3024612, EPI_ISL_3024613, EPI_ISL_3024614, EPI_ISL_3024615, EPI_ISL_3024616, EPI_ISL_3024617, EPI_ISL_3024618, EPI_ISL_3024619, EPI_ISL_3024620, EPI_ISL_3024621, EPI_ISL_3024622, EPI_ISL_3024623, EPI_ISL_3024624, EPI_ISL_3024625, EPI_ISL_3024626, EPI_ISL_3030436, EPI_ISL_3030437, EPI_ISL_3030438, EPI_ISL_3032185, EPI_ISL_3451530, EPI_ISL_3451531, EPI_ISL_3838401, EPI_ISL_3838402, EPI_ISL_3838403, EPI_ISL_3838404, EPI_ISL_3838405, EPI_ISL_3838406, EPI_ISL_3838407, EPI_ISL_3838408, EPI_ISL_3838409, EPI_ISL_3838410, EPI_ISL_3838411, EPI_ISL_3838412, EPI_ISL_3838413, EPI_ISL_3838414, EPI_ISL_3838415, EPI_ISL_3838416, EPI_ISL_3838417, EPI_ISL_3838418, EPI_ISL_3838419, EPI_ISL_3838420, EPI_ISL_3838421, EPI_ISL_3838422, EPI_ISL_3838423, EPI_ISL_3838424, EPI_ISL_3838425, EPI_ISL_3838426, EPI_ISL_4253615, EPI_ISL_4253616, EPI_ISL_4253617, EPI_ISL_4253618, EPI_ISL_4253619, EPI_ISL_4253620, EPI_ISL_4253621, EPI_ISL_4253622, EPI_ISL_4253623, EPI_ISL_4253624, EPI_ISL_4253625, EPI_ISL_4253626, EPI_ISL_4253627, EPI_ISL_4253628, EPI_ISL_4253629, EPI_ISL_4253630, EPI_ISL_4253631, EPI_ISL_4253632, EPI_ISL_4253633, EPI_ISL_4253634, EPI_ISL_4253635, EPI_ISL_4253636, EPI_ISL_4253637, EPI_ISL_4253638, EPI_ISL_4253639, EPI_ISL_4253640, EPI_ISL_4253641, EPI_ISL_4253642, EPI_ISL_4253643, EPI_ISL_4253644, EPI_ISL_4253645, EPI_ISL_4253646, EPI_ISL_4651672, EPI_ISL_4651733, EPI_ISL_4651850, EPI_ISL_4651941, EPI_ISL_4651945, EPI_ISL_4651959, EPI_ISL_4651962, EPI_ISL_4651970, EPI_ISL_5416275, EPI_ISL_5416276, EPI_ISL_5416282, EPI_ISL_5416288, EPI_ISL_5416292, EPI_ISL_5416312, EPI_ISL_5416321, EPI_ISL_5416323, EPI_ISL_5416324, EPI_ISL_5416355, EPI_ISL_5416383, EPI_ISL_5416465, EPI_ISL_5416466, EPI_ISL_5416467, EPI_ISL_5416468, EPI_ISL_5416469, EPI_ISL_5416470, EPI_ISL_5416471, EPI_ISL_5416472, EPI_ISL_6900028, EPI_ISL_6900095, EPI_ISL_6900139, EPI_ISL_6900140, EPI_ISL_6900141, EPI_ISL_6900142, EPI_ISL_6900143, EPI_ISL_6906407, EPI_ISL_7740761, EPI_ISL_7740762, EPI_ISL_7740765, EPI_ISL_7740768, EPI_ISL_7740769, EPI_ISL_7740770, EPI_ISL_7740771, EPI_ISL_7740772, EPI_ISL_7740773, EPI_ISL_7740774, EPI_ISL_7740775, EPI_ISL_7740776, EPI_ISL_7740777, EPI_ISL_7740778, EPI_ISL_7740779, EPI_ISL_7740780, EPI_ISL_7740781, EPI_ISL_7740782, EPI_ISL_7740783, EPI_ISL_7740784, EPI_ISL_7740785, EPI_ISL_7740786, EPI_ISL_7740787, EPI_ISL_7740788, EPI_ISL_7740789, EPI_ISL_7740790, EPI_ISL_7740791, EPI_ISL_7740792, EPI_ISL_7740793, EPI_ISL_7740794, EPI_ISL_7740795, EPI_ISL_7740796, EPI_ISL_7740797, EPI_ISL_7740801, EPI_ISL_7740802, EPI_ISL_7740803, EPI_ISL_7740804, EPI_ISL_7740805, EPI_ISL_7740806, EPI_ISL_7740807, EPI_ISL_7740808, EPI_ISL_7740809, EPI_ISL_7740810, EPI_ISL_7740811, EPI_ISL_7740817, EPI_ISL_7740818, EPI_ISL_7740819, EPI_ISL_7740820, EPI_ISL_7740821, EPI_ISL_7740822, EPI_ISL_7740823, EPI_ISL_7740824, EPI_ISL_7740825, EPI_ISL_7740826, EPI_ISL_7740827, EPI_ISL_7740828, EPI_ISL_7740829, EPI_ISL_7740830, EPI_ISL_7740831, EPI_ISL_7740832, EPI_ISL_7740833, EPI_ISL_7740834, EPI_ISL_7740835, EPI_ISL_7740836, EPI_ISL_7740837, EPI_ISL_7740838, EPI_ISL_7740839, EPI_ISL_7740840, EPI_ISL_7740841, EPI_ISL_7740842, EPI_ISL_7740843, EPI_ISL_7740844, EPI_ISL_7740845, EPI_ISL_7740846, EPI_ISL_7740847, EPI_ISL_7740848, EPI_ISL_7740849, EPI_ISL_7740850, EPI_ISL_7740851, EPI_ISL_7740852, EPI_ISL_7740853, EPI_ISL_7740862, EPI_ISL_7740863, EPI_ISL_7740864, EPI_ISL_7740867, EPI_ISL_7740868, EPI_ISL_7740870, EPI_ISL_7740871, EPI_ISL_7740872, EPI_ISL_7740873, EPI_ISL_7740874, EPI_ISL_7740876, EPI_ISL_7740877, EPI_ISL_7740879, EPI_ISL_7740880, EPI_ISL_7740881, EPI_ISL_7740882, EPI_ISL_7740884, EPI_ISL_7740886, EPI_ISL_7740888, EPI_ISL_7740891, EPI_ISL_7740892, EPI_ISL_7740894, EPI_ISL_7740895, EPI_ISL_7740896, EPI_ISL_7740898, EPI_ISL_7740899			
see above	HELEN JOSEPH LABORATORY	National Institute for Communicable Diseases of the National Health Laboratory Service	Amoako DG; Bhiman JN; Everatt J; Ismail A; Mahlangu B; Mnguni A; Mohale T; Ntuli N; Scheepers C; Wolter N
EPI_ISL_2271973, EPI_ISL_2271974, EPI_ISL_2271983, EPI_ISL_2271995, EPI_ISL_2271996, EPI_ISL_2271997, EPI_ISL_2271998, EPI_ISL_2272001, EPI_ISL_2272002, EPI_ISL_2272003, EPI_ISL_2272005, EPI_ISL_2272006, EPI_ISL_2272017, EPI_ISL_2272022, EPI_ISL_5510433			
see above	Helen Joseph	National Institute for Communicable Diseases of the National Health Laboratory Service	Amoako DG; Bhiman JN; Everatt J; Ismail A; Mahlangu B; Mnguni A; Mohale T; Ntuli N; Scheepers C
EPI_ISL_3342734, EPI_ISL_3342735, EPI_ISL_4891523, EPI_ISL_4891634, EPI_ISL_4891635, EPI_ISL_4891643, EPI_ISL_4891658, EPI_ISL_4891662, EPI_ISL_4891779, EPI_ISL_4891780, EPI_ISL_4891781, EPI_ISL_4891782, EPI_ISL_4891783, EPI_ISL_5917851, EPI_ISL_5917869, EPI_ISL_5917880, EPI_ISL_5917886, EPI_ISL_5917956, EPI_ISL_5917991, EPI_ISL_5918006, EPI_ISL_5918016, EPI_ISL_5918018, EPI_ISL_5918025, EPI_ISL_5918034, EPI_ISL_5918065, EPI_ISL_5918093, EPI_ISL_5918155, EPI_ISL_5918156, EPI_ISL_5918157, EPI_ISL_5918158, EPI_ISL_7337440, EPI_ISL_7337441, EPI_ISL_7337442, EPI_ISL_7337443, EPI_ISL_7337444, EPI_ISL_7337445, EPI_ISL_7337446, EPI_ISL_7337447, EPI_ISL_7337448, EPI_ISL_7337449, EPI_ISL_7337450, EPI_ISL_7337451, EPI_ISL_7337452, EPI_ISL_7337453, EPI_ISL_7337454, EPI_ISL_7337455, EPI_ISL_7337456, EPI_ISL_7337457, EPI_ISL_7337458, EPI_ISL_7337459, EPI_ISL_7337460, EPI_ISL_7337461, EPI_ISL_7337462, EPI_ISL_7456431			
see above	Helen Joseph Laboratory	National Institute for Communicable Diseases of the National Health Laboratory Service	Amoako DG; Bhiman JN; Everatt J; Ismail A; Mahlangu B; Mnguni A; Mohale T; Ntuli N; Scheepers C; Wolter N
EPI_ISL_3859862	Instituto Nacional de Investigação em Saúde	CERI, Centre for Epidemic Response and Innvoation, Stellenbosch University and KRISP, KZN Research Innovation and Sequencing Platform, UKZN.	Afonso P; David K; Emmanuel SJ; Freitas RH; Giandhari J; Inglês L; Lutucuta S; Miranda J; Morais J; Mufinda M; Naidoo Y; Neto Z; Paulo A Carralero RR Paixão JP; Pereira A; Pillay S; Tegally H; Wilkinson E; de Oliveira T
EPI_ISL_2271953, EPI_ISL_2271955, EPI_ISL_2271956, EPI_ISL_2271957, EPI_ISL_2271964, EPI_ISL_2271965, EPI_ISL_2271971, EPI_ISL_2271977, EPI_ISL_2271978, EPI_ISL_2271981, EPI_ISL_2271984, EPI_ISL_2271986, EPI_ISL_2271987, EPI_ISL_2271988, EPI_ISL_2271989, EPI_ISL_2271994, EPI_ISL_2272000, EPI_ISL_2272004, EPI_ISL_2272018			
see above	Joubert Clinic	National Institute for Communicable Diseases of the National Health Laboratory Service	Amoako DG; Bhiman JN; Ismail A; Mahlangu B; Mohale T; Ntuli N; Scheepers C
EPI_ISL_3342332, EPI_ISL_3342333, EPI_ISL_3342334, EPI_ISL_3342335, EPI_ISL_3342337, EPI_ISL_3342341, EPI_ISL_3342343, EPI_ISL_3342345, EPI_ISL_3342349			
see above	Jubilee laboratory	National Institute for Communicable Diseases of the National Health Laboratory Service	Amoako DG; Bhiman JN; Everatt J; Ismail A; Mahlangu B; Mnguni A; Mohale T; Ntuli N; Scheepers C
EPI_ISL_2382224, EPI_ISL_3024573, EPI_ISL_3024574, EPI_ISL_8182807	KIMBERLEY LABORATORY	National Institute for Communicable Diseases of the National Health Laboratory Service	Amoako DG; Bhiman JN; Everatt J; Ismail A; Kekana D; Mahlangu B; Mnguni A; Mohale T; Ntuli N; Scheepers C; Wolter N
EPI_ISL_5510405	KOOKRUS PHC-B	National Institute for Communicable Diseases of the National Health Laboratory Service	Amoako DG; Bhiman JN; Everatt J; Ismail A; Mahlangu B; Mohale T; Ntuli N; Scheepers C
EPI_ISL_2694614, EPI_ISL_2694615, EPI_ISL_2695512, EPI_ISL_2695513, EPI_ISL_2695523, EPI_ISL_2695524, EPI_ISL_5510279, EPI_ISL_5510281, EPI_ISL_5510340, EPI_ISL_5510343, EPI_ISL_5510488			
see above	KOPANONG	National Institute for Communicable Diseases of the National Health Laboratory Service	Amoako DG; Bhiman JN; Everatt J; Ismail A; Mahlangu B; Mnguni A; Mohale T; Ntuli N; Scheepers C
EPI_ISL_3101489, EPI_ISL_3101490, EPI_ISL_3101491, EPI_ISL_3101492, EPI_ISL_3101493, EPI_ISL_3101494, EPI_ISL_3101495, EPI_ISL_3101496, EPI_ISL_3101497, EPI_ISL_3101498, EPI_ISL_3101499, EPI_ISL_3101500, EPI_ISL_3101501, EPI_ISL_3101502, EPI_ISL_3101503, EPI_ISL_3101504, EPI_ISL_3101505, EPI_ISL_3101506, EPI_ISL_3101507, EPI_ISL_3101508, EPI_ISL_3101509, EPI_ISL_3101510, EPI_ISL_3101511, EPI_ISL_3101512, EPI_ISL_3101513, EPI_ISL_3101514, EPI_ISL_3101515, EPI_ISL_3101516, EPI_ISL_3101517, EPI_ISL_3101518, EPI_ISL_3101519, EPI_ISL_3101520, EPI_ISL_3101521, EPI_ISL_3101522, EPI_ISL_3101523, EPI_ISL_3101524, EPI_ISL_3101525, EPI_ISL_3101526, EPI_ISL_3101527, EPI_ISL_3101528, EPI_ISL_3101529, EPI_ISL_3101530, EPI_ISL_3101531, EPI_ISL_3101532,			

EPI_ISL_3101533, EPI_ISL_3101534, EPI_ISL_3101633, EPI_ISL_3101634, EPI_ISL_3149299, EPI_ISL_3149300, EPI_ISL_3149301, EPI_ISL_3149302, EPI_ISL_3149304

see above	KOPANONG HOSPITAL	National Institute for Communicable Diseases of the National Health Laboratory Service	Amoako DG; Bhiman JN; Everatt J; Ismail A; Mahlangu B; Mnguni A; Mohale T; Ntuli N; Scheepers C

EPI_ISL_2382260, EPI_ISL_2382261, EPI_ISL_2382262, EPI_ISL_2382263, EPI_ISL_2382264, EPI_ISL_2382265, EPI_ISL_2382266, EPI_ISL_2582987, EPI_ISL_2582990, EPI_ISL_2582993, EPI_ISL_2582996, EPI_ISL_2582999, EPI_ISL_2583001, EPI_ISL_2583005, EPI_ISL_2662662, EPI_ISL_2662663, EPI_ISL_2662664, EPI_ISL_2695759, EPI_ISL_2695760, EPI_ISL_2695761, EPI_ISL_2895766, EPI_ISL_2895767, EPI_ISL_2895768, EPI_ISL_2895769, EPI_ISL_2895770, EPI_ISL_2895771, EPI_ISL_2895799, EPI_ISL_2895800, EPI_ISL_2942283, EPI_ISL_3024643, EPI_ISL_3024644, EPI_ISL_3024645, EPI_ISL_3236955, EPI_ISL_3236956, EPI_ISL_3237104, EPI_ISL_3237105, EPI_ISL_3237106, EPI_ISL_3237254, EPI_ISL_3342222, EPI_ISL_3342223, EPI_ISL_3342224, EPI_ISL_3342225, EPI_ISL_3342226, EPI_ISL_3342227, EPI_ISL_3342228, EPI_ISL_3342229, EPI_ISL_3342230, EPI_ISL_3342231, EPI_ISL_3342232, EPI_ISL_3342233, EPI_ISL_3342234, EPI_ISL_3342235, EPI_ISL_3342236, EPI_ISL_3342237, EPI_ISL_3342238, EPI_ISL_3342239, EPI_ISL_3342240, EPI_ISL_3342241, EPI_ISL_3342242, EPI_ISL_3342243, EPI_ISL_3342244, EPI_ISL_3342245, EPI_ISL_3342246, EPI_ISL_3342248, EPI_ISL_3342249, EPI_ISL_3342250, EPI_ISL_3342251, EPI_ISL_3342252, EPI_ISL_3342253, EPI_ISL_3342254, EPI_ISL_3342256, EPI_ISL_3342422, EPI_ISL_3411463, EPI_ISL_3411466, EPI_ISL_3411467, EPI_ISL_3411469, EPI_ISL_3411770, EPI_ISL_3411810, EPI_ISL_3411811, EPI_ISL_3411812, EPI_ISL_3411813, EPI_ISL_3411814, EPI_ISL_3411815, EPI_ISL_3411816, EPI_ISL_3451255, EPI_ISL_3451256, EPI_ISL_3451491, EPI_ISL_3451492, EPI_ISL_3451493, EPI_ISL_3451494, EPI_ISL_3451495, EPI_ISL_3451496, EPI_ISL_3451497, EPI_ISL_3451498, EPI_ISL_3451499, EPI_ISL_3451500, EPI_ISL_3451501, EPI_ISL_3451502, EPI_ISL_3451503, EPI_ISL_3643814, EPI_ISL_3643821, EPI_ISL_3643830, EPI_ISL_3643833, EPI_ISL_3643840, EPI_ISL_3643846, EPI_ISL_3643870, EPI_ISL_3643876, EPI_ISL_3643877, EPI_ISL_3643887, EPI_ISL_3643889, EPI_ISL_3643900, EPI_ISL_3643901, EPI_ISL_3643910, EPI_ISL_3643911, EPI_ISL_3643912, EPI_ISL_3643914, EPI_ISL_3643917, EPI_ISL_3643924, EPI_ISL_3643926, EPI_ISL_3643932, EPI_ISL_3643933, EPI_ISL_3643934, EPI_ISL_3643950, EPI_ISL_3643960, EPI_ISL_3643961, EPI_ISL_3643963, EPI_ISL_3643966, EPI_ISL_3643969, EPI_ISL_3643974, EPI_ISL_3643980, EPI_ISL_3643981, EPI_ISL_3643982, EPI_ISL_3643986, EPI_ISL_3643987, EPI_ISL_3644019, EPI_ISL_3644020, EPI_ISL_3644021, EPI_ISL_3644022, EPI_ISL_3644023, EPI_ISL_3644024, EPI_ISL_3644025, EPI_ISL_3644026, EPI_ISL_3644027, EPI_ISL_3644028, EPI_ISL_3644029, EPI_ISL_5416274, EPI_ISL_5416342, EPI_ISL_6202362, EPI_ISL_6202425, EPI_ISL_6202743, EPI_ISL_6202820, EPI_ISL_6327770, EPI_ISL_6327771, EPI_ISL_6327848, EPI_ISL_6900144, EPI_ISL_6900145, EPI_ISL_6900146, EPI_ISL_6900147, EPI_ISL_6900148, EPI_ISL_6900149

see above	KOPANONG LABORATORY	National Institute for Communicable Diseases of the National Health Laboratory Service	Amoako DG; Bhiman JN; Everatt J; Ismail A; Mahlangu B; Mnguni A; Mohale T; Ntuli N; Scheepers C
EPI_ISL_5510467, EPI_ISL_5510468, EPI_ISL_5510482	Kathleen Subramoney	National Institute for Communicable Diseases of the National Health Laboratory Service	Amoako DG; Bhiman JN; Everatt J; Ismail A; Mahlangu B; Mnguni A; Mohale T; Ntuli N; Scheepers C
EPI_ISL_5510359, EPI_ISL_5510402, EPI_ISL_5510438, EPI_ISL_5510441, EPI_ISL_5510465	Kopanong	National Institute for Communicable Diseases of the National Health Laboratory Service	Amoako DG; Bhiman JN; Everatt J; Ismail A; Mahlangu B; Mnguni A; Mohale T; Ntuli N; Scheepers C

EPI_ISL_5917867, EPI_ISL_5917931, EPI_ISL_5917976, EPI_ISL_5917993, EPI_ISL_5918007, EPI_ISL_5918012, EPI_ISL_5918148, EPI_ISL_7337381, EPI_ISL_7337385, EPI_ISL_7337386, EPI_ISL_7337387, EPI_ISL_7337388, EPI_ISL_7456413, EPI_ISL_7456414, EPI_ISL_7456415, EPI_ISL_7456416, EPI_ISL_7456418, EPI_ISL_7456429

see above	Kopanong Laboratory	National Institute for Communicable Diseases of the National Health Laboratory Service	Amoako DG; Bhiman JN; Everatt J; Ismail A; Mahlangu B; Mnguni A; Mohale T; Ntuli N; Scheepers C; Wolter N

EPI_ISL_2463301, EPI_ISL_2463302, EPI_ISL_2463303, EPI_ISL_2463304, EPI_ISL_2463305, EPI_ISL_2463306, EPI_ISL_2463307, EPI_ISL_2463308, EPI_ISL_2463309, EPI_ISL_2463310, EPI_ISL_3236957, EPI_ISL_3236989, EPI_ISL_3236990, EPI_ISL_3236991, EPI_ISL_3236992, EPI_ISL_3237107, EPI_ISL_3237108, EPI_ISL_3237109, EPI_ISL_3237110, EPI_ISL_3237111, EPI_ISL_3237112, EPI_ISL_3237113, EPI_ISL_3237167, EPI_ISL_3237168, EPI_ISL_3237169, EPI_ISL_3237170, EPI_ISL_3237171, EPI_ISL_3237172, EPI_ISL_3237258, EPI_ISL_3643990, EPI_ISL_6647956, EPI_ISL_6647957, EPI_ISL_6647958, EPI_ISL_6647959, EPI_ISL_6647960, EPI_ISL_6647961, EPI_ISL_6647962, EPI_ISL_6698792, EPI_ISL_6704863, EPI_ISL_6704864, EPI_ISL_6704865, EPI_ISL_6704866, EPI_ISL_6704867, EPI_ISL_6704868, EPI_ISL_6704869, EPI_ISL_6704870, EPI_ISL_6704871, EPI_ISL_6704872, EPI_ISL_6704873, EPI_ISL_6704874, EPI_ISL_6704875, EPI_ISL_6704876, EPI_ISL_7701115, EPI_ISL_7701117, EPI_ISL_7701120, EPI_ISL_7701122, EPI_ISL_7701125, EPI_ISL_7701127, EPI_ISL_7701129, EPI_ISL_7701131, EPI_ISL_7701134, EPI_ISL_7701136, EPI_ISL_7701138

see above	LANCET LABORATORY	National Institute for Communicable Diseases of the National Health Laboratory Service	Amoako DG; Bhiman JN; Everatt J; Glass A; Ismail A; Mahlangu B; Mnguni A; Mohale T; Ntuli N; Scheepers C; Sisonke Team; Viana R; Wolter N

EPI_ISL_2433705, EPI_ISL_2433706, EPI_ISL_2433707, EPI_ISL_2433708, EPI_ISL_2433709, EPI_ISL_2433710, EPI_ISL_2433711, EPI_ISL_2433713, EPI_ISL_2433716, EPI_ISL_2433717, EPI_ISL_2433719, EPI_ISL_2433720, EPI_ISL_2433721, EPI_ISL_2433722, EPI_ISL_2433723, EPI_ISL_2433724, EPI_ISL_2433850, EPI_ISL_2433851, EPI_ISL_2433852, EPI_ISL_2433853, EPI_ISL_2433854, EPI_ISL_2433855, EPI_ISL_2433856, EPI_ISL_2433857, EPI_ISL_2433858, EPI_ISL_2433859, EPI_ISL_2433860, EPI_ISL_2433864, EPI_ISL_2433865, EPI_ISL_2433866, EPI_ISL_2433870, EPI_ISL_2433871, EPI_ISL_2433872, EPI_ISL_2433873, EPI_ISL_2433874, EPI_ISL_2433875, EPI_ISL_2433876, EPI_ISL_2433877, EPI_ISL_2433878, EPI_ISL_2433879, EPI_ISL_2433880, EPI_ISL_2433881, EPI_ISL_2433882, EPI_ISL_2433883, EPI_ISL_2433884, EPI_ISL_2433885, EPI_ISL_2433886, EPI_ISL_2433887, EPI_ISL_2433888, EPI_ISL_2433889, EPI_ISL_2433890, EPI_ISL_2433891, EPI_ISL_2433892, EPI_ISL_2433893, EPI_ISL_2433895, EPI_ISL_2433896, EPI_ISL_2433897, EPI_ISL_2433899, EPI_ISL_2433900, EPI_ISL_2433901, EPI_ISL_2433903, EPI_ISL_2433904, EPI_ISL_2433905, EPI_ISL_2433909, EPI_ISL_2433910, EPI_ISL_2433911, EPI_ISL_2433912, EPI_ISL_2433913, EPI_ISL_2433914, EPI_ISL_2433915, EPI_ISL_2433916, EPI_ISL_2433918, EPI_ISL_2433919, EPI_ISL_2433920, EPI_ISL_2433922, EPI_ISL_2433923, EPI_ISL_2433924, EPI_ISL_2433925, EPI_ISL_2443995, EPI_ISL_2443996

see above	LANCET, LABORATORIES	National Institute for Communicable Diseases of the National Health Laboratory Service	Amoako DG; Bhiman JN; Ismail A; Mahlangu B; Mohale T; Ntuli N; Scheepers C
EPI_ISL_2828731, EPI_ISL_2828732, EPI_ISL_2828733, EPI_ISL_5510283, EPI_ISL_5510348	Lancet	National Institute for Communicable Diseases of the National Health Laboratory Service	Amoako DG; Bhiman JN; Everatt J; Ismail A; Mahlangu B; Mnguni A; Mohale T; Ntuli N; Scheepers C

EPI_ISL_2013048, EPI_ISL_2013049, EPI_ISL_2013050, EPI_ISL_2013051, EPI_ISL_2013052, EPI_ISL_2013053, EPI_ISL_6913991, EPI_ISL_6913992, EPI_ISL_6913993, EPI_ISL_6913994, EPI_ISL_6913995, EPI_ISL_6913996, EPI_ISL_6913997, EPI_ISL_6913998, EPI_ISL_6913999, EPI_ISL_6914000, EPI_ISL_6914001, EPI_ISL_6914002, EPI_ISL_6914003, EPI_ISL_6914004, EPI_ISL_6914005, EPI_ISL_6914006, EPI_ISL_6914007

see above	Lancet Laboratories	National Institute for Communicable Diseases of the National Health Laboratory Service	Amoako DG; Bhiman JN; Everatt J; Glass A; Ismail A; Mahlangu B; Mnguni A; Mohale T; Ntuli N; Scheepers C; Viana R; Wolter N

EPI_ISL_2984866, EPI_ISL_2984867, EPI_ISL_2984868, EPI_ISL_2984869, EPI_ISL_2984870, EPI_ISL_2984871, EPI_ISL_2984872, EPI_ISL_2984873, EPI_ISL_3073888, EPI_ISL_3073889, EPI_ISL_3073890, EPI_ISL_3073891, EPI_ISL_3073892, EPI_ISL_3073893, EPI_ISL_3073894, EPI_ISL_3073895, EPI_ISL_3073896, EPI_ISL_3073897, EPI_ISL_3073898, EPI_ISL_3073899, EPI_ISL_3073900, EPI_ISL_3073901, EPI_ISL_3073910, EPI_ISL_3073911, EPI_ISL_3074049, EPI_ISL_3074050, EPI_ISL_3074051, EPI_ISL_3074052, EPI_ISL_3074053, EPI_ISL_3074054, EPI_ISL_3074055, EPI_ISL_3131775, EPI_ISL_3149307

see above	Lancet Laboratory	National Institute for Communicable Diseases of the National Health Laboratory Service	Amoako DG; Bhiman JN; Everatt J; Ismail A; Mahlangu B; Mnguni A; Mohale T; Ntuli N; Scheepers C
EPI_ISL_3024571, EPI_ISL_3024572, EPI_ISL_3237114, EPI_ISL_3237115	MANKWENG LABORATORY	National Institute for Communicable Diseases of the National Health Laboratory Service	Amoako DG; Bhiman JN; Everatt J; Ismail A; Mahlangu B; Mnguni A; Mohale T; Ntuli N; Scheepers C
EPI_ISL_3024682	MILPARK HOSPITAL	National Institute for Communicable Diseases of the National Health Laboratory Service	Amoako DG; Bhiman JN; Everatt J; Ismail A; Mahlangu B; Mnguni A; Mohale T; Ntuli N; Scheepers C; Sisonke Team
EPI_ISL_2271968	Matikwana Hospital	National Institute for Communicable Diseases of the National Health Laboratory Service	Amoako DG; Bhiman JN; Ismail A; Mahlangu B; Mohale T; Ntuli N; Scheepers C
EPI_ISL_5918058, EPI_ISL_5918154, EPI_ISL_6913986, EPI_ISL_7456404	Mobile Labs Gauteng	National Institute for Communicable Diseases of the National Health Laboratory Service	Amoako DG; Bhiman JN; Everatt J; Ismail A; Mahlangu B; Mnguni A; Mohale T; Ntuli N; Scheepers C; Wolter N

EPI_ISL_2710249, EPI_ISL_2710250, EPI_ISL_2710251, EPI_ISL_2710252, EPI_ISL_2710253, EPI_ISL_2710254, EPI_ISL_2710255, EPI_ISL_2710256, EPI_ISL_2710257, EPI_ISL_2710258, EPI_ISL_2710259, EPI_ISL_2710260, EPI_ISL_2710261, EPI_ISL_2710262, EPI_ISL_2710263, EPI_ISL_2710264, EPI_ISL_2710265, EPI_ISL_2710266, EPI_ISL_2710267, EPI_ISL_2710268, EPI_ISL_2710269, EPI_ISL_2710270, EPI_ISL_2710271, EPI_ISL_2710272, EPI_ISL_2710273, EPI_ISL_2710274, EPI_ISL_2710275, EPI_ISL_2710276, EPI_ISL_2710277, EPI_ISL_2710278, EPI_ISL_2710279, EPI_ISL_2710280, EPI_ISL_2710281, EPI_ISL_2710282, EPI_ISL_2710283, EPI_ISL_2710284, EPI_ISL_2710285, EPI_ISL_2710286, EPI_ISL_2710287, EPI_ISL_2710288, EPI_ISL_2710289, EPI_ISL_2710290, EPI_ISL_2710291, EPI_ISL_2710292, EPI_ISL_2710293, EPI_ISL_2710294, EPI_ISL_2710295, EPI_ISL_2710296, EPI_ISL_2710297, EPI_ISL_2710298, EPI_ISL_2710299, EPI_ISL_2710300, EPI_ISL_2710301, EPI_ISL_2710302, EPI_ISL_2710303, EPI_ISL_2710304, EPI_ISL_2710305, EPI_ISL_2726854, EPI_ISL_2726855, EPI_ISL_2726856, EPI_ISL_2770439, EPI_ISL_2770440, EPI_ISL_2770441, EPI_ISL_2770442, EPI_ISL_2770443, EPI_ISL_2770444, EPI_ISL_2770445, EPI_ISL_2770446, EPI_ISL_2770447, EPI_ISL_2770448, EPI_ISL_2770449, EPI_ISL_2770450, EPI_ISL_2770451, EPI_ISL_2770452, EPI_ISL_2770453, EPI_ISL_2770454, EPI_ISL_2770455, EPI_ISL_2770456, EPI_ISL_2770457, EPI_ISL_2770458, EPI_ISL_2770459, EPI_ISL_2770460, EPI_ISL_2770461, EPI_ISL_2770462, EPI_ISL_2770463, EPI_ISL_2770464, EPI_ISL_2770465, EPI_ISL_2770466, EPI_ISL_2770467, EPI_ISL_2770468, EPI_ISL_2770469, EPI_ISL_2770470, EPI_ISL_2770471, EPI_ISL_2770472, EPI_ISL_2770473, EPI_ISL_2770474, EPI_ISL_2770475, EPI_ISL_2770476, EPI_ISL_2770477, EPI_ISL_2770478, EPI_ISL_2770479, EPI_ISL_2770480, EPI_ISL_2770481, EPI_ISL_2770482, EPI_ISL_2770483, EPI_ISL_2770484, EPI_ISL_2770485, EPI_ISL_2770486, EPI_ISL_2770487, EPI_ISL_2770488, EPI_ISL_4572817, EPI_ISL_4572818, EPI_ISL_4572819, EPI_ISL_4572820, EPI_ISL_4572821, EPI_ISL_4572822, EPI_ISL_4572823, EPI_ISL_4572824, EPI_ISL_4572825, EPI_ISL_4572826, EPI_ISL_4572827, EPI_ISL_4572828, EPI_ISL_4572831, EPI_ISL_4572832, EPI_ISL_4572833, EPI_ISL_4572834, EPI_ISL_4572835, EPI_ISL_4572836, EPI_ISL_4572840, EPI_ISL_4572842, EPI_ISL_4572843, EPI_ISL_4572844, EPI_ISL_4572845, EPI_ISL_4572846, EPI_ISL_4572847, EPI_ISL_4572848, EPI_ISL_4572849, EPI_ISL_4572850, EPI_ISL_4572851, EPI_ISL_4572852, EPI_ISL_4572853, EPI_ISL_4572854, EPI_ISL_4572855, EPI_ISL_4572856, EPI_ISL_4572857, EPI_ISL_4572860, EPI_ISL_4572861, EPI_ISL_4572862, EPI_ISL_4572864, EPI_ISL_4572865, EPI_ISL_4572866, EPI_ISL_4572867

see above	NHLS Charlotte Maxeke Johannesburg Academic Hospital and the University of the Witwatersrand	KRISP, KZN Research Innovation and Sequencing Platform	Bulelani Manene; Florette Treurnicht; Giandhari Jennifer; Kathleen Subramoney; Naidoo Yeshnee; Pillay Sureshnee; San James; Sisonke; Tegally Houriiyah; Tshabuila Derek; Wilkinson Eduan; Yajna Ramphal; de Oliveira Tulio

EPI_ISL_2841643, EPI_ISL_2841644, EPI_ISL_2841645, EPI_ISL_2841646, EPI_ISL_2841647, EPI_ISL_2841648, EPI_ISL_2841649, EPI_ISL_3132494, EPI_ISL_3132495, EPI_ISL_3132496, EPI_ISL_3132497, EPI_ISL_3132498, EPI_ISL_3132499, EPI_ISL_3132500, EPI_ISL_3132501, EPI_ISL_3132502, EPI_ISL_3132503, EPI_ISL_3132504, EPI_ISL_3132505, EPI_ISL_3132506, EPI_ISL_3132507, EPI_ISL_3132508, EPI_ISL_3132509, EPI_ISL_3132510, EPI_ISL_3132511, EPI_ISL_3132512, EPI_ISL_3132513, EPI_ISL_3132514, EPI_ISL_3132515, EPI_ISL_3132516, EPI_ISL_3132517, EPI_ISL_3132518, EPI_ISL_3132519, EPI_ISL_3132520, EPI_ISL_3132521, EPI_ISL_3132522, EPI_ISL_3132523, EPI_ISL_3132524, EPI_ISL_3132525, EPI_ISL_3132526, EPI_ISL_3132527, EPI_ISL_3132528, EPI_ISL_3132529, EPI_ISL_3132530, EPI_ISL_3132531, EPI_ISL_3132532, EPI_ISL_3132533, EPI_ISL_3132534, EPI_ISL_3132535, EPI_ISL_3132536, EPI_ISL_3132537, EPI_ISL_3132538, EPI_ISL_3132539, EPI_ISL_3132540, EPI_ISL_3132541, EPI_ISL_3132542, EPI_ISL_3132543, EPI_ISL_3132544, EPI_ISL_3132545, EPI_ISL_3132546, EPI_ISL_3132547, EPI_ISL_3132548, EPI_ISL_3132549, EPI_ISL_3132550, EPI_ISL_3132551, EPI_ISL_3132552, EPI_ISL_3132553, EPI_ISL_3132554, EPI_ISL_3132555, EPI_ISL_3132556, EPI_ISL_3132557, EPI_ISL_3132558, EPI_ISL_3132559, EPI_ISL_3132560, EPI_ISL_3132561, EPI_ISL_3132562, EPI_ISL_3132563, EPI_ISL_3132564, EPI_ISL_3132565, EPI_ISL_3132566, EPI_ISL_3132567, EPI_ISL_3132568, EPI_ISL_3132569, EPI_ISL_3132570, EPI_ISL_3132571, EPI_ISL_3132572, EPI_ISL_3132573, EPI_ISL_3132623, EPI_ISL_3132624, EPI_ISL_3132625, EPI_ISL_3132626, EPI_ISL_3132627

see above	NHLS Charlotte Maxeke Johannesburg Academic Hospital and the University of the Witwatersrand	KRISP, KZn Research Innovation and Sequencing Platform	Bulelani Manene; Florette Treurnicht; Giandhari Jennifer; Kathleen Subramoney; Naidoo Yeshnee; Pillay Sureshnee; San James; Tegally Houriiyah; Tshabuila Derek; Wilkinson Eduan; Yajna Ramphal; de Oliveira Tulio

EPI_ISL_4473159, EPI_ISL_4473163, EPI_ISL_4473165, EPI_ISL_4473166, EPI_ISL_4473174, EPI_ISL_4473175, EPI_ISL_4473177, EPI_ISL_4473181, EPI_ISL_4473183, EPI_ISL_4473186, EPI_ISL_4473189, EPI_ISL_4473208, EPI_ISL_4473211, EPI_ISL_4473218, EPI_ISL_4473219, EPI_ISL_4473221, EPI_ISL_5098656, EPI_ISL_5098663, EPI_ISL_5098665, EPI_ISL_5098669, EPI_ISL_5098679, EPI_ISL_5098684, EPI_ISL_5098692, EPI_ISL_5098700, EPI_ISL_5098706, EPI_ISL_5098710, EPI_ISL_5098711, EPI_ISL_5098717, EPI_ISL_5098720, EPI_ISL_5098726, EPI_ISL_5098736, EPI_ISL_5098743, EPI_ISL_5098751, EPI_ISL_5098759, EPI_ISL_5098807, EPI_ISL_5098812, EPI_ISL_5098820, EPI_ISL_5098828, EPI_ISL_5098833, EPI_ISL_5098838, EPI_ISL_5098847, EPI_ISL_5098853, EPI_ISL_5098857, EPI_ISL_5098864, EPI_ISL_5098890, EPI_ISL_5098899, EPI_ISL_5098906, EPI_ISL_5098927, EPI_ISL_5098931, EPI_ISL_5098934, EPI_ISL_5098964, EPI_ISL_5098971, EPI_ISL_5098977, EPI_ISL_5098981, EPI_ISL_5098993, EPI_ISL_5099000, EPI_ISL_5099020, EPI_ISL_5099027, EPI_ISL_5099032, EPI_ISL_5099041, EPI_ISL_5099047, EPI_ISL_5099057, EPI_ISL_5099071, EPI_ISL_5099077, EPI_ISL_5099085, EPI_ISL_5099154, EPI_ISL_5099155, EPI_ISL_5099171, EPI_ISL_5099172, EPI_ISL_5099173, EPI_ISL_5099204, EPI_ISL_5099216, EPI_ISL_5429023, EPI_ISL_5429024, EPI_ISL_5429025, EPI_ISL_5429026, EPI_ISL_5429027, EPI_ISL_5429034, EPI_ISL_5429035, EPI_ISL_5429036, EPI_ISL_5429038, EPI_ISL_5429039, EPI_ISL_5429040, EPI_ISL_5429041, EPI_ISL_5429042, EPI_ISL_5429043, EPI_ISL_5429044, EPI_ISL_5429045, EPI_ISL_5429049, EPI_ISL_6422264, EPI_ISL_6422265, EPI_ISL_6422266, EPI_ISL_6422267, EPI_ISL_6422268, EPI_ISL_6422269, EPI_ISL_6422305, EPI_ISL_6422306, EPI_ISL_6422307, EPI_ISL_6422308, EPI_ISL_6422309, EPI_ISL_6422310, EPI_ISL_6422311, EPI_ISL_6422312, EPI_ISL_6422313, EPI_ISL_6422314, EPI_ISL_6422315, EPI_ISL_6422316, EPI_ISL_6422317, EPI_ISL_6422318

see above	NHLS Charlotte Maxeke Johannesburg Hospital	CERI, Centre for Epidemic Response and Innvoation, Stellenbosch University and KRISP, KZN Research Innovation and Sequencing Platform, UKZN.	Emmanuel SJ; Florette Treurnicht; Giandhari J; Giandhari Jennifer; Kathleen Subramoney; Naidoo Y; Naidoo Yeshnee; Pillay S; Pillay Sureshnee; San James; Tegally H; Tegally Houriiyah; Tshiabuila Derek; Upasana Ramphal; Wilkinson E; Wilkinson Eduan; Yajna Ramphal; de Oliveira T; de Oliveira Tulio

EPI_ISL_3728977, EPI_ISL_3728981, EPI_ISL_3729063, EPI_ISL_3729085, EPI_ISL_3729089, EPI_ISL_3729093, EPI_ISL_3729097, EPI_ISL_3729110, EPI_ISL_3729113, EPI_ISL_3729114, EPI_ISL_3729116, EPI_ISL_3729117, EPI_ISL_3729119, EPI_ISL_3729120, EPI_ISL_3729121, EPI_ISL_3729122, EPI_ISL_3729123, EPI_ISL_3729127, EPI_ISL_3729128, EPI_ISL_3729129, EPI_ISL_3729130, EPI_ISL_3729133, EPI_ISL_3729134, EPI_ISL_3729135, EPI_ISL_3729136, EPI_ISL_3729137, EPI_ISL_3729139, EPI_ISL_3729140, EPI_ISL_3729141, EPI_ISL_3729143, EPI_ISL_3729144, EPI_ISL_3729148, EPI_ISL_3729149, EPI_ISL_3729151, EPI_ISL_3729152, EPI_ISL_3729154, EPI_ISL_3729157, EPI_ISL_3729161, EPI_ISL_3729168, EPI_ISL_3729172, EPI_ISL_3729176, EPI_ISL_3729180, EPI_ISL_3729188, EPI_ISL_3729199, EPI_ISL_3729223, EPI_ISL_3729230, EPI_ISL_3729239, EPI_ISL_3729243, EPI_ISL_3729247, EPI_ISL_3729250, EPI_ISL_3859855, EPI_ISL_3859857, EPI_ISL_3859866, EPI_ISL_3859868, EPI_ISL_3859869, EPI_ISL_3859873, EPI_ISL_3859880, EPI_ISL_3859881, EPI_ISL_3859884, EPI_ISL_3859885, EPI_ISL_3859887, EPI_ISL_3859888, EPI_ISL_3859890, EPI_ISL_3859894, EPI_ISL_3859926, EPI_ISL_3859930, EPI_ISL_3859933, EPI_ISL_3859940, EPI_ISL_3859942, EPI_ISL_3859944, EPI_ISL_3859945, EPI_ISL_3859950, EPI_ISL_3859954, EPI_ISL_3859958, EPI_ISL_3859962, EPI_ISL_3859966, EPI_ISL_3859969, EPI_ISL_3859971, EPI_ISL_3859972, EPI_ISL_3859974, EPI_ISL_3859978, EPI_ISL_3859982, EPI_ISL_3859986, EPI_ISL_3859996, EPI_ISL_3859998, EPI_ISL_3859999, EPI_ISL_3860010, EPI_ISL_3860012, EPI_ISL_3860014, EPI_ISL_3860015, EPI_ISL_3860037, EPI_ISL_3860040, EPI_ISL_3860047, EPI_ISL_3860057, EPI_ISL_3860059, EPI_ISL_3860060, EPI_ISL_3860064, EPI_ISL_3860068, EPI_ISL_3860070, EPI_ISL_3860076, EPI_ISL_3860078, EPI_ISL_3860090, EPI_ISL_3860098, EPI_ISL_3860100, EPI_ISL_3860110, EPI_ISL_3860114, EPI_ISL_3860124, EPI_ISL_3860128, EPI_ISL_3860130, EPI_ISL_3860132, EPI_ISL_3860133, EPI_ISL_3860135, EPI_ISL_3860137, EPI_ISL_3860141, EPI_ISL_3860143, EPI_ISL_3860145, EPI_ISL_3860147, EPI_ISL_3860148, EPI_ISL_3860150, EPI_ISL_3860152, EPI_ISL_3860154, EPI_ISL_3860155, EPI_ISL_3860157, EPI_ISL_3860159, EPI_ISL_3860161, EPI_ISL_3860163, EPI_ISL_3860165, EPI_ISL_3860167, EPI_ISL_3860168, EPI_ISL_4572296, EPI_ISL_4572297, EPI_ISL_4572298, EPI_ISL_4572299, EPI_ISL_4572300, EPI_ISL_4572301, EPI_ISL_4572302, EPI_ISL_4572303, EPI_ISL_4572304, EPI_ISL_4572305, EPI_ISL_4572306, EPI_ISL_4572307, EPI_ISL_4572308, EPI_ISL_4572320, EPI_ISL_4572321, EPI_ISL_4572325, EPI_ISL_4572327, EPI_ISL_4572328, EPI_ISL_4572337, EPI_ISL_4572340, EPI_ISL_4572341, EPI_ISL_4572344, EPI_ISL_4572345, EPI_ISL_4572346, EPI_ISL_4572347, EPI_ISL_4572349, EPI_ISL_4572350, EPI_ISL_4572352, EPI_ISL_4572355, EPI_ISL_4572363, EPI_ISL_4572371, EPI_ISL_4572372, EPI_ISL_4572375, EPI_ISL_4572376, EPI_ISL_4572381, EPI_ISL_4572382, EPI_ISL_4572383, EPI_ISL_4572384, EPI_ISL_4572386, EPI_ISL_4572389

see above	NHLS Charlotte Maxeke Johannesburg Hospital	KRISP, KZN Research Innovation and Sequencing Platform	Emmanuel S; Florette Treurnicht; Giandhari J; Giandhari Jennifer; Kathleen Subramoney; Naidoo Yeshnee; Pillay S; Pillay Sureshnee; San James; Tegally H; Tegally Houriiyah; Tshabuila Derek; Wilkinson E; Wilkinson Eduan; Yajna Ramphal; de Oliveira T; de Oliveira Tulio

EPI_ISL_3722215, EPI_ISL_3722216, EPI_ISL_3722217, EPI_ISL_3722218, EPI_ISL_3722219, EPI_ISL_3722220, EPI_ISL_3722221, EPI_ISL_3722222, EPI_ISL_3722223, EPI_ISL_3722224, EPI_ISL_3722225, EPI_ISL_3722226, EPI_ISL_3722227, EPI_ISL_3722228, EPI_ISL_3722229, EPI_ISL_3722230, EPI_ISL_3722231, EPI_ISL_3722232, EPI_ISL_3722233, EPI_ISL_3722234, EPI_ISL_3722235, EPI_ISL_3722236,

EPI_ISL_3722237, EPI_ISL_3722238, EPI_ISL_3722239, EPI_ISL_3722240, EPI_ISL_3722241, EPI_ISL_3722242, EPI_ISL_3722243, EPI_ISL_3722244, EPI_ISL_3722245, EPI_ISL_3722246, EPI_ISL_3722247, EPI_ISL_3722248, EPI_ISL_3722249, EPI_ISL_3722250, EPI_ISL_3722251, EPI_ISL_3722252, EPI_ISL_3722253, EPI_ISL_3722254, EPI_ISL_3722255, EPI_ISL_3722256, EPI_ISL_3722257, EPI_ISL_3722258, EPI_ISL_3722259, EPI_ISL_3722260, EPI_ISL_3722261, EPI_ISL_3722262, EPI_ISL_3722263, EPI_ISL_3722264, EPI_ISL_3722265, EPI_ISL_3722266, EPI_ISL_3722267, EPI_ISL_3722268, EPI_ISL_3722269, EPI_ISL_3722270, EPI_ISL_3722271, EPI_ISL_3722272, EPI_ISL_3722273, EPI_ISL_3722274, EPI_ISL_3722275, EPI_ISL_3722276, EPI_ISL_3722277, EPI_ISL_3722278, EPI_ISL_3799009, EPI_ISL_3799010, EPI_ISL_3799013, EPI_ISL_3799014, EPI_ISL_3799016, EPI_ISL_3799017, EPI_ISL_3799018, EPI_ISL_3799019, EPI_ISL_3799022, EPI_ISL_3799027, EPI_ISL_3799028, EPI_ISL_3799035, EPI_ISL_3799036, EPI_ISL_3799056, EPI_ISL_3799062, EPI_ISL_3799063, EPI_ISL_3799064, EPI_ISL_3799066, EPI_ISL_3799071, EPI_ISL_3799083, EPI_ISL_3799085, EPI_ISL_3799089, EPI_ISL_3799095, EPI_ISL_3799104, EPI_ISL_3799116, EPI_ISL_3799122, EPI_ISL_3799124, EPI_ISL_3799128, EPI_ISL_3799137, EPI_ISL_3799139, EPI_ISL_3799143, EPI_ISL_3799145, EPI_ISL_3799151, EPI_ISL_3799153, EPI_ISL_3799155, EPI_ISL_3799156, EPI_ISL_3799158, EPI_ISL_3799159, EPI_ISL_3799160, EPI_ISL_3799161, EPI_ISL_3799162, EPI_ISL_3799165, EPI_ISL_3799166, EPI_ISL_3799168, EPI_ISL_3799169, EPI_ISL_3799170, EPI_ISL_3799171, EPI_ISL_4121605, EPI_ISL_4121607, EPI_ISL_4121610, EPI_ISL_4121621, EPI_ISL_4121634, EPI_ISL_4121656, EPI_ISL_4121659, EPI_ISL_4121668, EPI_ISL_4121671, EPI_ISL_4121675, EPI_ISL_4121681, EPI_ISL_4121687, EPI_ISL_4121710, EPI_ISL_4121713, EPI_ISL_4121728, EPI_ISL_4121731, EPI_ISL_4121733, EPI_ISL_4121740, EPI_ISL_4121764, EPI_ISL_4121770, EPI_ISL_4121779, EPI_ISL_4121785, EPI_ISL_4336962, EPI_ISL_4336965, EPI_ISL_4336968, EPI_ISL_4336970			
see above	NHLS Charlotte Maxeke Johannesburg Hospital	KRISP, KZn Research Innovation and Sequencing Platform	Emmanuel S; Florette Treurnicht; Giandhari J; Giandhari Jennifer; Kathleen Subramoney; Naidoo Yeshnee; Pillay S; Pillay Sureshnee; San James; Tegally H; Tegally Houriiyah; Tshabuila Derek; Tshiabuila Derek; Wilkinson E; Wilkinson Eduan; Yajna Ramphal; de Oliveira T; de Oliveira Tulio
EPI_ISL_3447706, EPI_ISL_3447707, EPI_ISL_3447708, EPI_ISL_3447713, EPI_ISL_3447714, EPI_ISL_3447715, EPI_ISL_3447718, EPI_ISL_3447728, EPI_ISL_3447729, EPI_ISL_3447734, EPI_ISL_3447737, EPI_ISL_3447738, EPI_ISL_3447739, EPI_ISL_3447743, EPI_ISL_3447744, EPI_ISL_3447746, EPI_ISL_3447748, EPI_ISL_3447754, EPI_ISL_3447760, EPI_ISL_3447761, EPI_ISL_3447762, EPI_ISL_3447763, EPI_ISL_3447764, EPI_ISL_3447765, EPI_ISL_3447766, EPI_ISL_3447767, EPI_ISL_3447768, EPI_ISL_3447769, EPI_ISL_3447770, EPI_ISL_3447771, EPI_ISL_3447772, EPI_ISL_3447773, EPI_ISL_3447774, EPI_ISL_3447775, EPI_ISL_3447776, EPI_ISL_3447777, EPI_ISL_3447778, EPI_ISL_3447780, EPI_ISL_3447781, EPI_ISL_3447783, EPI_ISL_3447784, EPI_ISL_3447785, EPI_ISL_3447786, EPI_ISL_3447787, EPI_ISL_3447788, EPI_ISL_3447789, EPI_ISL_3447790, EPI_ISL_3447791, EPI_ISL_3447792, EPI_ISL_3447793, EPI_ISL_3447794, EPI_ISL_3447795, EPI_ISL_3447796, EPI_ISL_3447798, EPI_ISL_3447799, EPI_ISL_3447800, EPI_ISL_3447803, EPI_ISL_3447804, EPI_ISL_3447806, EPI_ISL_3447807, EPI_ISL_3447808, EPI_ISL_3447809			
see above	NHLS_JHB	KRISP, KZN Research Innovation and Sequencing Platform	Bulelani Manene; Florette Treurnicht; Giandhari Jennifer; Kathleen Subramoney; Naidoo Yeshnee; Pillay Sureshnee; San James; Tegally Houriiyah; Tshiabuila Derek; Wilkinson Eduan; Yajna Ramphal; de Oliveira Tulio
EPI_ISL_3261914, EPI_ISL_3261915, EPI_ISL_3261916, EPI_ISL_3261917, EPI_ISL_3261918, EPI_ISL_3261919, EPI_ISL_3261920, EPI_ISL_3261921, EPI_ISL_3261922, EPI_ISL_3261923			
see above	NHLS_JHB (Ship 4)	KRISP, KZN Research Innovation and Sequencing Platform	Giandhari Jennifer; Naidoo Yeshnee; Pillay Sureshnee; San James; Tegally Houriiyah; Tshabuila Derek; Wilkinson Eduan; Yajna Ramphal; de Oliveira Tulio
EPI_ISL_4253647	NICD staff	National Institute for Communicable Diseases of the National Health Laboratory Service	Amoako DG; Bhiman JN; Everatt J; Ismail A; Mahlangu B; Mnguni A; Mohale T; Ntuli N; Scheepers C; Sisonke Team
EPI_ISL_5510364	Natalspruit	National Institute for Communicable Diseases of the National Health Laboratory Service	Amoako DG; Bhiman JN; Everatt J; Ismail A; Mahlangu B; Mnguni A; Mohale T; Ntuli N; Scheepers C
EPI_ISL_1363124, EPI_ISL_1363125, EPI_ISL_1363126, EPI_ISL_1363127, EPI_ISL_1363128, EPI_ISL_1363129, EPI_ISL_1363130, EPI_ISL_1363131, EPI_ISL_1363132, EPI_ISL_1363133, EPI_ISL_1363134, EPI_ISL_1363135, EPI_ISL_1363136, EPI_ISL_1363137, EPI_ISL_1363138, EPI_ISL_1363139, EPI_ISL_1363140, EPI_ISL_1363141, EPI_ISL_1363142, EPI_ISL_1363143, EPI_ISL_1363144, EPI_ISL_1363145, EPI_ISL_1372562			
see above	National Health Laboratory Services	National Health Laboratory Services	Arshad Ismail; Florette Treurnicht; Kathleen Subramoney
EPI_ISL_6795188, EPI_ISL_6795189, EPI_ISL_6795190, EPI_ISL_6795191, EPI_ISL_6795192, EPI_ISL_6795193, EPI_ISL_6795194, EPI_ISL_7715346, EPI_ISL_7715347, EPI_ISL_7715348, EPI_ISL_7715349, EPI_ISL_7715350, EPI_ISL_7715351, EPI_ISL_7715352, EPI_ISL_7715353, EPI_ISL_7715354, EPI_ISL_7715355, EPI_ISL_7715356, EPI_ISL_7715357, EPI_ISL_7715358, EPI_ISL_7715359, EPI_ISL_7715360, EPI_ISL_7715361, EPI_ISL_7715362, EPI_ISL_7715363, EPI_ISL_7715364, EPI_ISL_7715365, EPI_ISL_7715366, EPI_ISL_7715367, EPI_ISL_7715368, EPI_ISL_7715369, EPI_ISL_7715370, EPI_ISL_7715371, EPI_ISL_7715372, EPI_ISL_7715373, EPI_ISL_7715374, EPI_ISL_7715375, EPI_ISL_7715376, EPI_ISL_7715377, EPI_ISL_7715378, EPI_ISL_7715379, EPI_ISL_7715380, EPI_ISL_7715381, EPI_ISL_7715382, EPI_ISL_7715383, EPI_ISL_7715384, EPI_ISL_7715385, EPI_ISL_7715386, EPI_ISL_7715387, EPI_ISL_7715388, EPI_ISL_7715389, EPI_ISL_7715390, EPI_ISL_7715391, EPI_ISL_7715392, EPI_ISL_7715393, EPI_ISL_7715394, EPI_ISL_7715395, EPI_ISL_7715396, EPI_ISL_7715397, EPI_ISL_7715398, EPI_ISL_7715399, EPI_ISL_7715400, EPI_ISL_7715401, EPI_ISL_7715402, EPI_ISL_7747503, EPI_ISL_7747504			
see above	National Health Laboratory Services, Virology	CERI, Centre for Epidemic Response and Innvoation, Stellenbosch University and KRISP, KZN Research Innovation and Sequencing Platform, UKZN.	Arisha Maharaj; Avani Bharuthram; Florette Treurnicht; Giandhari J; Kathleen Subramoney; Naidoo Y; Nkhensani Mtileni; Pillay S; Ramphal U; Ramphal Y; San JE; Tegally H; Tshiabuila D; Wilkinson E; de Oliveira T
EPI_ISL_7852833, EPI_ISL_7852834, EPI_ISL_7852835, EPI_ISL_7852836, EPI_ISL_7852837, EPI_ISL_7852838, EPI_ISL_7852839, EPI_ISL_7852840, EPI_ISL_7852841, EPI_ISL_7852842, EPI_ISL_7852843, EPI_ISL_7852844, EPI_ISL_7852845, EPI_ISL_7852846, EPI_ISL_7852847, EPI_ISL_7852848, EPI_ISL_7852849, EPI_ISL_7852850, EPI_ISL_7852851, EPI_ISL_7852852, EPI_ISL_7852853, EPI_ISL_7852854, EPI_ISL_7852855, EPI_ISL_7852856, EPI_ISL_7852857, EPI_ISL_7852858, EPI_ISL_7852859, EPI_ISL_7852860, EPI_ISL_7852861, EPI_ISL_7852862, EPI_ISL_7852863, EPI_ISL_7852864, EPI_ISL_7852865, EPI_ISL_7852866, EPI_ISL_7852867, EPI_ISL_7852868, EPI_ISL_7852869, EPI_ISL_7852870, EPI_ISL_7852871, EPI_ISL_7852872, EPI_ISL_7852873, EPI_ISL_7852874, EPI_ISL_7852875, EPI_ISL_7852876, EPI_ISL_7852877, EPI_ISL_7852878, EPI_ISL_7852879, EPI_ISL_7852880, EPI_ISL_7852881, EPI_ISL_7852882, EPI_ISL_7852883, EPI_ISL_7852884, EPI_ISL_7852885, EPI_ISL_7852886, EPI_ISL_7852887, EPI_ISL_7852888, EPI_ISL_7852889, EPI_ISL_7852890, EPI_ISL_7852891			
see above	National Health Laboratory Services, Virology	National Health Laboratory Services, Virology	Avani Bharuthram; Florette Treurnicht; Kathleen Subramoney; Nkhensani Mtileni
EPI_ISL_6699728, EPI_ISL_6699729, EPI_ISL_6699730, EPI_ISL_6699731, EPI_ISL_6699732, EPI_ISL_6699733, EPI_ISL_6699734, EPI_ISL_6699735, EPI_ISL_6699736, EPI_ISL_6699737, EPI_ISL_6699738, EPI_ISL_6699739, EPI_ISL_6699740, EPI_ISL_6699741, EPI_ISL_6699742, EPI_ISL_6699743, EPI_ISL_6699744, EPI_ISL_6699745, EPI_ISL_6699746, EPI_ISL_6699747, EPI_ISL_6699748, EPI_ISL_6699749, EPI_ISL_6699750, EPI_ISL_6699751, EPI_ISL_6699752, EPI_ISL_6699753, EPI_ISL_6699754, EPI_ISL_6699755, EPI_ISL_6699756, EPI_ISL_6699757, EPI_ISL_6699758, EPI_ISL_6699759, EPI_ISL_6699760, EPI_ISL_6699761, EPI_ISL_6699762, EPI_ISL_6699763, EPI_ISL_6699764, EPI_ISL_6699765, EPI_ISL_6699766, EPI_ISL_6699767, EPI_ISL_6699768, EPI_ISL_6699769, EPI_ISL_6699770, EPI_ISL_6699771, EPI_ISL_6782043, EPI_ISL_6782048, EPI_ISL_6782055, EPI_ISL_6782056, EPI_ISL_6782066, EPI_ISL_6782071, EPI_ISL_6782079, EPI_ISL_6782080, EPI_ISL_6782084, EPI_ISL_6782090, EPI_ISL_6782091, EPI_ISL_6782092, EPI_ISL_6810481, EPI_ISL_6810482, EPI_ISL_6810483, EPI_ISL_6810484, EPI_ISL_6810485, EPI_ISL_6810486, EPI_ISL_6810487, EPI_ISL_7015157, EPI_ISL_7015158, EPI_ISL_7015159, EPI_ISL_7015160, EPI_ISL_7015161, EPI_ISL_7015162, EPI_ISL_7015163, EPI_ISL_7015164, EPI_ISL_7015165, EPI_ISL_7015166, EPI_ISL_7015167, EPI_ISL_7015168, EPI_ISL_7015169, EPI_ISL_7015170, EPI_ISL_7417114, EPI_ISL_7417129, EPI_ISL_7417159, EPI_ISL_7417169, EPI_ISL_7417267, EPI_ISL_7417285, EPI_ISL_7417376, EPI_ISL_7417383, EPI_ISL_7417384			
see above	National Health Laboratory Services, Virology, Charlotte Maxeke Johannesburg hospital, Parktown, Johannesburg, Gauteng	CERI, Centre for Epidemic Response and Innvoation, Stellenbosch University and KRISP, KZN Research Innovation and Sequencing Platform, UKZN.	Amoaka D; Arisha Maharaj; Avani Bharuthram; Bester P; Bhiman J; Engelbrecht S; Everatt J; Florette Treurnicht; Giandhari J; Goedhals D; Hardie D; Hsiao M; Iranzadeh A; Kathleen Subramoney; Lessells R; Makatini Z; Maponga T; Mdlalose N; Mlisana K; Moir M; NGS-SA (Scheepers C; Naidoo Y; Nkhensani Mtileni; Nyaga M) Giandhari J; Oluwakemi M; Pillay S; Preiser W; Ramphal U; Ramphal Y; San JE; Tegally H; Tshiabuila D; Venter M; Wilkinson E; Williamson C; de Oliveira T; von Gottberg A
EPI_ISL_4891489, EPI_ISL_4891510, EPI_ISL_4891522, EPI_ISL_4891528, EPI_ISL_4891579, EPI_ISL_4891586, EPI_ISL_4891588, EPI_ISL_4891595, EPI_ISL_4891610, EPI_ISL_4891613, EPI_ISL_4891639, EPI_ISL_4891645, EPI_ISL_4891656, EPI_ISL_4891661, EPI_ISL_4891667, EPI_ISL_4891844, EPI_ISL_4891850, EPI_ISL_4891851, EPI_ISL_4891852, EPI_ISL_5917845, EPI_ISL_5917864, EPI_ISL_5917908, EPI_ISL_5918045, EPI_ISL_5918057, EPI_ISL_5918182, EPI_ISL_5918183, EPI_ISL_5918184, EPI_ISL_5919750, EPI_ISL_7456447, EPI_ISL_7456448, EPI_ISL_7456452, EPI_ISL_7456455, EPI_ISL_7456456, EPI_ISL_7456457			
see above	National Institute For Communicable Diseases Of The National Health Laboratory Service	National Institute for Communicable Diseases of the National Health Laboratory Service	Amoako DG; Bhiman JN; Everatt J; Ismail A; Mahlangu B; Mnguni A; Mohale T; Ntuli N; Scheepers C; Wolter N
EPI_ISL_2444091, EPI_ISL_2662708, EPI_ISL_2662709, EPI_ISL_2662710, EPI_ISL_2662711, EPI_ISL_2662712, EPI_ISL_2662713, EPI_ISL_2662714, EPI_ISL_2662715, EPI_ISL_2662716, EPI_ISL_2662717, EPI_ISL_2662718, EPI_ISL_2662719, EPI_ISL_2662720, EPI_ISL_2662721, EPI_ISL_2662722, EPI_ISL_2662723, EPI_ISL_2662724, EPI_ISL_2662725, EPI_ISL_2984801, EPI_ISL_3101635, EPI_ISL_3101636, EPI_ISL_3101637, EPI_ISL_3101638, EPI_ISL_3101656, EPI_ISL_3101657, EPI_ISL_3101658, EPI_ISL_3101659, EPI_ISL_3101660, EPI_ISL_3101661, EPI_ISL_3101662, EPI_ISL_3101663, EPI_ISL_3101664, EPI_ISL_3149306, EPI_ISL_3219783, EPI_ISL_3219784, EPI_ISL_3219785, EPI_ISL_3219786, EPI_ISL_3219787, EPI_ISL_3219788, EPI_ISL_3219789, EPI_ISL_3219790, EPI_ISL_3219791, EPI_ISL_3219792, EPI_ISL_3219793, EPI_ISL_3219794, EPI_ISL_3219795, EPI_ISL_3219796, EPI_ISL_3219797, EPI_ISL_3219798, EPI_ISL_3219799, EPI_ISL_3219800, EPI_ISL_3219801, EPI_ISL_3219802, EPI_ISL_3219803, EPI_ISL_3219804, EPI_ISL_3219805, EPI_ISL_3219806, EPI_ISL_3219807, EPI_ISL_3219808, EPI_ISL_3219809, EPI_ISL_3219810, EPI_ISL_3219811, EPI_ISL_3219812, EPI_ISL_3219813, EPI_ISL_3219814, EPI_ISL_3219815, EPI_ISL_3219816, EPI_ISL_3219817, EPI_ISL_3219818, EPI_ISL_3219819, EPI_ISL_3219820, EPI_ISL_3219821, EPI_ISL_3219822, EPI_ISL_3219823, EPI_ISL_3219824, EPI_ISL_3219825, EPI_ISL_3219826, EPI_ISL_3219827, EPI_ISL_3219828, EPI_ISL_3219829, EPI_ISL_3219830, EPI_ISL_3219831, EPI_ISL_3219832, EPI_ISL_3219833, EPI_ISL_3219835, EPI_ISL_3219836, EPI_ISL_3219837, EPI_ISL_3219838, EPI_ISL_3219839, EPI_ISL_3219840, EPI_ISL_3219841, EPI_ISL_3219842, EPI_ISL_3219843, EPI_ISL_3219844, EPI_ISL_3219845, EPI_ISL_3219846, EPI_ISL_3219847, EPI_ISL_3219848, EPI_ISL_3219849, EPI_ISL_3219850, EPI_ISL_3219851, EPI_ISL_3219852, EPI_ISL_3219853, EPI_ISL_3219854, EPI_ISL_3219855, EPI_ISL_3219856, EPI_ISL_3219857, EPI_ISL_3219858, EPI_ISL_3219859, EPI_ISL_3219860, EPI_ISL_3219861, EPI_ISL_3219862, EPI_ISL_3219863, EPI_ISL_3219864, EPI_ISL_3219865, EPI_ISL_3219878, EPI_ISL_3219879, EPI_ISL_3219880, EPI_ISL_3219881, EPI_ISL_3219882, EPI_ISL_3219883, EPI_ISL_3219884, EPI_ISL_3219885, EPI_ISL_3219886, EPI_ISL_3281585, EPI_ISL_3281586, EPI_ISL_3281588, EPI_ISL_3281589, EPI_ISL_3281590, EPI_ISL_3281591, EPI_ISL_3281592, EPI_ISL_3281593, EPI_ISL_3281594, EPI_ISL_3281595, EPI_ISL_3281596, EPI_ISL_3281597, EPI_ISL_3281598, EPI_ISL_3281599, EPI_ISL_3281600, EPI_ISL_3281601, EPI_ISL_3281602, EPI_ISL_3342435, EPI_ISL_3342436, EPI_ISL_3342437, EPI_ISL_3342438, EPI_ISL_3342439, EPI_ISL_3342440, EPI_ISL_3342441, EPI_ISL_3411817, EPI_ISL_3451570, EPI_ISL_3451571, EPI_ISL_3451572, EPI_ISL_3451573, EPI_ISL_3451574, EPI_ISL_3451575, EPI_ISL_3451576, EPI_ISL_3451577, EPI_ISL_3451593, EPI_ISL_3451634, EPI_ISL_3451636, EPI_ISL_3451637, EPI_ISL_3643852, EPI_ISL_3643957, EPI_ISL_3644044, EPI_ISL_3644066, EPI_ISL_3717965, EPI_ISL_3717994, EPI_ISL_3718045, EPI_ISL_3718046, EPI_ISL_3718047, EPI_ISL_3718066, EPI_ISL_4029915, EPI_ISL_4029941, EPI_ISL_4029956, EPI_ISL_4029973, EPI_ISL_4030023, EPI_ISL_4030024, EPI_ISL_4030069, EPI_ISL_4030084, EPI_ISL_4030100, EPI_ISL_4253671, EPI_ISL_4651683, EPI_ISL_4651747, EPI_ISL_4651872, EPI_ISL_4652276, EPI_ISL_5477158, EPI_ISL_5477595, EPI_ISL_5477673, EPI_ISL_6204411, EPI_ISL_6886555, EPI_ISL_6886558, EPI_ISL_7605585, EPI_ISL_7605586, EPI_ISL_7605593, EPI_ISL_7605594, EPI_ISL_7605629, EPI_ISL_7605630, EPI_ISL_7605631, EPI_ISL_7605632, EPI_ISL_7605637, EPI_ISL_7605638, EPI_ISL_7605666, EPI_ISL_7605720, EPI_ISL_7605759, EPI_ISL_7605762, EPI_ISL_7605765, EPI_ISL_7605767, EPI_ISL_7605768, EPI_ISL_7605770, EPI_ISL_7605771, EPI_ISL_8002879, EPI_ISL_8002880, EPI_ISL_8002883, EPI_ISL_8002884, EPI_ISL_8002885, EPI_ISL_8002886, EPI_ISL_8002889, EPI_ISL_8002890, EPI_ISL_8002891, EPI_ISL_8002893, EPI_ISL_8002894, EPI_ISL_8002895, EPI_ISL_8002896, EPI_ISL_8002899, EPI_ISL_8002902, EPI_ISL_8002903, EPI_ISL_8002904, EPI_ISL_8002905, EPI_ISL_8002907, EPI_ISL_8002908, EPI_ISL_8002909, EPI_ISL_8002910, EPI_ISL_8002911, EPI_ISL_8002912, EPI_ISL_8051873, EPI_ISL_8051874, EPI_ISL_8051875, EPI_ISL_8051876, EPI_ISL_8051877, EPI_ISL_8051882, EPI_ISL_8051883, EPI_ISL_8051884, EPI_ISL_8051885			
see above	National Institute for Communicable Diseases of the National Health Laboratory Service	National Institute for Communicable Diseases of the National Health Laboratory Service	Amoako DG; Bhiman JN; Everatt J; Ismail A; Kekana D; Mahlangu B; Mnguni A; Mohale T; Ntuli N; Scheepers C; Sisonke Team; Wolter N
EPI_ISL_939656, EPI_ISL_939657, EPI_ISL_939658, EPI_ISL_939659, EPI_ISL_940067, EPI_ISL_1239850, EPI_ISL_1239882, EPI_ISL_1827555, EPI_ISL_1827556, EPI_ISL_1827557, EPI_ISL_1827558, EPI_ISL_1827559, EPI_ISL_1827560, EPI_ISL_1827561, EPI_ISL_1827562, EPI_ISL_1827563, EPI_ISL_1827564, EPI_ISL_1827565, EPI_ISL_1827566, EPI_ISL_1827567, EPI_ISL_1827568, EPI_ISL_1827569, EPI_ISL_1827570, EPI_ISL_1827571, EPI_ISL_1827572, EPI_ISL_1827573, EPI_ISL_1827574, EPI_ISL_1827575, EPI_ISL_1827576, EPI_ISL_1827577, EPI_ISL_1827578, EPI_ISL_1827579, EPI_ISL_1827580, EPI_ISL_1827581, EPI_ISL_1827582, EPI_ISL_1827583, EPI_ISL_1827584, EPI_ISL_1827585, EPI_ISL_1827586, EPI_ISL_1827587, EPI_ISL_1827588, EPI_ISL_1827589, EPI_ISL_1827590, EPI_ISL_1827591, EPI_ISL_1827592, EPI_ISL_1827593, EPI_ISL_1827594, EPI_ISL_1827595, EPI_ISL_1827596, EPI_ISL_1827597, EPI_ISL_1827598, EPI_ISL_1827599, EPI_ISL_1827600, EPI_ISL_1827601, EPI_ISL_1827602, EPI_ISL_1827603, EPI_ISL_1827604, EPI_ISL_1827605, EPI_ISL_1827606, EPI_ISL_1827607, EPI_ISL_1827608, EPI_ISL_1827609, EPI_ISL_1827610, EPI_ISL_1827611, EPI_ISL_1827612, EPI_ISL_1827613, EPI_ISL_1827614, EPI_ISL_1827615, EPI_ISL_1827616, EPI_ISL_1827618, EPI_ISL_1827620, EPI_ISL_1827621, EPI_ISL_1827622, EPI_ISL_1827623, EPI_ISL_1827624, EPI_ISL_1827625, EPI_ISL_1827626, EPI_ISL_1827627, EPI_ISL_1827628, EPI_ISL_1827629, EPI_ISL_1827630, EPI_ISL_1827631, EPI_ISL_1827632, EPI_ISL_1827633, EPI_ISL_1914786, EPI_ISL_1914787, EPI_ISL_1914788, EPI_ISL_1914789, EPI_ISL_1914790, EPI_ISL_1914791, EPI_ISL_1914792, EPI_ISL_1914793, EPI_ISL_1914794, EPI_ISL_1914795, EPI_ISL_1914796, EPI_ISL_1914797, EPI_ISL_1914798, EPI_ISL_1914799, EPI_ISL_1914800, EPI_ISL_1914801, EPI_ISL_1914802, EPI_ISL_1914803, EPI_ISL_1914804, EPI_ISL_1914805, EPI_ISL_1914806, EPI_ISL_1914807, EPI_ISL_1914808, EPI_ISL_1914809, EPI_ISL_1914810, EPI_ISL_1914811, EPI_ISL_1914812, EPI_ISL_1914813, EPI_ISL_1914814, EPI_ISL_1914815, EPI_ISL_1914816, EPI_ISL_1914817, EPI_ISL_1914818, EPI_ISL_1914819, EPI_ISL_1914820, EPI_ISL_1914821, EPI_ISL_1914822, EPI_ISL_1914823, EPI_ISL_1914824, EPI_ISL_1914825, EPI_ISL_1914826, EPI_ISL_1914827, EPI_ISL_1914828, EPI_ISL_1914829, EPI_ISL_1914830, EPI_ISL_1914831, EPI_ISL_1914832, EPI_ISL_1914833, EPI_ISL_1914834, EPI_ISL_1914835, EPI_ISL_1914836, EPI_ISL_1914837, EPI_ISL_1914838, EPI_ISL_1914839, EPI_ISL_1914840, EPI_ISL_1914841, EPI_ISL_1914842, EPI_ISL_1914843, EPI_ISL_1914845, EPI_ISL_1914848, EPI_ISL_1914849, EPI_ISL_1914850, EPI_ISL_1914851, EPI_ISL_1914852, EPI_ISL_1914853, EPI_ISL_1914854, EPI_ISL_1914855, EPI_ISL_1914856, EPI_ISL_1914857, EPI_ISL_1914858, EPI_ISL_1914859, EPI_ISL_1914860, EPI_ISL_1914861, EPI_ISL_1914862, EPI_ISL_1914863, EPI_ISL_1914864, EPI_ISL_1914865, EPI_ISL_1914866, EPI_ISL_1914867, EPI_ISL_1914868, EPI_ISL_1914869, EPI_ISL_1914870, EPI_ISL_1914871, EPI_ISL_1914872, EPI_ISL_1914873, EPI_ISL_1914874, EPI_ISL_1914875, EPI_ISL_1914876, EPI_ISL_1914877, EPI_ISL_1914878, EPI_ISL_1914879, EPI_ISL_1914880, EPI_ISL_1914881, EPI_ISL_1914882, EPI_ISL_1914883, EPI_ISL_1914884, EPI_ISL_1914885, EPI_ISL_1914887, EPI_ISL_1914888, EPI_ISL_1914889, EPI_ISL_1914890, EPI_ISL_1914891, EPI_ISL_1914892, EPI_ISL_1914893, EPI_ISL_1914894, EPI_ISL_1914895, EPI_ISL_1914896, EPI_ISL_1914897, EPI_ISL_1914898, EPI_ISL_1914899, EPI_ISL_1914900, EPI_ISL_1914901, EPI_ISL_1914902, EPI_ISL_1914903, EPI_ISL_1914904, EPI_ISL_1914905, EPI_ISL_1914906, EPI_ISL_1914907, EPI_ISL_1914908, EPI_ISL_1914909, EPI_ISL_1914910, EPI_ISL_1914911, EPI_ISL_1914912, EPI_ISL_1914913, EPI_ISL_1914914, EPI_ISL_1914915, EPI_ISL_1914916, EPI_ISL_1914917, EPI_ISL_1914918, EPI_ISL_1914919, EPI_ISL_1914920, EPI_ISL_1914921, EPI_ISL_1914922, EPI_ISL_1914923, EPI_ISL_1914924, EPI_ISL_1914925, EPI_ISL_1914926, EPI_ISL_1914927, EPI_ISL_1914928, EPI_ISL_1914929, EPI_ISL_1914930, EPI_ISL_1914932, EPI_ISL_1914933, EPI_ISL_1914934, EPI_ISL_1914935			
see above	National Institute for Communicable Diseases,National Health Laboratory Services, Gauteng, South Africa	National Institute for Communicable Diseases of the National Health Laboratory Service	Allam M; Amoako DG; Bhiman JN; Ismail A; Mahlangu B; Mohale T; Ntuli N; Scheepers C
EPI_ISL_4575097, EPI_ISL_4575118, EPI_ISL_4575178, EPI_ISL_4575179	Netcare Linksfield	KRISP, KZN Research Innovation and Sequencing Platform	Emmanuel SJ; Giandhari J; Lessells R; Naidoo Y; Pillay S; Ramphal U; Tegally H; Wilkinson E; de Oliveira T
EPI_ISL_2086211, EPI_ISL_2086212, EPI_ISL_2086213, EPI_ISL_2086214, EPI_ISL_2086215, EPI_ISL_2086216, EPI_ISL_2086217, EPI_ISL_2086218, EPI_ISL_2086219			
see above	Netcare Linksfield Hospital	KRISP, KZN Research Innovation and Sequencing Platform	Emmanuel S; Emmanuel SJ; Giandhari J; Lessells R; Naidoo Y; Pillay S; Ramphal U; Tegally H; Wilkinson E; de Oliveira T
EPI_ISL_4575055, EPI_ISL_4575199	Netcare Milpark	KRISP, KZN Research Innovation and Sequencing Platform	Emmanuel SJ; Giandhari J; Lessells R; Naidoo Y; Pillay S; Ramphal U; Tegally H; Wilkinson E; de Oliveira T
EPI_ISL_4575056	Netcare Olivedale Hospital	KRISP, KZN Research Innovation and Sequencing Platform	Emmanuel SJ; Giandhari J; Lessells R; Naidoo Y; Pillay S; Ramphal U; Tegally H; Wilkinson E; de Oliveira T
EPI_ISL_5510409	ORT Mobile lab	National Institute for Communicable Diseases of the National Health Laboratory Service	Amoako DG; Bhiman JN; Everatt J; Ismail A; Mahlangu B; Mnguni A; Mohale T; Ntuli N; Scheepers C
EPI_ISL_2086977, EPI_ISL_2086978	PATH CARE	National Institute for Communicable Diseases of the National Health Laboratory Service	Amoako DG; Bhiman JN; Ismail A; Mahlangu B; Mohale T; Ntuli N; Scheepers C
EPI_ISL_3838570, EPI_ISL_3838571, EPI_ISL_4651723, EPI_ISL_4651764, EPI_ISL_4651842, EPI_ISL_4652073, EPI_ISL_4652083, EPI_ISL_6327912			
see above	PATHCARE	National Institute for Communicable Diseases of the National Health Laboratory Service	Amoako DG; Bhiman JN; Everatt J; Ismail A; Mahlangu B; Mnguni A; Mohale T; Ntuli N; Scheepers C
EPI_ISL_2695583, EPI_ISL_2695584, EPI_ISL_2695585, EPI_ISL_2695586, EPI_ISL_2695587	PATHCARE LABORATORY	National Institute for Communicable Diseases of the National Health Laboratory Service	Amoako DG; Bhiman JN; Everatt J; Ismail A; Mahlangu B; Mnguni A; Mohale T; Ntuli N; Scheepers C

EPI_ISL_8051886, EPI_ISL_8051887, EPI_ISL_8051888	PATHCARe LABORATORY	National Institute for Communicable Diseases of the National Health Laboratory Service	Amoako DG; Bhiman JN; Everatt J; Ismail A; Kekana D; Mahlangu B; Mnguni A; Mohale T; Ntuli N; Scheepers C; Wolter N
EPI_ISL_2709978	PHOLOSONG LABORATORY	National Institute for Communicable Diseases of the National Health Laboratory Service	Amoako DG; Bhiman JN; Everatt J; Ismail A; Mahlangu B; Mnguni A; Mohale T; Ntuli N; Scheepers C
EPI_ISL_3237004, EPI_ISL_3237005, EPI_ISL_3237006, EPI_ISL_3237007, EPI_ISL_3237008, EPI_ISL_3237009, EPI_ISL_3237010, EPI_ISL_3237011, EPI_ISL_3237012, EPI_ISL_3237013, EPI_ISL_3237016, EPI_ISL_3237017, EPI_ISL_3237018, EPI_ISL_3237019, EPI_ISL_3237020, EPI_ISL_3237197, EPI_ISL_3237198, EPI_ISL_3237199, EPI_ISL_3237200, EPI_ISL_3237201, EPI_ISL_3237202, EPI_ISL_3237203, EPI_ISL_3237204, EPI_ISL_3237205, EPI_ISL_3237206, EPI_ISL_3237207, EPI_ISL_3237208, EPI_ISL_3237218, EPI_ISL_3237219, EPI_ISL_3237220, EPI_ISL_3237221			
see above	PORT ELIZABETH	National Institute for Communicable Diseases of the National Health Laboratory Service	Amoako DG; Bhiman JN; Everatt J; Ismail A; Mahlangu B; Mnguni A; Mohale T; Ntuli N; Scheepers C
EPI_ISL_3411695	PORT ELIZABETH LABORATORY	National Institute for Communicable Diseases of the National Health Laboratory Service	Amoako DG; Bhiman JN; Everatt J; Ismail A; Mahlangu B; Mnguni A; Mohale T; Ntuli N; Scheepers C
EPI_ISL_3717896, EPI_ISL_3717898, EPI_ISL_3717903, EPI_ISL_3717907, EPI_ISL_3717909, EPI_ISL_3717911, EPI_ISL_3717920, EPI_ISL_3717932, EPI_ISL_3717935, EPI_ISL_3717942, EPI_ISL_3717946, EPI_ISL_3717948, EPI_ISL_3717972, EPI_ISL_3717975, EPI_ISL_3717979, EPI_ISL_3717980, EPI_ISL_3717982, EPI_ISL_3717993, EPI_ISL_3717999, EPI_ISL_3718000, EPI_ISL_3718038, EPI_ISL_3718039, EPI_ISL_3718040, EPI_ISL_3718041, EPI_ISL_3718042, EPI_ISL_3718043, EPI_ISL_3718044, EPI_ISL_5510391, EPI_ISL_5917989, EPI_ISL_5918035			
see above	Pathcare	National Institute for Communicable Diseases of the National Health Laboratory Service	Amoako DG; Bhiman JN; Everatt J; Ismail A; Mahlangu B; Mnguni A; Mohale T; Ntuli N; Scheepers C; Sisonke Team
EPI_ISL_2828734, EPI_ISL_2828735, EPI_ISL_2828736, EPI_ISL_2828737, EPI_ISL_2828738, EPI_ISL_2828739, EPI_ISL_2828740, EPI_ISL_2828741, EPI_ISL_2828742, EPI_ISL_2828743, EPI_ISL_2828744, EPI_ISL_2828745, EPI_ISL_2828746, EPI_ISL_2828747, EPI_ISL_2828748, EPI_ISL_2828749, EPI_ISL_2828750, EPI_ISL_2828751, EPI_ISL_2828752, EPI_ISL_2828753, EPI_ISL_2828754, EPI_ISL_2828755, EPI_ISL_2828756, EPI_ISL_2828757, EPI_ISL_2828758, EPI_ISL_3101583, EPI_ISL_3101584, EPI_ISL_3101585, EPI_ISL_3101586, EPI_ISL_3101587, EPI_ISL_3101588, EPI_ISL_3101589, EPI_ISL_3101590, EPI_ISL_3101591, EPI_ISL_3411556, EPI_ISL_3411557, EPI_ISL_3411558, EPI_ISL_3411559, EPI_ISL_3411560, EPI_ISL_3411561, EPI_ISL_3411562, EPI_ISL_3411563, EPI_ISL_3411564, EPI_ISL_3411565, EPI_ISL_3411566, EPI_ISL_3411567, EPI_ISL_3411568, EPI_ISL_3411569, EPI_ISL_3411570, EPI_ISL_3411576, EPI_ISL_3411577, EPI_ISL_3411578, EPI_ISL_3411579, EPI_ISL_3411581, EPI_ISL_3411582, EPI_ISL_3411583, EPI_ISL_3411681, EPI_ISL_5510334, EPI_ISL_5510349, EPI_ISL_5510486			
see above	Pathcare Vaal	National Institute for Communicable Diseases of the National Health Laboratory Service	Amoako DG; Bhiman JN; Everatt J; Ismail A; Mahlangu B; Mnguni A; Mohale T; Ntuli N; Scheepers C
EPI_ISL_2984940, EPI_ISL_2984941, EPI_ISL_2984942, EPI_ISL_2984943, EPI_ISL_2984944, EPI_ISL_2984945, EPI_ISL_2984946, EPI_ISL_2984947, EPI_ISL_2984948, EPI_ISL_2984949, EPI_ISL_2984950, EPI_ISL_2984951, EPI_ISL_2984952, EPI_ISL_2984953, EPI_ISL_2988410			
see above	Pathcare Vaal Laboratory	National Institute for Communicable Diseases of the National Health Laboratory Service	Amoako DG; Bhiman JN; Everatt J; Ismail A; Mahlangu B; Mnguni A; Mohale T; Ntuli N; Scheepers C
EPI_ISL_3236988, EPI_ISL_3237163, EPI_ISL_3237164, EPI_ISL_3237165, EPI_ISL_3237166	Pathcare Vaal laboratory	National Institute for Communicable Diseases of the National Health Laboratory Service	Amoako DG; Bhiman JN; Everatt J; Ismail A; Mahlangu B; Mnguni A; Mohale T; Ntuli N; Scheepers C
EPI_ISL_2013036, EPI_ISL_2013038, EPI_ISL_2013039, EPI_ISL_2013041, EPI_ISL_2013042	Pathcare-Vermaak Centurion	National Institute for Communicable Diseases of the National Health Laboratory Service	Amoako DG; Bhiman JN; Glass A; Gottberg A; Mahlangu B; Mohale T; Ntuli N; Oliveira TD; Scheepers C; Tegally H; Viana R
EPI_ISL_5510288, EPI_ISL_5510290, EPI_ISL_5510371, EPI_ISL_5510418, EPI_ISL_5510464, EPI_ISL_5510466, EPI_ISL_5510475, EPI_ISL_5510485			
see above	Pathcare/Vermaak Centurion	National Institute for Communicable Diseases of the National Health Laboratory Service	Amoako DG; Bhiman JN; Everatt J; Ismail A; Mahlangu B; Mnguni A; Mohale T; Ntuli N; Scheepers C
EPI_ISL_3838601, EPI_ISL_3838602, EPI_ISL_3838604, EPI_ISL_3838606, EPI_ISL_3838607, EPI_ISL_3838608, EPI_ISL_3838610, EPI_ISL_3838611, EPI_ISL_3838612, EPI_ISL_3838613, EPI_ISL_3838614, EPI_ISL_3838615, EPI_ISL_3838616, EPI_ISL_3838617, EPI_ISL_3838618, EPI_ISL_3838619, EPI_ISL_3838620, EPI_ISL_3838621, EPI_ISL_3838622, EPI_ISL_3838623, EPI_ISL_3838624, EPI_ISL_3838625, EPI_ISL_3838626, EPI_ISL_3838627, EPI_ISL_3838628, EPI_ISL_3838629, EPI_ISL_3838630, EPI_ISL_3838631, EPI_ISL_3838632, EPI_ISL_3838633, EPI_ISL_3838634, EPI_ISL_3838635, EPI_ISL_3838636, EPI_ISL_3838637, EPI_ISL_3838639, EPI_ISL_3838640, EPI_ISL_3838641, EPI_ISL_3838642, EPI_ISL_3838643, EPI_ISL_3838644, EPI_ISL_3838645, EPI_ISL_3838646, EPI_ISL_3838647, EPI_ISL_3838648, EPI_ISL_3838649, EPI_ISL_3838650, EPI_ISL_3838651, EPI_ISL_3838652, EPI_ISL_3838654, EPI_ISL_3838655, EPI_ISL_3838656, EPI_ISL_3838657, EPI_ISL_3838658, EPI_ISL_4253648, EPI_ISL_4253649, EPI_ISL_4253650, EPI_ISL_4253651, EPI_ISL_4253652, EPI_ISL_4253653, EPI_ISL_4253654, EPI_ISL_4253655, EPI_ISL_4253656, EPI_ISL_4253657, EPI_ISL_4253658, EPI_ISL_4253659, EPI_ISL_4253660, EPI_ISL_4253661, EPI_ISL_4253662, EPI_ISL_4253663, EPI_ISL_4253664, EPI_ISL_4253665, EPI_ISL_4253666, EPI_ISL_4253667, EPI_ISL_4253668, EPI_ISL_4253669, EPI_ISL_4253670, EPI_ISL_4651691, EPI_ISL_4651856, EPI_ISL_4651857, EPI_ISL_4651865, EPI_ISL_4652202, EPI_ISL_4652208, EPI_ISL_4652210, EPI_ISL_4652211, EPI_ISL_4652215, EPI_ISL_4652231, EPI_ISL_4652237, EPI_ISL_4652248, EPI_ISL_4652253, EPI_ISL_4652265, EPI_ISL_4652270			
see above	Private/Pathcare	National Institute for Communicable Diseases of the National Health Laboratory Service	Amoako DG; Bhiman JN; Everatt J; Ismail A; Mahlangu B; Mnguni A; Mohale T; Ntuli N; Scheepers C; Sisonke Team
EPI_ISL_4651817, EPI_ISL_4651980	ROB FERREIRA LABORATORY	National Institute for Communicable Diseases of the National Health Laboratory Service	Amoako DG; Bhiman JN; Everatt J; Ismail A; Mahlangu B; Mnguni A; Mohale T; Ntuli N; Scheepers C
EPI_ISL_3236982, EPI_ISL_3236983, EPI_ISL_3236984, EPI_ISL_3236985, EPI_ISL_3236993, EPI_ISL_3236994, EPI_ISL_3236995, EPI_ISL_3236996, EPI_ISL_3236997, EPI_ISL_3236998, EPI_ISL_3236999, EPI_ISL_3237001, EPI_ISL_3237002, EPI_ISL_3237003, EPI_ISL_3237150, EPI_ISL_3237151, EPI_ISL_3237152, EPI_ISL_3237153, EPI_ISL_3237173, EPI_ISL_3237174, EPI_ISL_3237175, EPI_ISL_3237176, EPI_ISL_3237177, EPI_ISL_3237178, EPI_ISL_3237179, EPI_ISL_3237180, EPI_ISL_3237181, EPI_ISL_3237182, EPI_ISL_3237183, EPI_ISL_3237184, EPI_ISL_3237185, EPI_ISL_3237186, EPI_ISL_3237187, EPI_ISL_3237188, EPI_ISL_3237189, EPI_ISL_3237190, EPI_ISL_3237191, EPI_ISL_3237192, EPI_ISL_3237193, EPI_ISL_3237194, EPI_ISL_3237195, EPI_ISL_3237196, EPI_ISL_5416476			
see above	RUSTENBURG LABORATORY	National Institute for Communicable Diseases of the National Health Laboratory Service	Amoako DG; Bhiman JN; Everatt J; Ismail A; Mahlangu B; Mnguni A; Mohale T; Ntuli N; Scheepers C
EPI_ISL_4891541	Rob Ferreira Laboratory	National Institute for Communicable Diseases of the National Health Laboratory Service	Amoako DG; Bhiman JN; Everatt J; Ismail A; Mahlangu B; Mnguni A; Mohale T; Ntuli N; Scheepers C
EPI_ISL_7545637, EPI_ISL_7545638, EPI_ISL_7545639, EPI_ISL_7545640, EPI_ISL_7545641, EPI_ISL_7545642, EPI_ISL_7545643, EPI_ISL_7545644, EPI_ISL_7545645, EPI_ISL_7545646, EPI_ISL_7545647, EPI_ISL_7545648, EPI_ISL_7545649, EPI_ISL_7545650, EPI_ISL_7545651			
see above	SAMRC	CERI, Centre for Epidemic Response and Innvoation, Stellenbosch University and KRISP, KZN Research Innovation and Sequencing Platform, UKZN.	Arisha Maharaj; Giandhari J; MRC; Naidoo Y; Pillay S; Ramphal U; Ramphal Y; San JE; Tegally H; Tshiabuila D; Wilkinson E; de Oliveira T
EPI_ISL_5510477	SOUTH RAND HOSPITAL	National Institute for Communicable Diseases of the National Health Laboratory Service	Amoako DG; Bhiman JN; Everatt J; Ismail A; Mahlangu B; Mnguni A; Mohale T; Ntuli N; Scheepers C
EPI_ISL_2086909, EPI_ISL_2086914, EPI_ISL_2086916, EPI_ISL_2086917, EPI_ISL_2086918, EPI_ISL_2086919, EPI_ISL_2086920, EPI_ISL_2086925, EPI_ISL_2086926, EPI_ISL_2086927, EPI_ISL_2086928, EPI_ISL_2086929, EPI_ISL_2086930			
see above	Sebokeng Lab	National Institute for Communicable Diseases of the National Health Laboratory Service	Amoako DG; Bhiman JN; Ismail A; Mahlangu B; Mohale T; Ntuli N; Scheepers C
EPI_ISL_3267722, EPI_ISL_3267723, EPI_ISL_3267724, EPI_ISL_3267726, EPI_ISL_3267727, EPI_ISL_3267728, EPI_ISL_3267729, EPI_ISL_3267730, EPI_ISL_3267731, EPI_ISL_3267732, EPI_ISL_3267733, EPI_ISL_3267734, EPI_ISL_3267735, EPI_ISL_3267737, EPI_ISL_3267738, EPI_ISL_3267739, EPI_ISL_3267742, EPI_ISL_3267743, EPI_ISL_3267744, EPI_ISL_3267745, EPI_ISL_3267746, EPI_ISL_3267747			
see above	Sisonke - BARC SA	CERI, Centre for Epidemic Response and Innovation, Stellenbosch Univeristy & KRISP, KZN Research Innovation and Sequencing Platform	Giandhari Jennifer; Naidoo Yeshnee; Pillay Sureshnee; San James; Tegally Houriiyah; Tshabuila Derek; Wilkinson Eduan; Yajna Ramphal; de Oliveira Tulio
EPI_ISL_2694611, EPI_ISL_2694612, EPI_ISL_2694613, EPI_ISL_2694616, EPI_ISL_2694617, EPI_ISL_2694618, EPI_ISL_2694619, EPI_ISL_2694620, EPI_ISL_2694621, EPI_ISL_2694622, EPI_ISL_2694623, EPI_ISL_2694624, EPI_ISL_2694625, EPI_ISL_2694626, EPI_ISL_2694632, EPI_ISL_2694678, EPI_ISL_2694679, EPI_ISL_2694680, EPI_ISL_2694681, EPI_ISL_2694682, EPI_ISL_2694683, EPI_ISL_2694684, EPI_ISL_2694685, EPI_ISL_2694686, EPI_ISL_2694687, EPI_ISL_2694688, EPI_ISL_2694689, EPI_ISL_2694690, EPI_ISL_2694691, EPI_ISL_2694692, EPI_ISL_2694693, EPI_ISL_2694694, EPI_ISL_2694695, EPI_ISL_2694696, EPI_ISL_2694697, EPI_ISL_2694698, EPI_ISL_2694699, EPI_ISL_2694700, EPI_ISL_2694701, EPI_ISL_2694702, EPI_ISL_2694703, EPI_ISL_2694704, EPI_ISL_2694705, EPI_ISL_2694706, EPI_ISL_2694707, EPI_ISL_2694708, EPI_ISL_2694709, EPI_ISL_2694710, EPI_ISL_2694711, EPI_ISL_2694712, EPI_ISL_2694713, EPI_ISL_2694714, EPI_ISL_2694715, EPI_ISL_2694716, EPI_ISL_2694717, EPI_ISL_2694718, EPI_ISL_2694719, EPI_ISL_2694720, EPI_ISL_2694721, EPI_ISL_2694722, EPI_ISL_2694723, EPI_ISL_2694724, EPI_ISL_2694725, EPI_ISL_2694726, EPI_ISL_2695504, EPI_ISL_2695505, EPI_ISL_2695507, EPI_ISL_2695508, EPI_ISL_2695509, EPI_ISL_2695510, EPI_ISL_2695511, EPI_ISL_2695514, EPI_ISL_2695515, EPI_ISL_2695516, EPI_ISL_2695517, EPI_ISL_2695518, EPI_ISL_2695519, EPI_ISL_2695520, EPI_ISL_2695521, EPI_ISL_2695522, EPI_ISL_2695525, EPI_ISL_2695526, EPI_ISL_2695527, EPI_ISL_2695528, EPI_ISL_2695529, EPI_ISL_3101535, EPI_ISL_3101537, EPI_ISL_3101538, EPI_ISL_3101539, EPI_ISL_3101540, EPI_ISL_3101541, EPI_ISL_3101542, EPI_ISL_3101543, EPI_ISL_3101544, EPI_ISL_3101545, EPI_ISL_3101546, EPI_ISL_3101547, EPI_ISL_3101548, EPI_ISL_3101549, EPI_ISL_3101550, EPI_ISL_3101551, EPI_ISL_3101552, EPI_ISL_3101553, EPI_ISL_3101554, EPI_ISL_3101555, EPI_ISL_3101556, EPI_ISL_3101557, EPI_ISL_3101558, EPI_ISL_3101559, EPI_ISL_3101560, EPI_ISL_3101561, EPI_ISL_3101562, EPI_ISL_3101563, EPI_ISL_3101564, EPI_ISL_3101565, EPI_ISL_3101566, EPI_ISL_3101567, EPI_ISL_3101568, EPI_ISL_3101569, EPI_ISL_3101570, EPI_ISL_5510280, EPI_ISL_5510282, EPI_ISL_5510301, EPI_ISL_5510305, EPI_ISL_5510341, EPI_ISL_5510421, EPI_ISL_5510474, EPI_ISL_5510481			
see above	TAMBO MEMORIAL	National Institute for Communicable Diseases of the National Health Laboratory Service	Amoako DG; Bhiman JN; Everatt J; Ismail A; Mahlangu B; Mnguni A; Mohale T; Ntuli N; Scheepers C; Sisonke Team
EPI_ISL_2153480, EPI_ISL_2153481, EPI_ISL_2153482, EPI_ISL_2662486, EPI_ISL_2662487, EPI_ISL_2662488, EPI_ISL_2662489, EPI_ISL_2662490, EPI_ISL_2662491, EPI_ISL_2662556, EPI_ISL_2662557, EPI_ISL_2662558, EPI_ISL_2662559, EPI_ISL_2662560, EPI_ISL_2662561, EPI_ISL_2662562, EPI_ISL_2662563, EPI_ISL_2662564, EPI_ISL_2662565, EPI_ISL_2662566, EPI_ISL_2662567, EPI_ISL_2662568, EPI_ISL_2662569, EPI_ISL_2662570, EPI_ISL_2662571, EPI_ISL_2662572, EPI_ISL_2662573, EPI_ISL_2662574, EPI_ISL_2662575, EPI_ISL_2662576, EPI_ISL_2662577, EPI_ISL_2662578, EPI_ISL_2662579, EPI_ISL_2662580, EPI_ISL_2662581, EPI_ISL_2662582, EPI_ISL_2662583, EPI_ISL_2662584, EPI_ISL_2662585, EPI_ISL_2662586, EPI_ISL_2662587, EPI_ISL_2662588, EPI_ISL_2662589, EPI_ISL_2662590, EPI_ISL_2662591, EPI_ISL_2662592, EPI_ISL_2662593, EPI_ISL_2662594, EPI_ISL_2662595, EPI_ISL_2662596, EPI_ISL_2662597, EPI_ISL_2662598, EPI_ISL_2662599, EPI_ISL_2662600, EPI_ISL_2662601, EPI_ISL_2662602, EPI_ISL_2662603, EPI_ISL_2662604, EPI_ISL_2662605, EPI_ISL_2662606, EPI_ISL_2662607, EPI_ISL_2662608, EPI_ISL_2662609, EPI_ISL_2662610, EPI_ISL_2662611, EPI_ISL_2662612, EPI_ISL_2662613, EPI_ISL_2662614, EPI_ISL_2662615, EPI_ISL_2662616, EPI_ISL_2662617, EPI_ISL_2695772, EPI_ISL_2695773, EPI_ISL_2695774, EPI_ISL_2695775, EPI_ISL_2695776, EPI_ISL_2695777, EPI_ISL_2695778, EPI_ISL_2695779, EPI_ISL_2695780, EPI_ISL_2695781, EPI_ISL_2695782, EPI_ISL_2695783, EPI_ISL_2695784, EPI_ISL_2709943, EPI_ISL_2709944, EPI_ISL_2709945, EPI_ISL_2709946, EPI_ISL_2709947, EPI_ISL_2709948, EPI_ISL_2709949, EPI_ISL_2709950, EPI_ISL_2709951, EPI_ISL_2709952, EPI_ISL_2709953, EPI_ISL_2709954, EPI_ISL_2709955, EPI_ISL_2709956, EPI_ISL_2709957, EPI_ISL_2709958, EPI_ISL_2709959, EPI_ISL_2709960, EPI_ISL_2709961, EPI_ISL_2709962, EPI_ISL_2709963, EPI_ISL_2709964, EPI_ISL_2709965, EPI_ISL_2709966, EPI_ISL_2709967, EPI_ISL_2709968, EPI_ISL_2709969, EPI_ISL_2709970, EPI_ISL_2709972, EPI_ISL_2709973, EPI_ISL_2709974, EPI_ISL_2709975, EPI_ISL_2709976, EPI_ISL_2709977, EPI_ISL_2709979, EPI_ISL_2709980, EPI_ISL_2709981, EPI_ISL_2709982, EPI_ISL_2709983, EPI_ISL_2709984, EPI_ISL_2709985, EPI_ISL_2709986, EPI_ISL_2709987, EPI_ISL_2709988, EPI_ISL_2709989, EPI_ISL_2709990, EPI_ISL_2709991, EPI_ISL_2709992, EPI_ISL_2709993, EPI_ISL_2709994, EPI_ISL_2827037, EPI_ISL_2827038, EPI_ISL_2827039, EPI_ISL_2827040, EPI_ISL_2827041, EPI_ISL_2827042, EPI_ISL_2827043, EPI_ISL_2827044, EPI_ISL_2827045, EPI_ISL_2827046, EPI_ISL_2827047, EPI_ISL_2827048, EPI_ISL_2827049, EPI_ISL_2827050, EPI_ISL_2827051, EPI_ISL_2827052, EPI_ISL_2827053, EPI_ISL_2827054, EPI_ISL_2827055, EPI_ISL_2827056, EPI_ISL_2827057, EPI_ISL_2827058, EPI_ISL_2827059, EPI_ISL_2827060, EPI_ISL_2827061, EPI_ISL_2827062, EPI_ISL_2827063, EPI_ISL_2827064, EPI_ISL_2827065, EPI_ISL_2827066, EPI_ISL_2827067, EPI_ISL_2827068, EPI_ISL_2827069, EPI_ISL_2827142, EPI_ISL_2827143, EPI_ISL_2827144, EPI_ISL_2827145, EPI_ISL_2827146, EPI_ISL_2827147, EPI_ISL_2827148, EPI_ISL_2827149, EPI_ISL_2827150, EPI_ISL_2827151, EPI_ISL_2827152, EPI_ISL_2827153, EPI_ISL_2827154, EPI_ISL_2827155, EPI_ISL_3024627, EPI_ISL_3024628, EPI_ISL_3024629, EPI_ISL_3024630, EPI_ISL_3024631, EPI_ISL_3024632, EPI_ISL_3024633, EPI_ISL_3024634, EPI_ISL_3024635, EPI_ISL_3024636, EPI_ISL_3024637, EPI_ISL_3024638, EPI_ISL_3024639, EPI_ISL_3024640, EPI_ISL_3024641, EPI_ISL_3024642, EPI_ISL_3074030, EPI_ISL_3074032, EPI_ISL_3074033, EPI_ISL_3074034, EPI_ISL_3074035, EPI_ISL_3074036, EPI_ISL_3074037, EPI_ISL_3074038, EPI_ISL_3074039, EPI_ISL_3074040, EPI_ISL_3074041, EPI_ISL_3236935, EPI_ISL_3236936, EPI_ISL_3236937, EPI_ISL_3236938, EPI_ISL_3236939, EPI_ISL_3236940, EPI_ISL_3236941, EPI_ISL_3236942, EPI_ISL_3236943, EPI_ISL_3236944, EPI_ISL_3236945, EPI_ISL_3236946, EPI_ISL_3236948, EPI_ISL_3236949, EPI_ISL_3236950, EPI_ISL_3236951, EPI_ISL_3236952, EPI_ISL_3236953, EPI_ISL_3236954, EPI_ISL_3237059, EPI_ISL_3237060, EPI_ISL_3237061, EPI_ISL_3237062, EPI_ISL_3237063, EPI_ISL_3237064, EPI_ISL_3237065, EPI_ISL_3237066, EPI_ISL_3237067, EPI_ISL_3237068, EPI_ISL_3237069, EPI_ISL_3237070, EPI_ISL_3237071, EPI_ISL_3237072, EPI_ISL_3237073, EPI_ISL_3237074, EPI_ISL_3237075, EPI_ISL_3237076, EPI_ISL_3237077, EPI_ISL_3237078, EPI_ISL_3237079, EPI_ISL_3237080, EPI_ISL_3237081, EPI_ISL_3237082, EPI_ISL_3237083, EPI_ISL_3237084, EPI_ISL_3237085, EPI_ISL_3237086, EPI_ISL_3237087, EPI_ISL_3237088, EPI_ISL_3237089, EPI_ISL_3237090, EPI_ISL_3237091, EPI_ISL_3237092, EPI_ISL_3237093, EPI_ISL_3237094, EPI_ISL_3237095, EPI_ISL_3237096, EPI_ISL_3237097, EPI_ISL_3237098, EPI_ISL_3237099, EPI_ISL_3237100, EPI_ISL_3237101, EPI_ISL_3237102, EPI_ISL_3237103, EPI_ISL_3342257, EPI_ISL_3342258, EPI_ISL_3342259, EPI_ISL_3342260, EPI_ISL_3342261, EPI_ISL_3342262, EPI_ISL_3342263, EPI_ISL_3342264, EPI_ISL_3342265, EPI_ISL_3411468, EPI_ISL_3451184, EPI_ISL_3451185, EPI_ISL_3451186, EPI_ISL_3451187, EPI_ISL_3451188, EPI_ISL_3451189, EPI_ISL_3451190, EPI_ISL_3451191, EPI_ISL_3451192, EPI_ISL_3451193, EPI_ISL_3451194, EPI_ISL_3451195, EPI_ISL_3451196, EPI_ISL_3451197, EPI_ISL_3451198, EPI_ISL_3451199, EPI_ISL_3451200, EPI_ISL_3451201, EPI_ISL_3451202, EPI_ISL_3451203, EPI_ISL_3451204, EPI_ISL_3451205, EPI_ISL_3451206, EPI_ISL_3451207, EPI_ISL_3451208, EPI_ISL_3451209, EPI_ISL_3451210, EPI_ISL_3451211, EPI_ISL_3451212, EPI_ISL_3451213, EPI_ISL_3451214, EPI_ISL_3451215, EPI_ISL_3451216, EPI_ISL_3451505, EPI_ISL_3451506, EPI_ISL_3451507, EPI_ISL_3451508, EPI_ISL_3451509, EPI_ISL_3451510, EPI_ISL_3451511, EPI_ISL_3451512, EPI_ISL_3451513, EPI_ISL_3451514, EPI_ISL_3451515, EPI_ISL_3451516, EPI_ISL_3451517, EPI_ISL_3451518, EPI_ISL_3451519, EPI_ISL_3451520, EPI_ISL_3451521, EPI_ISL_3451522, EPI_ISL_3451523, EPI_ISL_3451524, EPI_ISL_3451525, EPI_ISL_3451526, EPI_ISL_3451527, EPI_ISL_3451528, EPI_ISL_3451529, EPI_ISL_3717901, EPI_ISL_3717929, EPI_ISL_3717931, EPI_ISL_3717952, EPI_ISL_3717955, EPI_ISL_3717961, EPI_ISL_3717964, EPI_ISL_3717998, EPI_ISL_3718033, EPI_ISL_3718034, EPI_ISL_5196436, EPI_ISL_5196518, EPI_ISL_5196531, EPI_ISL_5196555, EPI_ISL_5196563, EPI_ISL_5196564, EPI_ISL_5196582, EPI_ISL_5196611, EPI_ISL_5196625, EPI_ISL_5416267, EPI_ISL_5416269, EPI_ISL_5416272, EPI_ISL_5416277, EPI_ISL_5416280, EPI_ISL_5416281, EPI_ISL_5416285, EPI_ISL_5416287, EPI_ISL_5416304, EPI_ISL_5416305, EPI_ISL_5416313, EPI_ISL_5416320, EPI_ISL_5416325, EPI_ISL_5416328, EPI_ISL_5416331, EPI_ISL_5416363, EPI_ISL_5416365, EPI_ISL_5416473, EPI_ISL_5416474, EPI_ISL_5416477, EPI_ISL_5416478, EPI_ISL_5416479, EPI_ISL_5416480, EPI_ISL_5416481, EPI_ISL_5416482, EPI_ISL_5416483, EPI_ISL_5416484, EPI_ISL_5416485, EPI_ISL_5416486, EPI_ISL_5416487, EPI_ISL_5416488, EPI_ISL_5416489, EPI_ISL_5416490, EPI_ISL_5416491, EPI_ISL_5416492, EPI_ISL_5416493, EPI_ISL_5416494, EPI_ISL_5416495, EPI_ISL_5416496, EPI_ISL_5416497, EPI_ISL_5416498, EPI_ISL_5416499, EPI_ISL_5416500, EPI_ISL_5416501, EPI_ISL_5416502, EPI_ISL_5416503, EPI_ISL_5416504, EPI_ISL_5416505, EPI_ISL_5416506, EPI_ISL_5416507, EPI_ISL_5603066, EPI_ISL_5603067, EPI_ISL_5603068, EPI_ISL_5603069, EPI_ISL_5603070, EPI_ISL_5603074, EPI_ISL_5603085, EPI_ISL_5603133, EPI_ISL_5603137, EPI_ISL_5603138, EPI_ISL_5603145, EPI_ISL_5603153, EPI_ISL_5603160, EPI_ISL_5603162, EPI_ISL_5603168, EPI_ISL_5603173, EPI_ISL_5603174, EPI_ISL_5603179, EPI_ISL_5603189, EPI_ISL_6201577, EPI_ISL_6202177, EPI_ISL_6202187, EPI_ISL_6202234, EPI_ISL_6202310, EPI_ISL_6202316, EPI_ISL_6202339, EPI_ISL_6202387, EPI_ISL_6202400, EPI_ISL_6202430, EPI_ISL_6202435, EPI_ISL_6202453, EPI_ISL_6202476, EPI_ISL_6202480, EPI_ISL_6202495, EPI_ISL_6202503, EPI_ISL_6202511, EPI_ISL_6202519, EPI_ISL_6202531, EPI_ISL_6202556, EPI_ISL_6202565, EPI_ISL_6202574, EPI_ISL_6202583, EPI_ISL_6202597, EPI_ISL_6202606, EPI_ISL_6202617, EPI_ISL_6202630, EPI_ISL_6202657, EPI_ISL_6202661, EPI_ISL_6202749, EPI_ISL_6202812, EPI_ISL_6203025, EPI_ISL_6203207, EPI_ISL_6203304, EPI_ISL_6203356, EPI_ISL_6203359, EPI_ISL_6203417, EPI_ISL_6203461, EPI_ISL_6203484, EPI_ISL_6203593, EPI_ISL_6203605, EPI_ISL_6900134, EPI_ISL_6900135, EPI_ISL_6906374, EPI_ISL_6906375, EPI_ISL_6906383			
see above	TAMBO MEMORIAL LABORATORY	National Institute for Communicable Diseases of the National Health Laboratory Service	Amoako DG; Bhiman JN; Everatt J; Ismail A; Mahlangu B; Mnguni A; Mohale T; Ntuli N; Scheepers C; Wolter N

EPI_ISL_5510487	THUTHUKANI CLINIC	National Institute for Communicable Diseases of the National Health Laboratory Service	Amoako DG; Bhiman JN; Everatt J; Ismail A; Mahlangu B; Mnguni A; Mohale T; Ntuli N; Scheepers C
EPI_ISL_3236977, EPI_ISL_3236978, EPI_ISL_3237116, EPI_ISL_3237135, EPI_ISL_3237136, EPI_ISL_3237137, EPI_ISL_3237138, EPI_ISL_5196441, EPI_ISL_5196442, EPI_ISL_5196528, EPI_ISL_7971571, EPI_ISL_7971580			
see above	TSHEPONG LABORATORY	National Institute for Communicable Diseases of the National Health Laboratory Service	Amoako DG; Bhiman JN; Everatt J; Ismail A; Kekana D; Mahlangu B; Mnguni A; Mohale T; Ntuli N; Scheepers C; Wolter N
EPI_ISL_5510389	TSHWANE ACADEMIC	National Institute for Communicable Diseases of the National Health Laboratory Service	Amoako DG; Bhiman JN; Everatt J; Ismail A; Mahlangu B; Mnguni A; Mohale T; Ntuli N; Scheepers C
EPI_ISL_2285301, EPI_ISL_2285302, EPI_ISL_2285304, EPI_ISL_2285305, EPI_ISL_2285306, EPI_ISL_2285307, EPI_ISL_2285308, EPI_ISL_2285309, EPI_ISL_2285310, EPI_ISL_2285311, EPI_ISL_2285312, EPI_ISL_2285313, EPI_ISL_3838566, EPI_ISL_3838567, EPI_ISL_3838568, EPI_ISL_4651617, EPI_ISL_4651628, EPI_ISL_4651636, EPI_ISL_4651647, EPI_ISL_4651651, EPI_ISL_4651659, EPI_ISL_4651677, EPI_ISL_4651704, EPI_ISL_4651724, EPI_ISL_4651726, EPI_ISL_4651752, EPI_ISL_4651755, EPI_ISL_4651787, EPI_ISL_4651810, EPI_ISL_4651898, EPI_ISL_4651906, EPI_ISL_4651919, EPI_ISL_4651984, EPI_ISL_4651996, EPI_ISL_4652005, EPI_ISL_4652015, EPI_ISL_4652027, EPI_ISL_4652041, EPI_ISL_4652046, EPI_ISL_4652055, EPI_ISL_4652062, EPI_ISL_4652065, EPI_ISL_4652284			
see above	TSHWANE ACADEMIC LABORATORY	National Institute for Communicable Diseases of the National Health Laboratory Service	Amoako DG; Bhiman JN; Everatt J; Ismail A; Mahlangu B; Mnguni A; Mohale T; Ntuli N; Scheepers C
EPI_ISL_3342730, EPI_ISL_3342731, EPI_ISL_3342732, EPI_ISL_3342733, EPI_ISL_5510313, EPI_ISL_5510314, EPI_ISL_5510362, EPI_ISL_5510374, EPI_ISL_5510386, EPI_ISL_5510439, EPI_ISL_5510455			
see above	Tambo Memorial	National Institute for Communicable Diseases of the National Health Laboratory Service	Amoako DG; Bhiman JN; Everatt J; Ismail A; Mahlangu B; Mnguni A; Mohale T; Ntuli N; Scheepers C
EPI_ISL_5917846, EPI_ISL_5917847, EPI_ISL_5917852, EPI_ISL_5917854, EPI_ISL_5917858, EPI_ISL_5917866, EPI_ISL_5917868, EPI_ISL_5917881, EPI_ISL_5917892, EPI_ISL_5917895, EPI_ISL_5917896, EPI_ISL_5917936, EPI_ISL_5917950, EPI_ISL_5917967, EPI_ISL_5917968, EPI_ISL_5917973, EPI_ISL_5917977, EPI_ISL_5917978, EPI_ISL_5917985, EPI_ISL_5918003, EPI_ISL_5918022, EPI_ISL_5918023, EPI_ISL_5918024, EPI_ISL_5918026, EPI_ISL_5918033, EPI_ISL_5918038, EPI_ISL_5918040, EPI_ISL_5918046, EPI_ISL_5918048, EPI_ISL_5918051, EPI_ISL_5918053, EPI_ISL_5918054, EPI_ISL_5918056, EPI_ISL_5918066, EPI_ISL_5918071, EPI_ISL_5918080, EPI_ISL_5918085, EPI_ISL_5918088, EPI_ISL_5918098, EPI_ISL_5918103, EPI_ISL_5918104, EPI_ISL_5918149, EPI_ISL_5918150, EPI_ISL_5918152, EPI_ISL_5918153, EPI_ISL_5919754, EPI_ISL_5919765, EPI_ISL_5919775, EPI_ISL_5919777, EPI_ISL_5919782, EPI_ISL_5919787, EPI_ISL_5919790, EPI_ISL_5919855, EPI_ISL_5919861, EPI_ISL_5919870, EPI_ISL_5919878, EPI_ISL_5919888, EPI_ISL_5919895, EPI_ISL_5919902, EPI_ISL_5919910, EPI_ISL_5919913, EPI_ISL_5919917, EPI_ISL_6913967, EPI_ISL_6913968, EPI_ISL_6913969, EPI_ISL_6913970, EPI_ISL_6913971, EPI_ISL_6913972, EPI_ISL_6913973, EPI_ISL_6913974, EPI_ISL_6913975, EPI_ISL_6913976, EPI_ISL_6913977, EPI_ISL_6913978, EPI_ISL_6913979, EPI_ISL_6913981, EPI_ISL_6913982, EPI_ISL_6913983, EPI_ISL_6913984, EPI_ISL_6913985, EPI_ISL_6913987, EPI_ISL_6913988, EPI_ISL_6913989, EPI_ISL_6913990, EPI_ISL_7337382, EPI_ISL_7337383, EPI_ISL_7337384, EPI_ISL_7337390, EPI_ISL_7337391, EPI_ISL_7337392, EPI_ISL_7337393, EPI_ISL_7337394, EPI_ISL_7337395, EPI_ISL_7337396, EPI_ISL_7337397, EPI_ISL_7337398, EPI_ISL_7337399, EPI_ISL_7337400, EPI_ISL_7337401, EPI_ISL_7337402, EPI_ISL_7337403, EPI_ISL_7337404, EPI_ISL_7337405, EPI_ISL_7337406, EPI_ISL_7337407, EPI_ISL_7337408, EPI_ISL_7337409, EPI_ISL_7337410, EPI_ISL_7337411, EPI_ISL_7337412, EPI_ISL_7337413, EPI_ISL_7337414, EPI_ISL_7337415, EPI_ISL_7337416, EPI_ISL_7456405, EPI_ISL_7456406, EPI_ISL_7456407, EPI_ISL_7456408, EPI_ISL_7456409, EPI_ISL_7456410, EPI_ISL_7456417, EPI_ISL_7456419, EPI_ISL_7456420, EPI_ISL_7456421, EPI_ISL_7456422, EPI_ISL_7456425, EPI_ISL_7456426, EPI_ISL_7456427, EPI_ISL_7456428, EPI_ISL_7456430, EPI_ISL_7456432, EPI_ISL_7456433, EPI_ISL_7456434, EPI_ISL_7456435, EPI_ISL_7456436, EPI_ISL_7456437, EPI_ISL_7456438, EPI_ISL_7456439, EPI_ISL_7456440, EPI_ISL_7456441, EPI_ISL_7456442, EPI_ISL_7456445, EPI_ISL_7456446			
see above	Tambo Memorial Laboratory	National Institute for Communicable Diseases of the National Health Laboratory Service	Amoako DG; Bhiman JN; Everatt J; Ismail A; Mahlangu B; Mnguni A; Mohale T; Ntuli N; Scheepers C; Wolter N
EPI_ISL_5510390	Tembisa	National Institute for Communicable Diseases of the National Health Laboratory Service	Amoako DG; Bhiman JN; Everatt J; Ismail A; Mahlangu B; Mnguni A; Mohale T; Ntuli N; Scheepers C
EPI_ISL_2271958, EPI_ISL_2271959, EPI_ISL_2271960, EPI_ISL_2271961, EPI_ISL_2271963, EPI_ISL_2271969, EPI_ISL_2271975, EPI_ISL_2271985, EPI_ISL_2272009, EPI_ISL_2272012			
see above	Tshepong Hospital	National Institute for Communicable Diseases of the National Health Laboratory Service	Amoako DG; Bhiman JN; Ismail A; Mahlangu B; Mohale T; Ntuli N; Scheepers C
EPI_ISL_5918113	Tshepong Laboratory	National Institute for Communicable Diseases of the National Health Laboratory Service	Amoako DG; Bhiman JN; Everatt J; Ismail A; Mahlangu B; Mnguni A; Mohale T; Ntuli N; Scheepers C
EPI_ISL_1324121, EPI_ISL_1324126	UW Virology Lab	UW Virology Lab	Alexander Greninger; Hong Xie; Keith R Jerome; Lasata Shrestha; Margaret Mills; Meei-Li Huang; Michelle Lin; Noah Baker; Pavitra Roychoudhury; Saraswathi Sathees; Sean Ellis; Shah Mohamed Bakhash
EPI_ISL_3237038, EPI_ISL_3237039, EPI_ISL_3237043, EPI_ISL_3237044, EPI_ISL_3237243, EPI_ISL_3237244, EPI_ISL_3237251, EPI_ISL_3237252, EPI_ISL_3237253, EPI_ISL_3411587, EPI_ISL_3411588, EPI_ISL_3411589, EPI_ISL_3411590, EPI_ISL_3411591, EPI_ISL_3411592, EPI_ISL_3411593, EPI_ISL_3411594, EPI_ISL_3411595, EPI_ISL_3411596, EPI_ISL_3411597, EPI_ISL_3411598, EPI_ISL_3411599, EPI_ISL_3411600, EPI_ISL_3411601, EPI_ISL_3411602, EPI_ISL_3411603, EPI_ISL_3411604, EPI_ISL_3411605, EPI_ISL_3411606, EPI_ISL_3411607, EPI_ISL_3411608, EPI_ISL_5510431			
see above	University Pretoria	National Institute for Communicable Diseases of the National Health Laboratory Service	Adriano Mendes; Amoako DG; Amy Strydom; Bhiman JN; Everatt J; Ismail A; Mahlangu B; Marietjie Venter; Micheala Davids; Mnguni A; Mohale T; Ntuli N; Scheepers C; Sim Mayaphi
EPI_ISL_6308308, EPI_ISL_6308309, EPI_ISL_6308310, EPI_ISL_6308311, EPI_ISL_6308315, EPI_ISL_6308316, EPI_ISL_6308320, EPI_ISL_6308327, EPI_ISL_6308336, EPI_ISL_6308340, EPI_ISL_6308341, EPI_ISL_6308351, EPI_ISL_6308356			
see above	University of Pretoria	CERI, Centre for Epidemic Response and Innvoation, Stellenbosch University and KRISP, KZN Research Innovation and Sequencing Platform, UKZN.	Adriano Mendes; Amy Strydom; Giandhari Jennifer; Marietjie Venter; Micheala Davids; Naidoo Yeshnee; Pillay Sureshnee; San James; Sim Mayaphi; Tegally Houriiyah; Tshiabuila Derek; Wilkinson Eduan; Yajna Ramphal; de Oliveira Tulio
EPI_ISL_2382656, EPI_ISL_2382657, EPI_ISL_2382658, EPI_ISL_2382659, EPI_ISL_2382660, EPI_ISL_2382661, EPI_ISL_2382662, EPI_ISL_2382664, EPI_ISL_2382665, EPI_ISL_2382666, EPI_ISL_2382667, EPI_ISL_2382668, EPI_ISL_2382670, EPI_ISL_2382671, EPI_ISL_2382673			
see above	VERMAAK	National Institute for Communicable Diseases of the National Health Laboratory Service	Amoako DG; Bhiman JN; Ismail A; Mahlangu B; Mohale T; Ntuli N; Scheepers C
EPI_ISL_2662476, EPI_ISL_2662477, EPI_ISL_2662478, EPI_ISL_2662479, EPI_ISL_2662480, EPI_ISL_2662482, EPI_ISL_2662483, EPI_ISL_2662484, EPI_ISL_2662485, EPI_ISL_2662530, EPI_ISL_2662537, EPI_ISL_7740759, EPI_ISL_7740763, EPI_ISL_7740766, EPI_ISL_7740854, EPI_ISL_7740857, EPI_ISL_7740858, EPI_ISL_7740859, EPI_ISL_7740860, EPI_ISL_7740861, EPI_ISL_7740866, EPI_ISL_7740885, EPI_ISL_7740889, EPI_ISL_7740890, EPI_ISL_7740897, EPI_ISL_7971522, EPI_ISL_7971523, EPI_ISL_7971524, EPI_ISL_7971525, EPI_ISL_7971526, EPI_ISL_7971527, EPI_ISL_7971528, EPI_ISL_7971529, EPI_ISL_7971530, EPI_ISL_7971531, EPI_ISL_7971532, EPI_ISL_7971533, EPI_ISL_7971534, EPI_ISL_7971535, EPI_ISL_7971536, EPI_ISL_7971537, EPI_ISL_7971538, EPI_ISL_7971539, EPI_ISL_7971540, EPI_ISL_7971541, EPI_ISL_7971542, EPI_ISL_7971543, EPI_ISL_7971544, EPI_ISL_7971545, EPI_ISL_7971546, EPI_ISL_7971547, EPI_ISL_7971548, EPI_ISL_7971549, EPI_ISL_7971550, EPI_ISL_7971551, EPI_ISL_7971552, EPI_ISL_7971553, EPI_ISL_7971554, EPI_ISL_7971555, EPI_ISL_7971556			
see above	VERMAAK LABORATORY	National Institute for Communicable Diseases of the National Health Laboratory Service	Amoako DG; Bhiman JN; Everatt J; Ismail A; Kekana D; Mahlangu B; Mnguni A; Mohale T; Ntuli N; Scheepers C; Wolter N
EPI_ISL_2494710, EPI_ISL_2494715, EPI_ISL_2494717, EPI_ISL_2494718, EPI_ISL_2494719, EPI_ISL_2494723, EPI_ISL_2494724, EPI_ISL_2494726, EPI_ISL_2494732, EPI_ISL_2494733, EPI_ISL_2494735, EPI_ISL_2494737, EPI_ISL_2494742, EPI_ISL_2494754, EPI_ISL_2494757, EPI_ISL_2494760, EPI_ISL_2494765, EPI_ISL_2494768, EPI_ISL_2494788, EPI_ISL_2494902, EPI_ISL_2494919, EPI_ISL_2494933, EPI_ISL_2494941, EPI_ISL_2494947, EPI_ISL_2494962, EPI_ISL_2494967, EPI_ISL_2494968, EPI_ISL_2494970, EPI_ISL_2494973, EPI_ISL_2494974, EPI_ISL_2494976, EPI_ISL_2494981, EPI_ISL_2494987, EPI_ISL_2494988, EPI_ISL_2494991, EPI_ISL_2494996, EPI_ISL_2494997, EPI_ISL_2495000, EPI_ISL_2495001, EPI_ISL_2495002, EPI_ISL_2495004, EPI_ISL_2495006, EPI_ISL_2495010, EPI_ISL_2495011, EPI_ISL_2495015, EPI_ISL_2495018, EPI_ISL_2495019, EPI_ISL_2495020, EPI_ISL_2495022, EPI_ISL_2495024, EPI_ISL_2495030, EPI_ISL_2495031, EPI_ISL_2495033, EPI_ISL_3447812, EPI_ISL_3447814, EPI_ISL_3447815, EPI_ISL_3447816, EPI_ISL_3447823, EPI_ISL_3447825, EPI_ISL_3447843, EPI_ISL_3447844, EPI_ISL_3447845, EPI_ISL_3447846, EPI_ISL_3447847, EPI_ISL_3447848, EPI_ISL_3447849, EPI_ISL_3447852, EPI_ISL_3447853, EPI_ISL_3447854, EPI_ISL_3447863, EPI_ISL_3447864, EPI_ISL_3447865, EPI_ISL_3447866, EPI_ISL_3447867, EPI_ISL_3447868, EPI_ISL_3447881, EPI_ISL_3728985, EPI_ISL_3728989, EPI_ISL_3728993, EPI_ISL_3728996, EPI_ISL_3729000, EPI_ISL_3729005, EPI_ISL_3729009, EPI_ISL_3729013, EPI_ISL_3729016, EPI_ISL_3729021, EPI_ISL_3729024, EPI_ISL_3729028, EPI_ISL_3729032, EPI_ISL_3729036, EPI_ISL_3729047, EPI_ISL_3729054, EPI_ISL_3729072, EPI_ISL_3729076, EPI_ISL_3729080, EPI_ISL_3729101, EPI_ISL_3729106, EPI_ISL_3729115, EPI_ISL_3729118, EPI_ISL_3729124, EPI_ISL_3729125, EPI_ISL_3729126, EPI_ISL_3729131, EPI_ISL_3729132, EPI_ISL_3729138, EPI_ISL_3729142, EPI_ISL_3729145, EPI_ISL_3729146, EPI_ISL_3729147, EPI_ISL_3729150, EPI_ISL_3729153, EPI_ISL_3729164, EPI_ISL_3729183, EPI_ISL_3729192, EPI_ISL_3729196, EPI_ISL_3729203, EPI_ISL_3729208, EPI_ISL_3729212, EPI_ISL_3729216, EPI_ISL_3729220, EPI_ISL_3729227, EPI_ISL_3729235			
see above	Vaccines and Infectious Diseases Analytics Research Unit (VIDA)	KRISP, KZN Research Innovation and Sequencing Platform	Baillie Vicky; Giandhari Jennifer; Madhi Shabir; Naidoo Yeshnee; Pillay Sureshnee; San James; Tegally Houriiyah; Tshabuila Derek; Wilkinson Eduan; Yajna Ramphal; de Oliveira Tulio; du Plessis Jeanine
EPI_ISL_2360362, EPI_ISL_2360371, EPI_ISL_2360376, EPI_ISL_2360393, EPI_ISL_2360399, EPI_ISL_2360402, EPI_ISL_2360407, EPI_ISL_2360410, EPI_ISL_2360414, EPI_ISL_2360415, EPI_ISL_2360416, EPI_ISL_2360417, EPI_ISL_2360468, EPI_ISL_2360519, EPI_ISL_2360520, EPI_ISL_2360521, EPI_ISL_2360522, EPI_ISL_2360523, EPI_ISL_2360531, EPI_ISL_2360533, EPI_ISL_2360536, EPI_ISL_2360537, EPI_ISL_2360538, EPI_ISL_2360539, EPI_ISL_2360540, EPI_ISL_2360541, EPI_ISL_2360542, EPI_ISL_2360544, EPI_ISL_2360545, EPI_ISL_2360546, EPI_ISL_2360548, EPI_ISL_2360549, EPI_ISL_2360555, EPI_ISL_2360556, EPI_ISL_2360558, EPI_ISL_2360560, EPI_ISL_2360561, EPI_ISL_2360562, EPI_ISL_2360564, EPI_ISL_2360567, EPI_ISL_2360568, EPI_ISL_2360569, EPI_ISL_2360577, EPI_ISL_2360578, EPI_ISL_2360579, EPI_ISL_2360582, EPI_ISL_2360583, EPI_ISL_2360584, EPI_ISL_2360585, EPI_ISL_2360588, EPI_ISL_2360590, EPI_ISL_2360599, EPI_ISL_2360604, EPI_ISL_2360605, EPI_ISL_2360610, EPI_ISL_2360611, EPI_ISL_2360615, EPI_ISL_2360618, EPI_ISL_2360619, EPI_ISL_2360622, EPI_ISL_2360623, EPI_ISL_2360624, EPI_ISL_2360625, EPI_ISL_2360626, EPI_ISL_2360627, EPI_ISL_2360629, EPI_ISL_2360630, EPI_ISL_2360648, EPI_ISL_2360650, EPI_ISL_2360656, EPI_ISL_2360657, EPI_ISL_2360668, EPI_ISL_2360672, EPI_ISL_2360673, EPI_ISL_2360674, EPI_ISL_2360685, EPI_ISL_2360686, EPI_ISL_2360688, EPI_ISL_2360691, EPI_ISL_2360696, EPI_ISL_2360700, EPI_ISL_2360710, EPI_ISL_2360715, EPI_ISL_2360720, EPI_ISL_2360727, EPI_ISL_2360728, EPI_ISL_2360729, EPI_ISL_2360734, EPI_ISL_2360747, EPI_ISL_2360748, EPI_ISL_2360749, EPI_ISL_2360750, EPI_ISL_2360751, EPI_ISL_2360752, EPI_ISL_2360753, EPI_ISL_2360754, EPI_ISL_2360755, EPI_ISL_2360756, EPI_ISL_2360757, EPI_ISL_2360759, EPI_ISL_2360760, EPI_ISL_2360767, EPI_ISL_2360769, EPI_ISL_2360770, EPI_ISL_2360777, EPI_ISL_2360779, EPI_ISL_2360780, EPI_ISL_2360781, EPI_ISL_2360790, EPI_ISL_2360791, EPI_ISL_2360793, EPI_ISL_2360798, EPI_ISL_2360799, EPI_ISL_2360801, EPI_ISL_2360802, EPI_ISL_2360804, EPI_ISL_2360805, EPI_ISL_2360806, EPI_ISL_2360807, EPI_ISL_2360808, EPI_ISL_2360810, EPI_ISL_2360811, EPI_ISL_2360812, EPI_ISL_2360813, EPI_ISL_2360814, EPI_ISL_2360815, EPI_ISL_2360819, EPI_ISL_2360824, EPI_ISL_2360827, EPI_ISL_2360830, EPI_ISL_2360831, EPI_ISL_2360832, EPI_ISL_2360834, EPI_ISL_2360835, EPI_ISL_2360855, EPI_ISL_2360857, EPI_ISL_2360859, EPI_ISL_2360860, EPI_ISL_2360861, EPI_ISL_2360867, EPI_ISL_2360869, EPI_ISL_2360870, EPI_ISL_2360873, EPI_ISL_2360874, EPI_ISL_2360875, EPI_ISL_2360879, EPI_ISL_2360880, EPI_ISL_2360884, EPI_ISL_2360891, EPI_ISL_2360901, EPI_ISL_2360906, EPI_ISL_2360911, EPI_ISL_2360912, EPI_ISL_2360913, EPI_ISL_2360922, EPI_ISL_2360927, EPI_ISL_2360930, EPI_ISL_2360931, EPI_ISL_2360932, EPI_ISL_2360933, EPI_ISL_2360937, EPI_ISL_2360939, EPI_ISL_2360945, EPI_ISL_2360946, EPI_ISL_2360947, EPI_ISL_2360952, EPI_ISL_2360953, EPI_ISL_2360956, EPI_ISL_2360957, EPI_ISL_2360958, EPI_ISL_2360959, EPI_ISL_2360960, EPI_ISL_2360961, EPI_ISL_2360965, EPI_ISL_2360967, EPI_ISL_2360968, EPI_ISL_2360969, EPI_ISL_2360970, EPI_ISL_2360971, EPI_ISL_2360972, EPI_ISL_2360975, EPI_ISL_2360977, EPI_ISL_2360978, EPI_ISL_2360980, EPI_ISL_2360988, EPI_ISL_2360989, EPI_ISL_2360990, EPI_ISL_2360991, EPI_ISL_2360992, EPI_ISL_2360994, EPI_ISL_2360995, EPI_ISL_2360996, EPI_ISL_2360997, EPI_ISL_2360998, EPI_ISL_2361005, EPI_ISL_2361006, EPI_ISL_2361007, EPI_ISL_2361008, EPI_ISL_2361010, EPI_ISL_2361012, EPI_ISL_2361015, EPI_ISL_2361016, EPI_ISL_2361017, EPI_ISL_2361019, EPI_ISL_2361020, EPI_ISL_2361021, EPI_ISL_2361022, EPI_ISL_2361023, EPI_ISL_2361025, EPI_ISL_2361029, EPI_ISL_2361030, EPI_ISL_2361031, EPI_ISL_2361032, EPI_ISL_2361033, EPI_ISL_2361034, EPI_ISL_2361035, EPI_ISL_2361046, EPI_ISL_2361059, EPI_ISL_2361062, EPI_ISL_2361063, EPI_ISL_2361064, EPI_ISL_2361065, EPI_ISL_2361066, EPI_ISL_2361068, EPI_ISL_2494357, EPI_ISL_2494359, EPI_ISL_2494360, EPI_ISL_2494365, EPI_ISL_2494366, EPI_ISL_2494367, EPI_ISL_2494371, EPI_ISL_2494372, EPI_ISL_2494373, EPI_ISL_2494378, EPI_ISL_2494379, EPI_ISL_2494380, EPI_ISL_2494381, EPI_ISL_2494382, EPI_ISL_2494388, EPI_ISL_2494391, EPI_ISL_2494392, EPI_ISL_2494394, EPI_ISL_2494396, EPI_ISL_2494398, EPI_ISL_2494402, EPI_ISL_2494405, EPI_ISL_2494407, EPI_ISL_2494409, EPI_ISL_2494412, EPI_ISL_2494413, EPI_ISL_2494416, EPI_ISL_2494420, EPI_ISL_2494423, EPI_ISL_2494425, EPI_ISL_2494426, EPI_ISL_2494429, EPI_ISL_2494430, EPI_ISL_2494432, EPI_ISL_2494433, EPI_ISL_2494434, EPI_ISL_2494435, EPI_ISL_2494436, EPI_ISL_2494438, EPI_ISL_2494439, EPI_ISL_2494442, EPI_ISL_2494443, EPI_ISL_2494444, EPI_ISL_2494448, EPI_ISL_2494456, EPI_ISL_2494460, EPI_ISL_2494461, EPI_ISL_2494466, EPI_ISL_2494468, EPI_ISL_2494471, EPI_ISL_2494472, EPI_ISL_2494478, EPI_ISL_2494479, EPI_ISL_2494482, EPI_ISL_2494484, EPI_ISL_2494487, EPI_ISL_2494488, EPI_ISL_2494489, EPI_ISL_2494494, EPI_ISL_2494495, EPI_ISL_2494512, EPI_ISL_2494513, EPI_ISL_2494526, EPI_ISL_2494528, EPI_ISL_2494529, EPI_ISL_2494534, EPI_ISL_2494537, EPI_ISL_2494539, EPI_ISL_2494549, EPI_ISL_2494552, EPI_ISL_2494553, EPI_ISL_2494558, EPI_ISL_2494559, EPI_ISL_2494562, EPI_ISL_2494563, EPI_ISL_2494567, EPI_ISL_2494575, EPI_ISL_2494577, EPI_ISL_2494578, EPI_ISL_2494580, EPI_ISL_2494582, EPI_ISL_2494586, EPI_ISL_2494589, EPI_ISL_2494590, EPI_ISL_2494591, EPI_ISL_2494593, EPI_ISL_2494594, EPI_ISL_2494601, EPI_ISL_2494603, EPI_ISL_2494609, EPI_ISL_2494616, EPI_ISL_2494627, EPI_ISL_2494630			
see above	Vaccines and Infectious Diseases Analytics Research Unit (VIDA)	KRISP, KZn Research Innovation and Sequencing Platform	Baillie Vicky; Giandhari Jennifer; Madhi Shabir; Naidoo Yeshnee; Pillay Sureshnee; San James; Tegally Houriiyah; Tshabuila Derek; Wilkinson Eduan; de Oliveira Tulio; du Plessis Jeanine
EPI_ISL_940850, EPI_ISL_940851, EPI_ISL_940852, EPI_ISL_940853, EPI_ISL_940854, EPI_ISL_940855, EPI_ISL_940856, EPI_ISL_940858, EPI_ISL_940859, EPI_ISL_940861, EPI_ISL_940862, EPI_ISL_940863, EPI_ISL_940865, EPI_ISL_940866, EPI_ISL_940867, EPI_ISL_940868, EPI_ISL_940869, EPI_ISL_940870, EPI_ISL_940871, EPI_ISL_940874, EPI_ISL_940875, EPI_ISL_940876, EPI_ISL_940877, EPI_ISL_940878, EPI_ISL_940879, EPI_ISL_940880, EPI_ISL_940881, EPI_ISL_940882, EPI_ISL_940884, EPI_ISL_940885, EPI_ISL_940886, EPI_ISL_940887, EPI_ISL_940888, EPI_ISL_940889, EPI_ISL_940890, EPI_ISL_940891			
see above	Vaccines and Infectious Diseases Analytics Research Unit (VIDA)	KRISP, KZN Research Innovation and Sequencing Platform	Baillie Vicky; Giandhari Jennifer; Madhi Shabir; Naidoo Yeshnee; Pillay Sureshnee; Tegally Houriiyah; de Oliveira Tulio; du Plessis Jeanine
EPI_ISL_1132669, EPI_ISL_1132670, EPI_ISL_1132671, EPI_ISL_1132672, EPI_ISL_1132673, EPI_ISL_1132674, EPI_ISL_1132675, EPI_ISL_1132676, EPI_ISL_1132677, EPI_ISL_1132678, EPI_ISL_1132679, EPI_ISL_1132680, EPI_ISL_1132681, EPI_ISL_1132682, EPI_ISL_1132683, EPI_ISL_1132684, EPI_ISL_1132685, EPI_ISL_1132686, EPI_ISL_1132687, EPI_ISL_1132688, EPI_ISL_1132689, EPI_ISL_1132690, EPI_ISL_1132691, EPI_ISL_1132692, EPI_ISL_1132693, EPI_ISL_1132694, EPI_ISL_1132695, EPI_ISL_1132696, EPI_ISL_1132697, EPI_ISL_1132698, EPI_ISL_1132699, EPI_ISL_1132700, EPI_ISL_1132701, EPI_ISL_1132702, EPI_ISL_1132703, EPI_ISL_1132704, EPI_ISL_1132705, EPI_ISL_1132706, EPI_ISL_1132707, EPI_ISL_1132708, EPI_ISL_1132709, EPI_ISL_1132710, EPI_ISL_1132711, EPI_ISL_1132712, EPI_ISL_1132713, EPI_ISL_1132714, EPI_ISL_1132715, EPI_ISL_1132716, EPI_ISL_1132717, EPI_ISL_1132718, EPI_ISL_1132719, EPI_ISL_1132720, EPI_ISL_1132721, EPI_ISL_1132722, EPI_ISL_1132723, EPI_ISL_1132724, EPI_ISL_1132725, EPI_ISL_1132726, EPI_ISL_1132727, EPI_ISL_1132728			
see above	Vaccines and Infectious Diseases Analytics Research Unit (VIDA)	KRISP, KZn Research Innovation and Sequencing Platform	Baillie Vicky; Giandhari Jennifer; Madhi Shabir; Naidoo Yeshnee; Pillay Sureshnee; Tegally Houriiyah; de Oliveira Tulio; du Plessis Jeanine
EPI_ISL_5917944, EPI_ISL_5917979, EPI_ISL_5918019, EPI_ISL_5918027	Vermaak	National Institute for Communicable Diseases of the National Health Laboratory Service	Amoako DG; Bhiman JN; Everatt J; Ismail A; Mahlangu B; Mnguni A; Mohale T; Ntuli N; Scheepers C
EPI_ISL_2086968, EPI_ISL_2086969, EPI_ISL_2086972, EPI_ISL_2086979, EPI_ISL_2086981, EPI_ISL_2086983, EPI_ISL_2582726, EPI_ISL_2582730, EPI_ISL_2582733, EPI_ISL_2582736, EPI_ISL_2582739, EPI_ISL_2582742, EPI_ISL_2582744, EPI_ISL_2582746, EPI_ISL_2582748, EPI_ISL_2582749, EPI_ISL_2582752, EPI_ISL_2582757, EPI_ISL_2582761, EPI_ISL_2582765, EPI_ISL_2582769, EPI_ISL_2582772, EPI_ISL_2582774, EPI_ISL_2582776, EPI_ISL_2582781, EPI_ISL_2582786, EPI_ISL_2582790, EPI_ISL_2582792, EPI_ISL_2582794, EPI_ISL_2582797, EPI_ISL_2582800, EPI_ISL_2582803, EPI_ISL_2582806			
see above	Vermaak & Partners Laboratory	National Institute for Communicable Diseases of the National Health Laboratory Service	Amoako DG; Bhiman JN; Everatt J; Ismail A; Mahlangu B; Mnguni A; Mohale T; Ntuli N; Scheepers C
EPI_ISL_2841660, EPI_ISL_2841661, EPI_ISL_2841662, EPI_ISL_2841663, EPI_ISL_2841664, EPI_ISL_2841665, EPI_ISL_2841666, EPI_ISL_2841667, EPI_ISL_2841668, EPI_ISL_2841669, EPI_ISL_2841670, EPI_ISL_2841671, EPI_ISL_2841672, EPI_ISL_2841673, EPI_ISL_2841674, EPI_ISL_2841675, EPI_ISL_2841676, EPI_ISL_2841677, EPI_ISL_2841678, EPI_ISL_2841679, EPI_ISL_2841680, EPI_ISL_2841681, EPI_ISL_2841682, EPI_ISL_2841683, EPI_ISL_2841684, EPI_ISL_2841685, EPI_ISL_2841686, EPI_ISL_2841687, EPI_ISL_2841688, EPI_ISL_2841689, EPI_ISL_2841690, EPI_ISL_2841691, EPI_ISL_2841692, EPI_ISL_2841693, EPI_ISL_2841694, EPI_ISL_2841695, EPI_ISL_2841696, EPI_ISL_2841697, EPI_ISL_2841698, EPI_ISL_2841699, EPI_ISL_2841700			
see above	Wits Vaccines & Infectious Diseases Analytics (VIDA) Research Unit	KRISP, KZn Research Innovation and Sequencing Platform	Baillie Vicky; Giandhari Jennifer; Madhi Shabir; Naidoo Yeshnee; Pillay Sureshnee; San James; Tegally Houriiyah; Wilkinson Eduan; de Oliveira Tulio; du Plessis Jeanine

EPI_ISL_6261993	ZARV, Department Medical Virology, University of Pretoria	ZARV, Department Mdeical Virology, University of Pretoria	Adriano Mendes; Amy Strydom; Katja Koeppel; Lia Rotherham and Marietjie Venter
EPI_ISL_6261983, EPI_ISL_6261987, EPI_ISL_6261989, EPI_ISL_6261996	ZARV, Department Medical Virology, University of Pretoria	ZARV, Department Medical Virology, University of Pretoria	Adriano Mendes; Amy Strydom; Katja Koeppel; Lia Rotherham and Marietjie Venter
EPI_ISL_4474416, EPI_ISL_4474417, EPI_ISL_4474418, EPI_ISL_4474419, EPI_ISL_4474420, EPI_ISL_4474421, EPI_ISL_4474422, EPI_ISL_4474423, EPI_ISL_4474424, EPI_ISL_4474425, EPI_ISL_4474426, EPI_ISL_4474427, EPI_ISL_4474428, EPI_ISL_4474429, EPI_ISL_4474430, EPI_ISL_4474431, EPI_ISL_4474432			
see above	ZARV/NHLS, Department Medical Virology, University of Pretoria	CERI, Centre for Epidemic Response and Innovation	Adriano Mendes; Amy Strydom; Emmanuel S; Giandhari J; Micheala Davids; Naidoo Yeshnee; Pillay S; Sim Mayaphi and Marietjie Venter; Tegally H; Tshabuila Derek; Wilkinson E; Yajna Ramphal; de Oliveira T
EPI_ISL_5098761, EPI_ISL_5098766, EPI_ISL_5098771, EPI_ISL_5098774, EPI_ISL_5098782, EPI_ISL_5098787, EPI_ISL_5098801, EPI_ISL_5098860, EPI_ISL_5098870, EPI_ISL_5098876, EPI_ISL_5098882, EPI_ISL_5098912, EPI_ISL_5098921, EPI_ISL_5098949, EPI_ISL_5098984, EPI_ISL_5098990, EPI_ISL_5099007, EPI_ISL_5099083, EPI_ISL_6795833, EPI_ISL_6795834, EPI_ISL_6795835, EPI_ISL_6795836, EPI_ISL_6795837, EPI_ISL_6795838, EPI_ISL_6795839, EPI_ISL_6795840, EPI_ISL_6795841, EPI_ISL_6795842, EPI_ISL_6795843, EPI_ISL_6795844, EPI_ISL_6795845, EPI_ISL_6795846, EPI_ISL_6795847, EPI_ISL_6795848, EPI_ISL_6795849, EPI_ISL_6795850, EPI_ISL_6825389, EPI_ISL_6825390, EPI_ISL_6825391, EPI_ISL_6825392, EPI_ISL_6825393, EPI_ISL_6825394, EPI_ISL_6825395, EPI_ISL_6825396, EPI_ISL_6825397, EPI_ISL_6825398, EPI_ISL_7015171, EPI_ISL_7015172, EPI_ISL_7015173, EPI_ISL_7015174, EPI_ISL_7015175, EPI_ISL_7015176, EPI_ISL_7015177, EPI_ISL_7015178, EPI_ISL_7015179, EPI_ISL_7015180, EPI_ISL_7015181, EPI_ISL_7015182, EPI_ISL_7015183, EPI_ISL_7015184, EPI_ISL_7015185, EPI_ISL_7015186, EPI_ISL_7015187, EPI_ISL_7015188, EPI_ISL_7015189, EPI_ISL_7015190, EPI_ISL_7015191, EPI_ISL_7015192, EPI_ISL_7015193, EPI_ISL_7015194, EPI_ISL_7015195, EPI_ISL_7015196, EPI_ISL_7015197, EPI_ISL_7015198, EPI_ISL_7015199, EPI_ISL_7015200, EPI_ISL_7015201, EPI_ISL_7015202, EPI_ISL_7015203, EPI_ISL_7015204, EPI_ISL_7015205, EPI_ISL_7015206, EPI_ISL_7015207, EPI_ISL_7015208, EPI_ISL_8128432, EPI_ISL_8128433, EPI_ISL_8128434, EPI_ISL_8128435, EPI_ISL_8128436, EPI_ISL_8128437, EPI_ISL_8128438, EPI_ISL_8128439, EPI_ISL_8128440, EPI_ISL_8128441, EPI_ISL_8128442, EPI_ISL_8128443, EPI_ISL_8128444, EPI_ISL_8128445, EPI_ISL_8128446, EPI_ISL_8128447, EPI_ISL_8128448, EPI_ISL_8128449, EPI_ISL_8128450, EPI_ISL_8128451, EPI_ISL_8128452, EPI_ISL_8128453, EPI_ISL_8128454, EPI_ISL_8128455, EPI_ISL_8128456, EPI_ISL_8128457, EPI_ISL_8128458, EPI_ISL_8128459, EPI_ISL_8128460, EPI_ISL_8128461, EPI_ISL_8128462, EPI_ISL_8128463, EPI_ISL_8128464, EPI_ISL_8128465, EPI_ISL_8128466, EPI_ISL_8128467, EPI_ISL_8128468, EPI_ISL_8128469, EPI_ISL_8128470, EPI_ISL_8128471, EPI_ISL_8128472, EPI_ISL_8128473, EPI_ISL_8128474, EPI_ISL_8128475, EPI_ISL_8128476, EPI_ISL_8128477, EPI_ISL_8128478, EPI_ISL_8128479, EPI_ISL_8128480, EPI_ISL_8128481, EPI_ISL_8128482, EPI_ISL_8128483, EPI_ISL_8128484, EPI_ISL_8128485, EPI_ISL_8128486, EPI_ISL_8128487, EPI_ISL_8128488, EPI_ISL_8128489, EPI_ISL_8128490, EPI_ISL_8128491, EPI_ISL_8128492, EPI_ISL_8128493, EPI_ISL_8128494, EPI_ISL_8128495, EPI_ISL_8128496, EPI_ISL_8128497, EPI_ISL_8128498, EPI_ISL_8128499, EPI_ISL_8128500, EPI_ISL_8128501, EPI_ISL_8128502, EPI_ISL_8128503, EPI_ISL_8128504			
see above	ZARV/NHLS, Department Medical Virology, University of Pretoria	CERI, Centre for Epidemic Response and Innvoation, Stellenbosch University and KRISP, KZN Research Innovation and Sequencing Platform, UKZN.	Adriano Mendes; Amoaka D; Amy Strydom; Arisha Maharaj; Bester P; Bhiman J; Engelbrecht S; Everatt J; Giandhari J; Giandhari Jennifer; Goedhals D; Hardie D; Hsiao M; Iranzadeh A; Lessells R; Makatini Z; Maponga T; Mdlalose N; Micheala Davids; Mlisana K; Moir M; NGS-SA (Scheepers C; Naidoo Y; Naidoo Yeshnee; Nyaga M) Giandhari J; Oluwakemi M; Pillay S; Pillay Sureshnee; Preiser W; Ramphal U; Ramphal Y; San JE; San James; Sim Mayaphi and Marietjie Venter; Tegally H; Tegally Houriiyah; Tshiabuila D; Tshiabuila Derek; Upasana Ramphal; Venter M; Wilkinson E; Wilkinson Eduan; Williamson C; Yajna Ramphal; de Oliveira T; de Oliveira Tulio; von Gottberg A
EPI_ISL_2727239, EPI_ISL_2727240, EPI_ISL_2727241, EPI_ISL_2727242, EPI_ISL_2727243, EPI_ISL_2727244, EPI_ISL_2727245, EPI_ISL_2727246, EPI_ISL_2727247, EPI_ISL_2727248, EPI_ISL_2727249, EPI_ISL_2727250, EPI_ISL_2727251, EPI_ISL_2727252, EPI_ISL_2727253, EPI_ISL_2727254, EPI_ISL_2727255, EPI_ISL_2727256, EPI_ISL_2727257, EPI_ISL_2727258, EPI_ISL_2727259, EPI_ISL_2727260, EPI_ISL_2727261, EPI_ISL_2727262, EPI_ISL_2727263, EPI_ISL_2727264, EPI_ISL_2727265, EPI_ISL_2727266, EPI_ISL_2727267, EPI_ISL_2727268, EPI_ISL_2727269, EPI_ISL_2727270, EPI_ISL_2727271, EPI_ISL_2727272, EPI_ISL_2727273, EPI_ISL_2727274, EPI_ISL_2727275, EPI_ISL_2727276, EPI_ISL_2727277, EPI_ISL_2727278, EPI_ISL_2727279, EPI_ISL_2727280, EPI_ISL_2727281, EPI_ISL_2727282, EPI_ISL_2727283, EPI_ISL_2727284, EPI_ISL_2727285, EPI_ISL_2727286, EPI_ISL_2727287, EPI_ISL_2727288, EPI_ISL_2727289, EPI_ISL_2727290, EPI_ISL_4572282, EPI_ISL_4572283, EPI_ISL_4572284, EPI_ISL_4572285, EPI_ISL_4572286, EPI_ISL_4572287, EPI_ISL_4572288, EPI_ISL_4572289, EPI_ISL_4572290, EPI_ISL_4572291, EPI_ISL_4572292, EPI_ISL_4572293, EPI_ISL_4572294, EPI_ISL_4572295, EPI_ISL_4572319, EPI_ISL_4572322, EPI_ISL_4572323, EPI_ISL_4572324, EPI_ISL_4572329, EPI_ISL_4572331, EPI_ISL_4572333, EPI_ISL_4572334, EPI_ISL_4572338, EPI_ISL_4572339, EPI_ISL_4572342, EPI_ISL_4572343, EPI_ISL_4572353, EPI_ISL_4572356, EPI_ISL_4572357, EPI_ISL_4572358, EPI_ISL_4572360, EPI_ISL_4572361, EPI_ISL_4572364, EPI_ISL_4572366, EPI_ISL_4572367, EPI_ISL_4572368, EPI_ISL_4572373, EPI_ISL_4572374, EPI_ISL_4572378, EPI_ISL_4572379, EPI_ISL_4572387, EPI_ISL_4572388, EPI_ISL_4575028, EPI_ISL_4575029, EPI_ISL_4575030, EPI_ISL_4575031, EPI_ISL_4575032, EPI_ISL_4575033, EPI_ISL_4575034, EPI_ISL_4575035, EPI_ISL_4575036, EPI_ISL_4575037, EPI_ISL_4575038, EPI_ISL_4575039, EPI_ISL_4575040, EPI_ISL_4575041, EPI_ISL_4575042, EPI_ISL_4575043, EPI_ISL_4575044, EPI_ISL_4575045, EPI_ISL_4575046, EPI_ISL_4575047, EPI_ISL_4575048, EPI_ISL_4575049, EPI_ISL_4575050, EPI_ISL_4575051, EPI_ISL_4575052, EPI_ISL_4575053, EPI_ISL_4575054, EPI_ISL_4575060, EPI_ISL_4575070, EPI_ISL_4575071, EPI_ISL_4575079, EPI_ISL_4575080, EPI_ISL_4575081, EPI_ISL_4575082, EPI_ISL_4575084, EPI_ISL_4575088, EPI_ISL_4575089, EPI_ISL_4575098, EPI_ISL_4575100, EPI_ISL_4575102, EPI_ISL_4575107, EPI_ISL_4575119, EPI_ISL_4575120, EPI_ISL_4575121, EPI_ISL_4575127, EPI_ISL_4575128, EPI_ISL_4575129, EPI_ISL_4575130, EPI_ISL_4575133, EPI_ISL_4575141, EPI_ISL_4575142, EPI_ISL_4575150, EPI_ISL_4575151, EPI_ISL_4575153, EPI_ISL_4575154, EPI_ISL_4575155, EPI_ISL_4575156, EPI_ISL_4575157, EPI_ISL_4575164, EPI_ISL_4575165, EPI_ISL_4575166, EPI_ISL_4575167, EPI_ISL_4575169, EPI_ISL_4575170, EPI_ISL_4575171, EPI_ISL_4575183, EPI_ISL_4575184, EPI_ISL_4575185, EPI_ISL_4575189, EPI_ISL_4575190, EPI_ISL_4575191, EPI_ISL_4575200, EPI_ISL_4575203			
see above	ZARV/NHLS, Department Medical Virology, University of Pretoria	KRISP, KZN Research Innovation and Sequencing Platform	Adriano Mendes; Amy Strydom; Emmanuel SJ; Giandhari J; Giandhari Jennifer; Lessells R; Micheala Davids; Naidoo Y; Naidoo Yeshnee; Pillay S; Pillay Sureshnee; Ramphal U; San James; Sim Mayaphi and Marietjie Venter; Tegally H; Tegally Houriiyah; Tshiabuila Derek; Wilkinson E; Wilkinson Eduan; Yajna Ramphal; de Oliveira T; de Oliveira Tulio
EPI_ISL_2727328, EPI_ISL_2727329, EPI_ISL_2727330, EPI_ISL_2727331, EPI_ISL_2727332, EPI_ISL_2727333, EPI_ISL_2727334, EPI_ISL_2727335, EPI_ISL_2727336, EPI_ISL_2727337, EPI_ISL_2727338, EPI_ISL_2727339, EPI_ISL_2727340, EPI_ISL_2727341, EPI_ISL_2727342, EPI_ISL_2727343, EPI_ISL_2727344, EPI_ISL_2727345, EPI_ISL_2727346, EPI_ISL_2727347, EPI_ISL_2727348, EPI_ISL_2727349, EPI_ISL_2727350, EPI_ISL_2727351, EPI_ISL_2727352, EPI_ISL_2727353, EPI_ISL_2727354, EPI_ISL_2727355, EPI_ISL_2727356, EPI_ISL_2727357, EPI_ISL_2727358, EPI_ISL_2727359, EPI_ISL_2727360, EPI_ISL_2727361, EPI_ISL_2727362, EPI_ISL_2727363, EPI_ISL_2727364, EPI_ISL_2727365, EPI_ISL_2727366, EPI_ISL_2727367, EPI_ISL_2727368, EPI_ISL_2727369, EPI_ISL_2727370, EPI_ISL_2727371, EPI_ISL_2727372, EPI_ISL_2727373, EPI_ISL_2727374, EPI_ISL_2727375, EPI_ISL_2727376, EPI_ISL_2727377, EPI_ISL_2727378, EPI_ISL_2727379, EPI_ISL_2899741, EPI_ISL_2899742, EPI_ISL_2899743, EPI_ISL_2899744, EPI_ISL_2899745, EPI_ISL_2899746, EPI_ISL_2899747, EPI_ISL_2899748, EPI_ISL_2899749, EPI_ISL_2899750, EPI_ISL_2899751, EPI_ISL_2899752, EPI_ISL_2899753, EPI_ISL_2899754, EPI_ISL_2899755, EPI_ISL_2899756, EPI_ISL_2899757, EPI_ISL_2955421, EPI_ISL_2955422, EPI_ISL_2955423, EPI_ISL_2955424, EPI_ISL_2955425, EPI_ISL_2955426, EPI_ISL_2955427, EPI_ISL_2955428, EPI_ISL_2955429, EPI_ISL_2955430, EPI_ISL_2955431, EPI_ISL_2955432, EPI_ISL_2955433, EPI_ISL_2955434, EPI_ISL_2955435, EPI_ISL_2955436, EPI_ISL_2955437, EPI_ISL_2955438, EPI_ISL_2955439, EPI_ISL_2955440, EPI_ISL_2955441, EPI_ISL_2955442, EPI_ISL_2955443, EPI_ISL_2955444, EPI_ISL_2955445, EPI_ISL_2955446, EPI_ISL_2955447, EPI_ISL_2955448, EPI_ISL_2955449, EPI_ISL_2955450, EPI_ISL_2955451, EPI_ISL_2955452, EPI_ISL_4121618, EPI_ISL_4121678, EPI_ISL_4121684			
see above	ZARV/NHLS, Department Medical Virology, University of Pretoria	KRISP, KZn Research Innovation and Sequencing Platform	Adriano Mendes; Amy Strydom; Emmanuel SJ; Giandhari J; Lessells R; Micheala Davids; Naidoo Y; Pillay S; Ramphal U; Sim Mayaphi and Marietjie Venter; Tegally H; Wilkinson E; de Oliveira T
EPI_ISL_5510403	ZONE 3 CLINIC	National Institute for Communicable Diseases of the National Health Laboratory Service	Amoako DG; Bhiman JN; Everatt J; Ismail A; Mahlangu B; Mnguni A; Mohale T; Ntuli N; Scheepers C
