# Supplementary material for: The Spread of SARS-CoV-2 Variant Omicron with a Doubling Time of 2.0–3.3 Days Can Be Explained by Immune Evasion
Source: Viruses. 2022 Jan 30;14(2):294. doi: 10.3390/v14020294 (PMC8875689; doi:10.3390/v14020294)
Supplement: Supplementary file 1 [file viruses-14-00294-s001.zip › Table_S4--GISAID-KwaZulu-Natal-acknowledgements.pdf]

We gratefully acknowledge the following Authors from the Originating laboratories responsible for obtaining the specimens, as well as the Submitting laboratories where the genome data were generated and shared via GISAID, on which this research is based.

All Submitters of data may be contacted directly via www.gisaid.org

Authors are sorted alphabetically.

Accession ID	Originating Laboratory	Submitting Laboratory	Authors
EPI_ISL_7381191	AHRI	CERI, Centre for Epidemic Response and Innvoation, Stellenbosch University and KRISP, KZN Research Innovation and Sequencing Platform, UKZN.	Arisha Maharaj; Bernstein Mallory; Cele Sandile; Giandhari J; Karim Farina; Khan Khadija; Moir M; Naidoo Y; Pillay S; Ramphal U; Ramphal Y; San JE; Sigal Alex; Tegally H; Tshiabuila D; Wilkinson E; de Oliveira T; van Wyk S
EPI_ISL_2397308, EPI_ISL_2397309, EPI_ISL_2397310, EPI_ISL_2397311, EPI_ISL_2397312, EPI_ISL_2397313	AHRI	KRISP, KZN Research Innovation and Sequencing Platform	Bernstein M; COMMIT-KZN Team; Cele S; Giandhari J; Gosnell BI; Hanekom W; Karim F; Khan K; Lessells RJ; Manickchund N; Mlisana K; Moosa MYS; Msomi N; Pillay S; Ramphal U; San JE; Sigal A; Singh L; Tegally H; Wilkinson E; de Oliveira T
EPI_ISL_3722334, EPI_ISL_3722335, EPI_ISL_3722336, EPI_ISL_3722337, EPI_ISL_3722338, EPI_ISL_3722339	AHRI	KRISP, KZn Research Innovation and Sequencing Platform	Bernstein Mallory; Cele Sandile; Emmanuel S; Giandhari J; Karim Farina; Khan Khadija; Naidoo Yeshnee; Pillay S; Sigal Alex; Tegally H; Tshabuila Derek; Wilkinson E; Yajna Ramphal; de Oliveira T
EPI_ISL_3939064, EPI_ISL_3939068, EPI_ISL_3939088	AHRI - Sigal Lab	KRISP, KZn Research Innovation and Sequencing Platform	Bernstein Mallory; Cele Sandile; Emmanuel S; Giandhari J; Karim Farina; Khan Khadija; Naidoo Yeshnee; Pillay S; Sigal Alex; Tegally H; Tshabuila Derek; Wilkinson E; Yajna Ramphal; de Oliveira T
EPI_ISL_3447712, EPI_ISL_3447752, EPI_ISL_3447779, EPI_ISL_3447802	AHRI- Alex Sigal Lab	KRISP, KZN Research Innovation and Sequencing Platform	Alex Sigal; Giandhari Jennifer; Mallory Bernstein; Naidoo Yeshnee; Pillay Sureshnee; San E. James; Sandile Cele; Tegally Houriiyah; Tshabuila Derek; Wilkinson Eduan; Yajna Ramphal; de Oliveira Tulio
EPI_ISL_3267757	AHRI- Alex Sigal lab	KRISP, KZN Research Innovation and Sequencing Platform	Alex Sigal; Giandhari Jennifer; Mallory Bernstein; Naidoo Yeshnee; Pillay Sureshnee; San E. James; Sandile Cele; Tegally Houriiyah; Tshabuila Derek; Wilkinson Eduan; Yajna Ramphal; de Oliveira Tulio
EPI_ISL_1229367, EPI_ISL_1229368	AHRI-Sigal	KRISP, KZn Research Innovation and Sequencing Platform	Cele S; Gazy I; Giandhari J; Karim F; Pillay S; Sigal A; Tegally H; Wilkinson E; de Oliveira T
EPI_ISL_2493041	AMPATH	CERI, Centre for Epidemic Response and Innvoation, Stellenbosch University and KRISP, KZN Research Innovation and Sequencing Platform, UKZN.	Emmanuel SJ; Giandhari J; Khan S; Lessells R; Mdlalose K; Naidoo Y; Pillay S; Ramphal U; Tegally H; Wilkinson E; York D; de Oliveira T
EPI_ISL_2501090	AMPATH	KRISP, KZN Research Innovation and Sequencing Platform	Emmanuel SJ; Giandhari J; Khan S; Lessells R; Mdlalose K; Naidoo Y; Pillay S; Ramphal U; Sisonke Team; Tegally H; Wilkinson E; York D; de Oliveira T
EPI_ISL_3838572, EPI_ISL_3838573, EPI_ISL_3838574, EPI_ISL_3838575, EPI_ISL_3838576, EPI_ISL_3838577, EPI_ISL_4651553, EPI_ISL_4651560, EPI_ISL_6327732			
see above	AMPATH	National Institute for Communicable Diseases of the National Health Laboratory Service	Amoako DG; Bhiman JN; Everatt J; Ismail A; Mahlangu B; Mnguni A; Mohale T; Ntuli N; Scheepers C
EPI_ISL_2494961	Ampath	KRISP, KZN Research Innovation and Sequencing Platform	Emmanuel SJ; Giandhari J; Khan S; Lessells R; Mdlalose K; Naidoo Y; Pillay S; Ramphal U; Tegally H; Wilkinson E; York D; de Oliveira T
EPI_ISL_1994608	Ampath - Kingsway	KRISP, KZN Research Innovation and Sequencing Platform	Emmanuel SJ; Giandhari J; Khan S; Lessells R; Mdlalose K; Naidoo Y; Pillay S; Ramphal U; Tegally H; Wilkinson E; York D; de Oliveira T
EPI_ISL_2693088	Ampath laboratories- Sharks	KRISP, KZN Research Innovation and Sequencing Platform	Giandhari Jennifer; Naidoo Yeshnee; Pillay Sureshnee; San James; Tegally Houriiyah; Tshabuila Derek; Wilkinson Eduan; Yajna Ramphal; de Oliveira Tulio
EPI_ISL_860553	Ampath-Netcare	KRISP, KZn Research Innovation and Sequencing Platform	Giandhari J; Khan S; Lessells R; Mdlalose K; Pillay S; Tegally H; Wilkinson E; York D; de Oliveira T
EPI_ISL_2893315, EPI_ISL_2893316	BARC - Sisonke	KRISP, KZN Research Innovation and Sequencing Platform	Giandhari Jennifer; Naidoo Yeshnee; Pillay Sureshnee; San James; Sisonke; Tegally Houriiyah; Tshabuila Derek; Wilkinson Eduan; Yajna Ramphal; de Oliveira Tulio
EPI_ISL_2955501, EPI_ISL_2955502, EPI_ISL_2955503, EPI_ISL_2955504, EPI_ISL_2955505, EPI_ISL_2955506, EPI_ISL_2955507, EPI_ISL_2955508, EPI_ISL_2955509, EPI_ISL_2955510, EPI_ISL_2955511			
see above	CAPRISA	KRISP, KZn Research Innovation and Sequencing Platform	Emmanuel SJ; Giandhari J; Lessells R; Naidoo Y; Ngcapu S; Pillay S; Ramphal U; Samsunder N; Sivro A; Tegally H; Wilkinson E; de Oliveira T
EPI_ISL_7747505, EPI_ISL_7747506, EPI_ISL_7747507, EPI_ISL_7747508, EPI_ISL_7747509, EPI_ISL_7747510, EPI_ISL_7747511, EPI_ISL_7747512, EPI_ISL_7747513, EPI_ISL_7747514, EPI_ISL_7747515, EPI_ISL_7747516, EPI_ISL_7747517, EPI_ISL_7747518, EPI_ISL_7747519, EPI_ISL_7747520, EPI_ISL_7747521, EPI_ISL_7747522, EPI_ISL_7747523, EPI_ISL_7747524, EPI_ISL_7747525, EPI_ISL_7747526, EPI_ISL_7747527, EPI_ISL_7747528, EPI_ISL_7747529, EPI_ISL_7747530, EPI_ISL_7747531, EPI_ISL_7747532, EPI_ISL_7747533, EPI_ISL_7747534			
see above	CAPRISA_HILLCREST	CERI, Centre for Epidemic Response and Innvoation, Stellenbosch University and KRISP, KZN Research Innovation and Sequencing Platform, UKZN.	Aida Sivro; Arisha Maharaj; Giandhari J; Naidoo Y; Natasha Samsunder; Pillay S; Ramphal U; Ramphal Y; San JE; Tegally H; Tshiabuila D; Wilkinson E; de Oliveira T
EPI_ISL_2271972, EPI_ISL_2271982, EPI_ISL_2271990, EPI_ISL_2271991, EPI_ISL_2272019, EPI_ISL_2272020, EPI_ISL_2272021			
see above	Edendale Gateway Clinic	National Institute for Communicable Diseases of the National Health Laboratory Service	Amoako DG; Bhiman JN; Ismail A; Mahlangu B; Mohale T; Ntuli N; Scheepers C
EPI_ISL_4253672	Edendale Hosiptal	National Institute for Communicable Diseases of the National Health Laboratory Service	Amoako DG; Bhiman JN; Everatt J; Ismail A; Mahlangu B; Mnguni A; Mohale T; Ntuli N; Scheepers C; Sisonke Team
EPI_ISL_2086267	Enthabeni Lancet Laboratories	KRISP, KZN Research Innovation and Sequencing Platform	Emmanuel SJ; Giandhari J; Khan S; Lessells R; Mdlalose K; Naidoo Y; Pillay S; Ramphal U; Tegally H; Wilkinson E; York D; de Oliveira T
EPI_ISL_5416464	KIMBERLEY LABORATORY	National Institute for Communicable Diseases of the National Health Laboratory Service	Amoako DG; Bhiman JN; Everatt J; Ismail A; Mahlangu B; Mnguni A; Mohale T; Ntuli N; Scheepers C
EPI_ISL_660221, EPI_ISL_660222, EPI_ISL_660228, EPI_ISL_660229, EPI_ISL_660230, EPI_ISL_660231, EPI_ISL_660232, EPI_ISL_660233, EPI_ISL_660256, EPI_ISL_660257, EPI_ISL_660258, EPI_ISL_660664			
see above	KRISP, KZN Research Innovation and Sequencing Platform	KRISP, KZN Research Innovation and Sequencing Platform	Giandhari J; Khan S; Lessells R; Mdlalose K; Pillay S; Tegally H; Wilkinson E; York D; de Oliveira T
EPI_ISL_2688547, EPI_ISL_2688548	KRISP, KZn Research Innovation and Sequencing Platform	KRISP, KZn Research Innovation and Sequencing Platform	Giandhari Jennifer; Naidoo Yeshnee; Pillay Sureshnee; San James; Tegally Houriiyah; Tshabuila Derek; Wilkinson Eduan; Yajna Ramphal; de Oliveira Tulio
EPI_ISL_5918117	Kimberley Laboratory	National Institute for Communicable Diseases of the National Health Laboratory Service	Amoako DG; Bhiman JN; Everatt J; Ismail A; Mahlangu B; Mnguni A; Mohale T; Ntuli N; Scheepers C
EPI_ISL_2013037, EPI_ISL_2013040	Lancet Laboratories	National Institute for Communicable Diseases of the National Health Laboratory Service	Amoako DG; Bhiman JN; Glass A; Gottberg A; Mahlangu B; Mohale T; Ntuli N; Oliveira TD; Scheepers C; Tegally H; Viana R
EPI_ISL_736927, EPI_ISL_736930, EPI_ISL_736966, EPI_ISL_736967, EPI_ISL_736968, EPI_ISL_736995	MDS	KRISP, KZn Research Innovation and Sequencing Platform	ChimukangaraB; Giandhari J; Khan S; Lessells R; Mdlalose K; Pillay S; Tegally H; Wilkinson E; York D; de Oliveira T
EPI_ISL_660259, EPI_ISL_660260, EPI_ISL_660261, EPI_ISL_660262, EPI_ISL_660263	Molecualr Diagnostic Services (MDS)	KRISP, KZN Research Innovation and Sequencing Platform	Giandhari J; Khan S; Lessells R; Mdlalose K; Pillay S; Tegally H; Wilkinson E; York D; de Oliveira T
EPI_ISL_1250507, EPI_ISL_1250509, EPI_ISL_1250512, EPI_ISL_1250514, EPI_ISL_1250515, EPI_ISL_1250517, EPI_ISL_1250518, EPI_ISL_1250520, EPI_ISL_1250522, EPI_ISL_1250523, EPI_ISL_1250525, EPI_ISL_1250526, EPI_ISL_1250528, EPI_ISL_1250529, EPI_ISL_1250531, EPI_ISL_1250533, EPI_ISL_1250535, EPI_ISL_1250537, EPI_ISL_1250538, EPI_ISL_1250540, EPI_ISL_1250542, EPI_ISL_1250543, EPI_ISL_1250545, EPI_ISL_1250547, EPI_ISL_1250548, EPI_ISL_1250550, EPI_ISL_1250551, EPI_ISL_1250552			
see above	Molecular Diagnostics Services (MDS)	KRISP, KZN Research Innovation and Sequencing Platform	Emmanuel SJ; Giandhari J; Khan S; Lessells R; Maslo C; Mdlalose K; Pillay S; Sitharam L; Tegally H; Wilkinson E; York D; de Oliveira T
EPI_ISL_529720, EPI_ISL_529721, EPI_ISL_529722, EPI_ISL_529723, EPI_ISL_529724, EPI_ISL_529725, EPI_ISL_529726, EPI_ISL_529727, EPI_ISL_529728, EPI_ISL_529729, EPI_ISL_529730, EPI_ISL_529731, EPI_ISL_529732, EPI_ISL_529733, EPI_ISL_529734, EPI_ISL_529735, EPI_ISL_529736, EPI_ISL_529737, EPI_ISL_529738, EPI_ISL_529739, EPI_ISL_529740, EPI_ISL_529741, EPI_ISL_529742, EPI_ISL_529743, EPI_ISL_529744, EPI_ISL_529745, EPI_ISL_529746, EPI_ISL_529747, EPI_ISL_529748, EPI_ISL_529749, EPI_ISL_529750, EPI_ISL_529751, EPI_ISL_529752, EPI_ISL_529753, EPI_ISL_529754, EPI_ISL_529755, EPI_ISL_529756, EPI_ISL_529757, EPI_ISL_529758, EPI_ISL_529759, EPI_ISL_529760, EPI_ISL_529761, EPI_ISL_529762, EPI_ISL_529763, EPI_ISL_529764, EPI_ISL_529765, EPI_ISL_529766, EPI_ISL_529767, EPI_ISL_529768, EPI_ISL_529769, EPI_ISL_529770, EPI_ISL_529771, EPI_ISL_529772, EPI_ISL_529773, EPI_ISL_529774, EPI_ISL_529775, EPI_ISL_529776, EPI_ISL_529777, EPI_ISL_529778, EPI_ISL_529779, EPI_ISL_529780, EPI_ISL_529781, EPI_ISL_529782, EPI_ISL_529783, EPI_ISL_529784, EPI_ISL_529785, EPI_ISL_529786, EPI_ISL_529787, EPI_ISL_529788, EPI_ISL_529789, EPI_ISL_529790, EPI_ISL_529791, EPI_ISL_529792, EPI_ISL_529793, EPI_ISL_529794, EPI_ISL_529795, EPI_ISL_529796, EPI_ISL_529797, EPI_ISL_529798, EPI_ISL_529799, EPI_ISL_529800, EPI_ISL_529801, EPI_ISL_529802, EPI_ISL_529803, EPI_ISL_535390, EPI_ISL_535391, EPI_ISL_535392, EPI_ISL_535393, EPI_ISL_535394, EPI_ISL_535395, EPI_ISL_535396, EPI_ISL_535397, EPI_ISL_535398, EPI_ISL_535399, EPI_ISL_535400, EPI_ISL_535401, EPI_ISL_535402, EPI_ISL_535403, EPI_ISL_535404, EPI_ISL_535503, EPI_ISL_535504, EPI_ISL_535505, EPI_ISL_535508, EPI_ISL_535509, EPI_ISL_535510, EPI_ISL_535516, EPI_ISL_535517, EPI_ISL_535518, EPI_ISL_535520, EPI_ISL_535521, EPI_ISL_535523, EPI_ISL_535524, EPI_ISL_535525, EPI_ISL_535526, EPI_ISL_535527, EPI_ISL_535528, EPI_ISL_535529, EPI_ISL_535531, EPI_ISL_535533, EPI_ISL_535535, EPI_ISL_535538, EPI_ISL_535540, EPI_ISL_535542, EPI_ISL_535544, EPI_ISL_535549, EPI_ISL_535550, EPI_ISL_535551, EPI_ISL_602632, EPI_ISL_602633, EPI_ISL_602634, EPI_ISL_602635, EPI_ISL_602636, EPI_ISL_602637, EPI_ISL_602638, EPI_ISL_602639, EPI_ISL_602640, EPI_ISL_602641, EPI_ISL_602642, EPI_ISL_602643, EPI_ISL_602644, EPI_ISL_602645, EPI_ISL_602646, EPI_ISL_602647, EPI_ISL_602648, EPI_ISL_602649, EPI_ISL_602650, EPI_ISL_602651, EPI_ISL_602652, EPI_ISL_602653, EPI_ISL_602654, EPI_ISL_602655, EPI_ISL_602656, EPI_ISL_602657, EPI_ISL_602658, EPI_ISL_602659, EPI_ISL_602660, EPI_ISL_602661, EPI_ISL_602662, EPI_ISL_602663, EPI_ISL_602664, EPI_ISL_602665, EPI_ISL_602666, EPI_ISL_602667, EPI_ISL_602668, EPI_ISL_602669, EPI_ISL_602670, EPI_ISL_602671, EPI_ISL_602672, EPI_ISL_602673, EPI_ISL_602674, EPI_ISL_602675, EPI_ISL_602676, EPI_ISL_602677, EPI_ISL_602678, EPI_ISL_602679, EPI_ISL_602680, EPI_ISL_602681, EPI_ISL_602682, EPI_ISL_602683, EPI_ISL_602684, EPI_ISL_602685, EPI_ISL_602686, EPI_ISL_602687, EPI_ISL_602688, EPI_ISL_602689, EPI_ISL_602690, EPI_ISL_602691, EPI_ISL_602692, EPI_ISL_602693, EPI_ISL_602694, EPI_ISL_602695, EPI_ISL_602696, EPI_ISL_602697, EPI_ISL_602698, EPI_ISL_602699, EPI_ISL_602700, EPI_ISL_602701, EPI_ISL_602702, EPI_ISL_602703, EPI_ISL_602704, EPI_ISL_602705, EPI_ISL_602706, EPI_ISL_602707, EPI_ISL_602708, EPI_ISL_602709, EPI_ISL_602710, EPI_ISL_602711, EPI_ISL_602712, EPI_ISL_602713, EPI_ISL_602714, EPI_ISL_602715, EPI_ISL_602716, EPI_ISL_602717, EPI_ISL_602718, EPI_ISL_602719, EPI_ISL_602720, EPI_ISL_602721, EPI_ISL_602722, EPI_ISL_602723, EPI_ISL_602724, EPI_ISL_602725, EPI_ISL_602726, EPI_ISL_602727, EPI_ISL_602728, EPI_ISL_602729, EPI_ISL_602730, EPI_ISL_602731, EPI_ISL_602732, EPI_ISL_602733, EPI_ISL_602734, EPI_ISL_602735, EPI_ISL_602736, EPI_ISL_602737, EPI_ISL_602738, EPI_ISL_602739, EPI_ISL_602740, EPI_ISL_602741, EPI_ISL_602742, EPI_ISL_602743, EPI_ISL_602744, EPI_ISL_602745, EPI_ISL_602746, EPI_ISL_602747, EPI_ISL_602748, EPI_ISL_602749, EPI_ISL_602750, EPI_ISL_602751, EPI_ISL_602752, EPI_ISL_602753, EPI_ISL_602754, EPI_ISL_602755, EPI_ISL_602756, EPI_ISL_602757, EPI_ISL_602758, EPI_ISL_602759, EPI_ISL_602760, EPI_ISL_602761, EPI_ISL_602762, EPI_ISL_602763, EPI_ISL_602764, EPI_ISL_602765, EPI_ISL_602766, EPI_ISL_602767, EPI_ISL_602768, EPI_ISL_602769, EPI_ISL_602770, EPI_ISL_602771, EPI_ISL_602772, EPI_ISL_602773, EPI_ISL_602774, EPI_ISL_602775, EPI_ISL_602776, EPI_ISL_602777, EPI_ISL_602778, EPI_ISL_602779, EPI_ISL_602780, EPI_ISL_602781, EPI_ISL_602782, EPI_ISL_602783, EPI_ISL_602784, EPI_ISL_602785, EPI_ISL_602786, EPI_ISL_602787, EPI_ISL_602788, EPI_ISL_602789, EPI_ISL_602790, EPI_ISL_602791, EPI_ISL_602792, EPI_ISL_602793, EPI_ISL_602794, EPI_ISL_602795, EPI_ISL_602796, EPI_ISL_602797, EPI_ISL_602798, EPI_ISL_602799, EPI_ISL_602800, EPI_ISL_602801, EPI_ISL_602802, EPI_ISL_602803, EPI_ISL_602804, EPI_ISL_602805, EPI_ISL_602806, EPI_ISL_602807, EPI_ISL_602808, EPI_ISL_602809, EPI_ISL_602810, EPI_ISL_602811, EPI_ISL_602812, EPI_ISL_602813, EPI_ISL_602814, EPI_ISL_602815, EPI_ISL_602816, EPI_ISL_602817, EPI_ISL_602818, EPI_ISL_602819, EPI_ISL_602820, EPI_ISL_602821, EPI_ISL_602822, EPI_ISL_602823, EPI_ISL_602824, EPI_ISL_602825, EPI_ISL_602826, EPI_ISL_602827, EPI_ISL_602828, EPI_ISL_602829, EPI_ISL_602830, EPI_ISL_602831, EPI_ISL_602832, EPI_ISL_602833, EPI_ISL_602834, EPI_ISL_602835, EPI_ISL_602836, EPI_ISL_602837, EPI_ISL_602838, EPI_ISL_602839, EPI_ISL_602840, EPI_ISL_602841, EPI_ISL_602842, EPI_ISL_602843, EPI_ISL_602844, EPI_ISL_602845, EPI_ISL_602846, EPI_ISL_602847, EPI_ISL_602848, EPI_ISL_602849, EPI_ISL_602850, EPI_ISL_602851, EPI_ISL_602852, EPI_ISL_602853, EPI_ISL_602854, EPI_ISL_602855, EPI_ISL_602856, EPI_ISL_602857, EPI_ISL_602858, EPI_ISL_602859, EPI_ISL_602860, EPI_ISL_602861, EPI_ISL_602862, EPI_ISL_602863, EPI_ISL_602864, EPI_ISL_602865, EPI_ISL_602866, EPI_ISL_602867, EPI_ISL_602868, EPI_ISL_602869, EPI_ISL_602870, EPI_ISL_602871, EPI_ISL_602872, EPI_ISL_602873, EPI_ISL_602874, EPI_ISL_602875, EPI_ISL_602876, EPI_ISL_602877, EPI_ISL_602878, EPI_ISL_602879, EPI_ISL_602880, EPI_ISL_602881, EPI_ISL_602882, EPI_ISL_602883, EPI_ISL_602884, EPI_ISL_602885, EPI_ISL_602886, EPI_ISL_602887, EPI_ISL_602888, EPI_ISL_602889, EPI_ISL_602890, EPI_ISL_602891, EPI_ISL_602892, EPI_ISL_602893, EPI_ISL_602894, EPI_ISL_602895, EPI_ISL_602896, EPI_ISL_602897, EPI_ISL_602898, EPI_ISL_602899, EPI_ISL_602900, EPI_ISL_602901, EPI_ISL_602902, EPI_ISL_602903, EPI_ISL_602904, EPI_ISL_602905, EPI_ISL_602906, EPI_ISL_602907, EPI_ISL_602908, EPI_ISL_602909, EPI_ISL_602910, EPI_ISL_602911, EPI_ISL_602912, EPI_ISL_602913, EPI_ISL_602914, EPI_ISL_602915, EPI_ISL_602916, EPI_ISL_602917, EPI_ISL_602918, EPI_ISL_602919, EPI_ISL_602920, EPI_ISL_602921, EPI_ISL_602922, EPI_ISL_602923, EPI_ISL_602924, EPI_ISL_602925, EPI_ISL_602926, EPI_ISL_602927, EPI_ISL_602928, EPI_ISL_602929, EPI_ISL_602930, EPI_ISL_605785, EPI_ISL_605786, EPI_ISL_605787, EPI_ISL_605788, EPI_ISL_605789, EPI_ISL_605790, EPI_ISL_660159, EPI_ISL_660160, EPI_ISL_660161, EPI_ISL_660162, EPI_ISL_660163, EPI_ISL_660164, EPI_ISL_660177, EPI_ISL_660178, EPI_ISL_660179, EPI_ISL_660180, EPI_ISL_660181, EPI_ISL_660182, EPI_ISL_660183, EPI_ISL_660184, EPI_ISL_660185, EPI_ISL_660186, EPI_ISL_660187, EPI_ISL_660188, EPI_ISL_660189, EPI_ISL_660190, EPI_ISL_660191, EPI_ISL_660192, EPI_ISL_660193, EPI_ISL_660194, EPI_ISL_660195, EPI_ISL_660196, EPI_ISL_660197, EPI_ISL_660198, EPI_ISL_660199, EPI_ISL_660200, EPI_ISL_660201, EPI_ISL_660202, EPI_ISL_660203, EPI_ISL_660204, EPI_ISL_660205, EPI_ISL_660206, EPI_ISL_660207, EPI_ISL_660208, EPI_ISL_660209, EPI_ISL_660210, EPI_ISL_660211, EPI_ISL_660212, EPI_ISL_660213, EPI_ISL_660214, EPI_ISL_660215, EPI_ISL_660216, EPI_ISL_660217, EPI_ISL_660218, EPI_ISL_660219, EPI_ISL_660220, EPI_ISL_660223, EPI_ISL_660224, EPI_ISL_660225, EPI_ISL_660226, EPI_ISL_660227, EPI_ISL_660234, EPI_ISL_660235, EPI_ISL_660236, EPI_ISL_660237, EPI_ISL_660238, EPI_ISL_660239, EPI_ISL_660240, EPI_ISL_660241, EPI_ISL_660242, EPI_ISL_660243, EPI_ISL_660244, EPI_ISL_660245, EPI_ISL_660246, EPI_ISL_660247, EPI_ISL_660248, EPI_ISL_660249, EPI_ISL_660250, EPI_ISL_660251, EPI_ISL_660252, EPI_ISL_660253, EPI_ISL_660254, EPI_ISL_660255, EPI_ISL_660616, EPI_ISL_660617, EPI_ISL_660618, EPI_ISL_660619, EPI_ISL_660620, EPI_ISL_660621, EPI_ISL_660622, EPI_ISL_660623, EPI_ISL_660624, EPI_ISL_660625, EPI_ISL_660626, EPI_ISL_660627, EPI_ISL_660628, EPI_ISL_660629, EPI_ISL_660630, EPI_ISL_660631, EPI_ISL_660632, EPI_ISL_660633, EPI_ISL_660634, EPI_ISL_660635, EPI_ISL_660636, EPI_ISL_660637, EPI_ISL_660638, EPI_ISL_660639, EPI_ISL_660640, EPI_ISL_660641, EPI_ISL_660642, EPI_ISL_660643, EPI_ISL_660644, EPI_ISL_660645, EPI_ISL_660646, EPI_ISL_660647, EPI_ISL_660648, EPI_ISL_660649, EPI_ISL_660650, EPI_ISL_660651, EPI_ISL_660652, EPI_ISL_660653, EPI_ISL_660654, EPI_ISL_660655, EPI_ISL_660656, EPI_ISL_660657, EPI_ISL_660658, EPI_ISL_660659, EPI_ISL_660660, EPI_ISL_660661, EPI_ISL_660662, EPI_ISL_660663, EPI_ISL_678570, EPI_ISL_678598, EPI_ISL_678599, EPI_ISL_678600, EPI_ISL_678601, EPI_ISL_678602, EPI_ISL_678603, EPI_ISL_678604, EPI_ISL_678605, EPI_ISL_678606, EPI_ISL_678607, EPI_ISL_678608, EPI_ISL_678609, EPI_ISL_678610, EPI_ISL_678611, EPI_ISL_678612, EPI_ISL_678613, EPI_ISL_678614, EPI_ISL_678615, EPI_ISL_678616, EPI_ISL_678617, EPI_ISL_678618, EPI_ISL_678619, EPI_ISL_678620, EPI_ISL_678621, EPI_ISL_678622, EPI_ISL_678623, EPI_ISL_678624, EPI_ISL_678643, EPI_ISL_736925, EPI_ISL_736926, EPI_ISL_736931, EPI_ISL_736932, EPI_ISL_736933, EPI_ISL_736934, EPI_ISL_736935, EPI_ISL_736940, EPI_ISL_736941, EPI_ISL_736942, EPI_ISL_736943, EPI_ISL_736944, EPI_ISL_736945, EPI_ISL_736946, EPI_ISL_736947, EPI_ISL_736948, EPI_ISL_736949, EPI_ISL_736950, EPI_ISL_736951, EPI_ISL_736952, EPI_ISL_736953, EPI_ISL_736954, EPI_ISL_736955, EPI_ISL_736956, EPI_ISL_736957, EPI_ISL_736958, EPI_ISL_736959, EPI_ISL_736960, EPI_ISL_736984, EPI_ISL_736985, EPI_ISL_736986, EPI_ISL_736987, EPI_ISL_736988, EPI_ISL_736989, EPI_ISL_736990, EPI_ISL_736991, EPI_ISL_736992, EPI_ISL_736993, EPI_ISL_736994, EPI_ISL_825128, EPI_ISL_825129, EPI_ISL_825130, EPI_ISL_825131, EPI_ISL_825132, EPI_ISL_825133, EPI_ISL_825134, EPI_ISL_825135, EPI_ISL_825136, EPI_ISL_825137, EPI_ISL_825138, EPI_ISL_825139, EPI_ISL_825438, EPI_ISL_825439, EPI_ISL_825440, EPI_ISL_825441, EPI_ISL_825442, EPI_ISL_825443, EPI_ISL_825444, EPI_ISL_825445, EPI_ISL_825446, EPI_ISL_825447, EPI_ISL_825448, EPI_ISL_825449, EPI_ISL_825450, EPI_ISL_825451, EPI_ISL_825452, EPI_ISL_825453, EPI_ISL_825454, EPI_ISL_825455, EPI_ISL_825456, EPI_ISL_825457, EPI_ISL_825458, EPI_ISL_825459, EPI_ISL_825460, EPI_ISL_825461, EPI_ISL_825462, EPI_ISL_825463, EPI_ISL_825464, EPI_ISL_825465, EPI_ISL_825466, EPI_ISL_825467, EPI_ISL_825468, EPI_ISL_825469, EPI_ISL_825470, EPI_ISL_825471, EPI_ISL_825472, EPI_ISL_825473, EPI_ISL_825474, EPI_ISL_825475, EPI_ISL_825476, EPI_ISL_825477, EPI_ISL_825478, EPI_ISL_825479, EPI_ISL_825480, EPI_ISL_825481, EPI_ISL_825482, EPI_ISL_825483, EPI_ISL_825484, EPI_ISL_825485, EPI_ISL_825486, EPI_ISL_825487, EPI_ISL_825488, EPI_ISL_825489			
see above	NHLS-IALCH	KRISP, KZN Research Innovation and Sequencing Platform	Cele S; ChimukangaraB; Gazy I; Giandhari J; Karim F; Khan S; Lessells R; Mdlalose K; Pillay S; Sigal A; Tegally H; Wilkinson E; York D; de Oliveira T
EPI_ISL_860554, EPI_ISL_860555, EPI_ISL_860556, EPI_ISL_860557, EPI_ISL_860558, EPI_ISL_860559, EPI_ISL_860560, EPI_ISL_860561, EPI_ISL_860562, EPI_ISL_860563, EPI_ISL_860564, EPI_ISL_860565, EPI_ISL_860566, EPI_ISL_860567, EPI_ISL_860568, EPI_ISL_860569, EPI_ISL_860570, EPI_ISL_860571, EPI_ISL_860572, EPI_ISL_860573, EPI_ISL_860574, EPI_ISL_860575, EPI_ISL_860576, EPI_ISL_860577, EPI_ISL_860578, EPI_ISL_860579, EPI_ISL_860580, EPI_ISL_860581, EPI_ISL_860582, EPI_ISL_860583, EPI_ISL_860584, EPI_ISL_860585, EPI_ISL_860586, EPI_ISL_860587, EPI_ISL_860588, EPI_ISL_860589, EPI_ISL_860590, EPI_ISL_860591, EPI_ISL_860592, EPI_ISL_860593, EPI_ISL_860594, EPI_ISL_860595, EPI_ISL_860596, EPI_ISL_860597, EPI_ISL_860598, EPI_ISL_860599, EPI_ISL_860600, EPI_ISL_860601, EPI_ISL_860602, EPI_ISL_860603, EPI_ISL_860604, EPI_ISL_860605, EPI_ISL_860606, EPI_ISL_860607, EPI_ISL_860608, EPI_ISL_860609, EPI_ISL_860610, EPI_ISL_860611, EPI_ISL_860612, EPI_ISL_860613, EPI_ISL_860614, EPI_ISL_860615, EPI_ISL_860616, EPI_ISL_860617, EPI_ISL_860618, EPI_ISL_860619, EPI_ISL_860620, EPI_ISL_860621, EPI_ISL_860622, EPI_ISL_860623, EPI_ISL_860624, EPI_ISL_860625,			

EPI_ISL_860626, EPI_ISL_860627, EPI_ISL_860628, EPI_ISL_860629, EPI_ISL_860630			
see above	NHLS-IALCH	KRISP, KZn Research Innovation and Sequencing Platform	Giandhari J; Khan S; Lessells R; Mdlalose K; Pillay S; Tegally H; Wilkinson E; York D; de Oliveira T
EPI_ISL_3447709, EPI_ISL_3447710, EPI_ISL_3447711, EPI_ISL_3447716, EPI_ISL_3447717, EPI_ISL_3447719, EPI_ISL_3447720, EPI_ISL_3447721, EPI_ISL_3447722, EPI_ISL_3447723, EPI_ISL_3447724, EPI_ISL_3447725, EPI_ISL_3447726, EPI_ISL_3447727, EPI_ISL_3447730, EPI_ISL_3447731, EPI_ISL_3447732, EPI_ISL_3447733, EPI_ISL_3447735, EPI_ISL_3447736, EPI_ISL_3447740, EPI_ISL_3447741, EPI_ISL_3447742, EPI_ISL_3447745, EPI_ISL_3447747, EPI_ISL_3447749, EPI_ISL_3447750, EPI_ISL_3447751, EPI_ISL_3447753, EPI_ISL_3447755, EPI_ISL_3447756, EPI_ISL_3447757, EPI_ISL_3447758, EPI_ISL_3447759, EPI_ISL_3447782, EPI_ISL_3447797, EPI_ISL_3447801, EPI_ISL_3447805, EPI_ISL_3447810, EPI_ISL_3447811, EPI_ISL_3447813, EPI_ISL_3447817, EPI_ISL_3447819, EPI_ISL_3447822, EPI_ISL_3447824, EPI_ISL_3447826, EPI_ISL_3447827, EPI_ISL_3447828, EPI_ISL_3447829, EPI_ISL_3447830, EPI_ISL_3447831, EPI_ISL_3447832, EPI_ISL_3447833, EPI_ISL_3447834, EPI_ISL_3447835, EPI_ISL_3447836, EPI_ISL_3447838, EPI_ISL_3447839, EPI_ISL_3447840, EPI_ISL_3447841, EPI_ISL_3447842, EPI_ISL_3447855, EPI_ISL_3447869, EPI_ISL_3447870, EPI_ISL_3447871, EPI_ISL_3447872, EPI_ISL_3447873, EPI_ISL_3447874, EPI_ISL_3447875, EPI_ISL_3447876, EPI_ISL_3447877, EPI_ISL_3447878, EPI_ISL_3447879, EPI_ISL_3447880			
see above	NHLS_IALCH	KRISP, KZN Research Innovation and Sequencing Platform	Giandhari Jennifer; Naidoo Yeshnee; Pillay Sureshnee; San James; Tegally Houriiyah; Tshabuila Derek; Tshiabuila Derek; Wilkinson Eduan; Yajna Ramphal; de Oliveira Tulio
EPI_ISL_4473168, EPI_ISL_4473194, EPI_ISL_4473195, EPI_ISL_4473198, EPI_ISL_4473201	NHLS_VIRO	CERI, Centre for Epidemic Response and Innvoation, Stellenbosch University and KRISP, KZN Research Innovation and Sequencing Platform, UKZN.	Giandhari Jennifer; Naidoo Yeshnee; Pillay Sureshnee; San James; Tegally Houriiyah; Tshiabuila Derek; Wilkinson Eduan; Yajna Ramphal; de Oliveira Tulio
EPI_ISL_2693036, EPI_ISL_2693037, EPI_ISL_2693038, EPI_ISL_2693039, EPI_ISL_2693040, EPI_ISL_2693041, EPI_ISL_2693042, EPI_ISL_2693043, EPI_ISL_2693044, EPI_ISL_2693045, EPI_ISL_2693046, EPI_ISL_2693047, EPI_ISL_2693048, EPI_ISL_2693049, EPI_ISL_2693050, EPI_ISL_2693051, EPI_ISL_2693052, EPI_ISL_2693053, EPI_ISL_2693054, EPI_ISL_2693055, EPI_ISL_2693056, EPI_ISL_2693057, EPI_ISL_2693058, EPI_ISL_2693059, EPI_ISL_2693060, EPI_ISL_2693061, EPI_ISL_2693063, EPI_ISL_2693064, EPI_ISL_2693065, EPI_ISL_2693066, EPI_ISL_2693067, EPI_ISL_2693068, EPI_ISL_2693069, EPI_ISL_2693070, EPI_ISL_2693071, EPI_ISL_2693072, EPI_ISL_2693073, EPI_ISL_2693074, EPI_ISL_2693075, EPI_ISL_2693076, EPI_ISL_2693077, EPI_ISL_2693078, EPI_ISL_2693079, EPI_ISL_2693080, EPI_ISL_2693081, EPI_ISL_2693082, EPI_ISL_2693083, EPI_ISL_2693084, EPI_ISL_2693085, EPI_ISL_2693086, EPI_ISL_2693087, EPI_ISL_2693089, EPI_ISL_2693090, EPI_ISL_2693091, EPI_ISL_2693092, EPI_ISL_2693093, EPI_ISL_2693094, EPI_ISL_2693095, EPI_ISL_3161759, EPI_ISL_3161760, EPI_ISL_3161761, EPI_ISL_3161762, EPI_ISL_3161763, EPI_ISL_3161764, EPI_ISL_3161765, EPI_ISL_3161766, EPI_ISL_3161767, EPI_ISL_3161768, EPI_ISL_3161769, EPI_ISL_3161770, EPI_ISL_3161771, EPI_ISL_3161772, EPI_ISL_3161773, EPI_ISL_3161774, EPI_ISL_3161775, EPI_ISL_3161776, EPI_ISL_3161777, EPI_ISL_3161778, EPI_ISL_3161779, EPI_ISL_3161780, EPI_ISL_3161781, EPI_ISL_3161782, EPI_ISL_3161783, EPI_ISL_3161784, EPI_ISL_3161785, EPI_ISL_3161786, EPI_ISL_3161787, EPI_ISL_3161788, EPI_ISL_3161789, EPI_ISL_3161790, EPI_ISL_3161791, EPI_ISL_3161792, EPI_ISL_3161793, EPI_ISL_3161794, EPI_ISL_3161795, EPI_ISL_3161796, EPI_ISL_3161797, EPI_ISL_3161798, EPI_ISL_3161799, EPI_ISL_3161800, EPI_ISL_3161801, EPI_ISL_3261864, EPI_ISL_3261865, EPI_ISL_3261866, EPI_ISL_3261867, EPI_ISL_3261868, EPI_ISL_3261869, EPI_ISL_3261870, EPI_ISL_3261871, EPI_ISL_3261872, EPI_ISL_3261873, EPI_ISL_3261874, EPI_ISL_3261875, EPI_ISL_3261876, EPI_ISL_3261877, EPI_ISL_3261878, EPI_ISL_3261879, EPI_ISL_3261880, EPI_ISL_3261881, EPI_ISL_3261882, EPI_ISL_3261883, EPI_ISL_3261884, EPI_ISL_3261885, EPI_ISL_3261886, EPI_ISL_3261887, EPI_ISL_3261888, EPI_ISL_3261889, EPI_ISL_3261890, EPI_ISL_3261891, EPI_ISL_3261892, EPI_ISL_3261893, EPI_ISL_3261894, EPI_ISL_3261895, EPI_ISL_3261896, EPI_ISL_3261897, EPI_ISL_3261898, EPI_ISL_3261899, EPI_ISL_3261900, EPI_ISL_3261901, EPI_ISL_3261902, EPI_ISL_3261903, EPI_ISL_3261904, EPI_ISL_3261905, EPI_ISL_3261924, EPI_ISL_3261925, EPI_ISL_3261926, EPI_ISL_3261927, EPI_ISL_3261928, EPI_ISL_3261929, EPI_ISL_3261930, EPI_ISL_3261931, EPI_ISL_3261932, EPI_ISL_3261933, EPI_ISL_3261934, EPI_ISL_3261935, EPI_ISL_3261936, EPI_ISL_3261937, EPI_ISL_3261938, EPI_ISL_3261939, EPI_ISL_3261940, EPI_ISL_3261941, EPI_ISL_3261942, EPI_ISL_3261943, EPI_ISL_3261944, EPI_ISL_3261945, EPI_ISL_3261946, EPI_ISL_3261947, EPI_ISL_3261948, EPI_ISL_3261949, EPI_ISL_3261950, EPI_ISL_3261951, EPI_ISL_3261952, EPI_ISL_3261953, EPI_ISL_3261954, EPI_ISL_3261955, EPI_ISL_3261956, EPI_ISL_3261957, EPI_ISL_3261958, EPI_ISL_3261959, EPI_ISL_3261960, EPI_ISL_3261961, EPI_ISL_3261962, EPI_ISL_3261963, EPI_ISL_3261964, EPI_ISL_3261965, EPI_ISL_3261966, EPI_ISL_3261967, EPI_ISL_3261968, EPI_ISL_3261969, EPI_ISL_3261970, EPI_ISL_3261971, EPI_ISL_3261972, EPI_ISL_3261973, EPI_ISL_3261974, EPI_ISL_3261975, EPI_ISL_3261976, EPI_ISL_3261977, EPI_ISL_3261978, EPI_ISL_3261979, EPI_ISL_3261980, EPI_ISL_3261981, EPI_ISL_3261982, EPI_ISL_3261983			
see above	NHLS_VIRO	KRISP, KZN Research Innovation and Sequencing Platform	Giandhari Jennifer; Naidoo Yeshnee; Pillay Sureshnee; San James; Tegally Houriiyah; Tshabuila Derek; Wilkinson Eduan; Yajna Ramphal; de Oliveira Tulio
EPI_ISL_2494363, EPI_ISL_2494368, EPI_ISL_2494376, EPI_ISL_2494387, EPI_ISL_2494390, EPI_ISL_2494397, EPI_ISL_2494403, EPI_ISL_2494417, EPI_ISL_2494421, EPI_ISL_2494422, EPI_ISL_2494424, EPI_ISL_2494427, EPI_ISL_2494428, EPI_ISL_2494431, EPI_ISL_2494437, EPI_ISL_2494440, EPI_ISL_2494441, EPI_ISL_2494445, EPI_ISL_2494446, EPI_ISL_2494447, EPI_ISL_2494449, EPI_ISL_2494450, EPI_ISL_2494451, EPI_ISL_2494453, EPI_ISL_2494454, EPI_ISL_2494455, EPI_ISL_2494457, EPI_ISL_2494458, EPI_ISL_2494459, EPI_ISL_2494462, EPI_ISL_2494463, EPI_ISL_2494464, EPI_ISL_2494465, EPI_ISL_2494469, EPI_ISL_2494470, EPI_ISL_2494474, EPI_ISL_2494475, EPI_ISL_2494476, EPI_ISL_2494480, EPI_ISL_2494490, EPI_ISL_2494491, EPI_ISL_2494492, EPI_ISL_2494493, EPI_ISL_2494499, EPI_ISL_2494500, EPI_ISL_2494502, EPI_ISL_2494503, EPI_ISL_2494504, EPI_ISL_2494505, EPI_ISL_2494506, EPI_ISL_2494507, EPI_ISL_2494508, EPI_ISL_2494509, EPI_ISL_2494510, EPI_ISL_2494511, EPI_ISL_2494514, EPI_ISL_2494515, EPI_ISL_2494516, EPI_ISL_2494517, EPI_ISL_2494523, EPI_ISL_2494525, EPI_ISL_2494531, EPI_ISL_2494532, EPI_ISL_2494536, EPI_ISL_2494541, EPI_ISL_2494542, EPI_ISL_2494555, EPI_ISL_2494556, EPI_ISL_2494569, EPI_ISL_2494579, EPI_ISL_2494606, EPI_ISL_2494607, EPI_ISL_2494608, EPI_ISL_2494611, EPI_ISL_2494612, EPI_ISL_2494613, EPI_ISL_2494615, EPI_ISL_2494617, EPI_ISL_2494618, EPI_ISL_2494619, EPI_ISL_2494620, EPI_ISL_2494621, EPI_ISL_2494622, EPI_ISL_2494623, EPI_ISL_2494624, EPI_ISL_2494625, EPI_ISL_2494626, EPI_ISL_2727380, EPI_ISL_2727381, EPI_ISL_2727382, EPI_ISL_2727383, EPI_ISL_2727384, EPI_ISL_2727385, EPI_ISL_2727386, EPI_ISL_2727387, EPI_ISL_2727388, EPI_ISL_2727389, EPI_ISL_2727390, EPI_ISL_2727391, EPI_ISL_2727392, EPI_ISL_2727393, EPI_ISL_2727394, EPI_ISL_2727395, EPI_ISL_2727396, EPI_ISL_2727397, EPI_ISL_2727398, EPI_ISL_2727399, EPI_ISL_2727400, EPI_ISL_2727401, EPI_ISL_2727402, EPI_ISL_2727403			
see above	NHLS_VIRO	KRISP, KZn Research Innovation and Sequencing Platform	Baillie Vicky; Giandhari Jennifer; Madhi Shabir; Naidoo Yeshnee; Pillay Sureshnee; San James; Tegally Houriiyah; Tshabuila Derek; Wilkinson Eduan; Yajna Ramphal; de Oliveira Tulio; du Plessis Jeanine
EPI_ISL_634978, EPI_ISL_634979, EPI_ISL_634980, EPI_ISL_634981, EPI_ISL_634982, EPI_ISL_634983, EPI_ISL_634984, EPI_ISL_634985, EPI_ISL_634986, EPI_ISL_634987, EPI_ISL_634988, EPI_ISL_634989, EPI_ISL_634990, EPI_ISL_634991, EPI_ISL_634992, EPI_ISL_634993, EPI_ISL_634994, EPI_ISL_634995, EPI_ISL_634996, EPI_ISL_634997, EPI_ISL_634998, EPI_ISL_634999, EPI_ISL_635000, EPI_ISL_635001, EPI_ISL_635002, EPI_ISL_635003, EPI_ISL_635004, EPI_ISL_635005, EPI_ISL_635006, EPI_ISL_635007, EPI_ISL_635008, EPI_ISL_635009, EPI_ISL_635010, EPI_ISL_635011, EPI_ISL_635012, EPI_ISL_635013, EPI_ISL_635014, EPI_ISL_635015, EPI_ISL_635016, EPI_ISL_635017, EPI_ISL_635018, EPI_ISL_635019, EPI_ISL_635020, EPI_ISL_635021, EPI_ISL_635022, EPI_ISL_635023, EPI_ISL_635024, EPI_ISL_635025, EPI_ISL_635026, EPI_ISL_635027, EPI_ISL_635028, EPI_ISL_635029, EPI_ISL_635030, EPI_ISL_635031, EPI_ISL_635032, EPI_ISL_635033, EPI_ISL_635034, EPI_ISL_635035, EPI_ISL_635036, EPI_ISL_635037, EPI_ISL_635038, EPI_ISL_635039, EPI_ISL_635040, EPI_ISL_635041, EPI_ISL_635042, EPI_ISL_635043, EPI_ISL_635044, EPI_ISL_635045, EPI_ISL_635046, EPI_ISL_635047, EPI_ISL_635048, EPI_ISL_635049, EPI_ISL_635050, EPI_ISL_635051, EPI_ISL_635052, EPI_ISL_635053, EPI_ISL_635054, EPI_ISL_635055, EPI_ISL_635056, EPI_ISL_635057, EPI_ISL_635058			
see above	National Health Laboratory Service - Inkosi Albert Luthuli Central Hospital (NHLS-IALCH)	KRISP, KZN Research Innovation and Sequencing Platform	Giandhari J; Khan S; Lessells R; Mdlalose K; Pillay S; Tegally H; Wilkinson E; York D; de Oliveira T
EPI_ISL_4505703	National Health Laboratory Service, Eastern Cape, South Africa	KRISP, KZN Research Innovation and Sequencing Platform	Giandhari Jennifer; Naidoo Yeshnee; Oluwakemi Laguda-Akingba; Pillay Sureshnee; San James; Tegally Houriiyah; Tshiabuila Derek; Wilkinson Eduan; Yajna Ramphal; de Oliveira Tulio
EPI_ISL_4505635, EPI_ISL_4505636, EPI_ISL_4505637, EPI_ISL_4505638, EPI_ISL_4505639, EPI_ISL_4505640, EPI_ISL_4505641, EPI_ISL_4505642, EPI_ISL_4505643, EPI_ISL_4505644, EPI_ISL_4505645, EPI_ISL_4505646, EPI_ISL_4505647, EPI_ISL_4505648, EPI_ISL_4505649, EPI_ISL_4505650, EPI_ISL_4505651, EPI_ISL_4505652, EPI_ISL_4505653, EPI_ISL_4505654, EPI_ISL_4505655, EPI_ISL_4505656, EPI_ISL_4505657, EPI_ISL_4505658, EPI_ISL_4505659, EPI_ISL_4505660, EPI_ISL_4505661, EPI_ISL_4505662, EPI_ISL_4505663, EPI_ISL_4505664, EPI_ISL_4505665, EPI_ISL_4505666, EPI_ISL_4505667, EPI_ISL_4505668, EPI_ISL_4505669, EPI_ISL_4505670, EPI_ISL_4505671			
see above	National Health Laboratory Service, Kwazulu Natal, South Africa	KRISP, KZN Research Innovation and Sequencing Platform	Giandhari Jennifer; Naidoo Yeshnee; Nokukhanya Mdlalose; Pillay Sureshnee; San James; Tegally Houriiyah; Tshiabuila Derek; Wilkinson Eduan; Yajna Ramphal; de Oliveira Tulio
EPI_ISL_4876926, EPI_ISL_4876934, EPI_ISL_4876935, EPI_ISL_4876943, EPI_ISL_4876949, EPI_ISL_4876957, EPI_ISL_4876958, EPI_ISL_4876967, EPI_ISL_4876976, EPI_ISL_4876983, EPI_ISL_4876984, EPI_ISL_4876989, EPI_ISL_4876997, EPI_ISL_4876998, EPI_ISL_4877005, EPI_ISL_4877009, EPI_ISL_4877015, EPI_ISL_4877022, EPI_ISL_4877025, EPI_ISL_4877026, EPI_ISL_4877033, EPI_ISL_4877043, EPI_ISL_4877046, EPI_ISL_4877047, EPI_ISL_4877053, EPI_ISL_4877058, EPI_ISL_5099156, EPI_ISL_5099157, EPI_ISL_5099158, EPI_ISL_5099217, EPI_ISL_5942679, EPI_ISL_5942680, EPI_ISL_5942681, EPI_ISL_5942682, EPI_ISL_5942688, EPI_ISL_5942689, EPI_ISL_5942690, EPI_ISL_5942691, EPI_ISL_5942692, EPI_ISL_5942693, EPI_ISL_5942694, EPI_ISL_5942701, EPI_ISL_5942702, EPI_ISL_5942703, EPI_ISL_5942717, EPI_ISL_5942723, EPI_ISL_5942730, EPI_ISL_5942734, EPI_ISL_5942742, EPI_ISL_5942749, EPI_ISL_5942757, EPI_ISL_5942768, EPI_ISL_5942777, EPI_ISL_5942783, EPI_ISL_5942790, EPI_ISL_5942795, EPI_ISL_5942796, EPI_ISL_5942804, EPI_ISL_5942810, EPI_ISL_5942821, EPI_ISL_5942835, EPI_ISL_5942836, EPI_ISL_5942843, EPI_ISL_6422290, EPI_ISL_6422291, EPI_ISL_6422292, EPI_ISL_6422293, EPI_ISL_6422294, EPI_ISL_6422295, EPI_ISL_6422296, EPI_ISL_6422297, EPI_ISL_6422298, EPI_ISL_6422299, EPI_ISL_6422300, EPI_ISL_6422301, EPI_ISL_6422302, EPI_ISL_6422303, EPI_ISL_6422304, EPI_ISL_6585201, EPI_ISL_6585202, EPI_ISL_6585203, EPI_ISL_6585204, EPI_ISL_6585205, EPI_ISL_6585206, EPI_ISL_6585207, EPI_ISL_6585208, EPI_ISL_6585209, EPI_ISL_6585210, EPI_ISL_6585211, EPI_ISL_6585212, EPI_ISL_6585213, EPI_ISL_6585214, EPI_ISL_6585215, EPI_ISL_6585216, EPI_ISL_6585217, EPI_ISL_6585223, EPI_ISL_6585224, EPI_ISL_6585225, EPI_ISL_6585226, EPI_ISL_6585227, EPI_ISL_6585228, EPI_ISL_6585229, EPI_ISL_6699699, EPI_ISL_6699700, EPI_ISL_6699701, EPI_ISL_6699702, EPI_ISL_6699703, EPI_ISL_6699704, EPI_ISL_6699705, EPI_ISL_6699706, EPI_ISL_6699707, EPI_ISL_6699708, EPI_ISL_6699709, EPI_ISL_6699710, EPI_ISL_6699711, EPI_ISL_6699712, EPI_ISL_6699713, EPI_ISL_6699714, EPI_ISL_6699715, EPI_ISL_6699716, EPI_ISL_6699717, EPI_ISL_6699718, EPI_ISL_6699719, EPI_ISL_6699720, EPI_ISL_6699721, EPI_ISL_6699722, EPI_ISL_6699723, EPI_ISL_6699724, EPI_ISL_6699725, EPI_ISL_6699726, EPI_ISL_6699727, EPI_ISL_6795195, EPI_ISL_6795196, EPI_ISL_6795197, EPI_ISL_6795198, EPI_ISL_6795199, EPI_ISL_6795200, EPI_ISL_6795201, EPI_ISL_6795202, EPI_ISL_6795203, EPI_ISL_6795204, EPI_ISL_6795406, EPI_ISL_7015209, EPI_ISL_7015210, EPI_ISL_7015211, EPI_ISL_7015212, EPI_ISL_7015213, EPI_ISL_7015214, EPI_ISL_7015215, EPI_ISL_7015216, EPI_ISL_7015217, EPI_ISL_7015218, EPI_ISL_7015219, EPI_ISL_7015220, EPI_ISL_7015221, EPI_ISL_7015222, EPI_ISL_7015223, EPI_ISL_7015224, EPI_ISL_7015225, EPI_ISL_7015226, EPI_ISL_7015227, EPI_ISL_7015228, EPI_ISL_7015229, EPI_ISL_7015230, EPI_ISL_7015231, EPI_ISL_7015232, EPI_ISL_7015233, EPI_ISL_7310589, EPI_ISL_7310595, EPI_ISL_7310605, EPI_ISL_7310613, EPI_ISL_7310622, EPI_ISL_7310630, EPI_ISL_7310631, EPI_ISL_7310636, EPI_ISL_7310643, EPI_ISL_7310648, EPI_ISL_7310658, EPI_ISL_7310666, EPI_ISL_7310675, EPI_ISL_7310679, EPI_ISL_7310685, EPI_ISL_7310694, EPI_ISL_7310703, EPI_ISL_7310710, EPI_ISL_7310719, EPI_ISL_7310725, EPI_ISL_7310733, EPI_ISL_7310742, EPI_ISL_7310747, EPI_ISL_7358050, EPI_ISL_7358051, EPI_ISL_7358052, EPI_ISL_7358053, EPI_ISL_7358054, EPI_ISL_7358055, EPI_ISL_7358056, EPI_ISL_7358057, EPI_ISL_7358058, EPI_ISL_7358059, EPI_ISL_7358064, EPI_ISL_7358065, EPI_ISL_7358066, EPI_ISL_7358067, EPI_ISL_7358068, EPI_ISL_7358069, EPI_ISL_7358070, EPI_ISL_7358071, EPI_ISL_7358072, EPI_ISL_7358073, EPI_ISL_7358074, EPI_ISL_7358075, EPI_ISL_7358076, EPI_ISL_7358077, EPI_ISL_7358078, EPI_ISL_7358079, EPI_ISL_7358080, EPI_ISL_7358081, EPI_ISL_7358082, EPI_ISL_7358083, EPI_ISL_7358084, EPI_ISL_7358085, EPI_ISL_7358089, EPI_ISL_7358090, EPI_ISL_7358091, EPI_ISL_7358092, EPI_ISL_7358093, EPI_ISL_7381192, EPI_ISL_7381193, EPI_ISL_7381194, EPI_ISL_7381195, EPI_ISL_7381196, EPI_ISL_7381197, EPI_ISL_7381198, EPI_ISL_7381199, EPI_ISL_7381200, EPI_ISL_7381201, EPI_ISL_7381202, EPI_ISL_7381203, EPI_ISL_7381204, EPI_ISL_7381205, EPI_ISL_7381206, EPI_ISL_7381207, EPI_ISL_7417008, EPI_ISL_7417082, EPI_ISL_7417091, EPI_ISL_7417102, EPI_ISL_7417181, EPI_ISL_7417197, EPI_ISL_7417232, EPI_ISL_7417255, EPI_ISL_7417393, EPI_ISL_7545676, EPI_ISL_7545677, EPI_ISL_7545678, EPI_ISL_7545679, EPI_ISL_7545680, EPI_ISL_7545681, EPI_ISL_7545682, EPI_ISL_7545683, EPI_ISL_7545684, EPI_ISL_7545685, EPI_ISL_7545686, EPI_ISL_7545687, EPI_ISL_7545688, EPI_ISL_7545689, EPI_ISL_7545690, EPI_ISL_7545691, EPI_ISL_7545692, EPI_ISL_7545693, EPI_ISL_7545694, EPI_ISL_7545695, EPI_ISL_7545696, EPI_ISL_7545698, EPI_ISL_7545699, EPI_ISL_7545700, EPI_ISL_7545701, EPI_ISL_7545702, EPI_ISL_7545703, EPI_ISL_7545704, EPI_ISL_7545705, EPI_ISL_7545706, EPI_ISL_7545707, EPI_ISL_7545708, EPI_ISL_7545709, EPI_ISL_7545710, EPI_ISL_7545711, EPI_ISL_7545712, EPI_ISL_7545713, EPI_ISL_7545714, EPI_ISL_7545715, EPI_ISL_7545716, EPI_ISL_7545717, EPI_ISL_7545718, EPI_ISL_7545719, EPI_ISL_7545720, EPI_ISL_7545721, EPI_ISL_7545722, EPI_ISL_7545723, EPI_ISL_7545724, EPI_ISL_7545725, EPI_ISL_7545726, EPI_ISL_7545727, EPI_ISL_7545728, EPI_ISL_7545729, EPI_ISL_7545730, EPI_ISL_7545731, EPI_ISL_7545732, EPI_ISL_7545733, EPI_ISL_7545734, EPI_ISL_7545735, EPI_ISL_7545736, EPI_ISL_7545737, EPI_ISL_7545738, EPI_ISL_7545739, EPI_ISL_7545742, EPI_ISL_7545743, EPI_ISL_7545744, EPI_ISL_7545745, EPI_ISL_7545746, EPI_ISL_7545747, EPI_ISL_7545748, EPI_ISL_7545749, EPI_ISL_7545750, EPI_ISL_7545751, EPI_ISL_7545752, EPI_ISL_7545753, EPI_ISL_7545754, EPI_ISL_7545755, EPI_ISL_7545756, EPI_ISL_7545757, EPI_ISL_7545758, EPI_ISL_7545759, EPI_ISL_7545760, EPI_ISL_7545761, EPI_ISL_7545762, EPI_ISL_7545764, EPI_ISL_7545765, EPI_ISL_7545766, EPI_ISL_7545767, EPI_ISL_7545768, EPI_ISL_7545769, EPI_ISL_7545770, EPI_ISL_7545771, EPI_ISL_7545772, EPI_ISL_7545773, EPI_ISL_7545774, EPI_ISL_7545775, EPI_ISL_7545776, EPI_ISL_7545777, EPI_ISL_7545778, EPI_ISL_7545779, EPI_ISL_7545780, EPI_ISL_7545781, EPI_ISL_7545782, EPI_ISL_7545783, EPI_ISL_7545784, EPI_ISL_7545785, EPI_ISL_7545787, EPI_ISL_7545788, EPI_ISL_7545789, EPI_ISL_7545790, EPI_ISL_7545791, EPI_ISL_7545792, EPI_ISL_7545793, EPI_ISL_7545794, EPI_ISL_7747496, EPI_ISL_7747497, EPI_ISL_7747498, EPI_ISL_7747499, EPI_ISL_7747500, EPI_ISL_7747501, EPI_ISL_7747502, EPI_ISL_7747535, EPI_ISL_7747536, EPI_ISL_7747537, EPI_ISL_7747538, EPI_ISL_7747539, EPI_ISL_7747540, EPI_ISL_7747541, EPI_ISL_7747542, EPI_ISL_7747543, EPI_ISL_7747544, EPI_ISL_7747545, EPI_ISL_7747546, EPI_ISL_7747547, EPI_ISL_7747548, EPI_ISL_7747549, EPI_ISL_7747550, EPI_ISL_7747551, EPI_ISL_7747552, EPI_ISL_7747553, EPI_ISL_7747554, EPI_ISL_7747555, EPI_ISL_7747556, EPI_ISL_7747557, EPI_ISL_7747558, EPI_ISL_7747559, EPI_ISL_7747560, EPI_ISL_7747561, EPI_ISL_7747562, EPI_ISL_7747563, EPI_ISL_7747564, EPI_ISL_7747565, EPI_ISL_7747566, EPI_ISL_7747567, EPI_ISL_7747568, EPI_ISL_7747569, EPI_ISL_7747570, EPI_ISL_7747571, EPI_ISL_7747572, EPI_ISL_7747573, EPI_ISL_7747574, EPI_ISL_7747575, EPI_ISL_7747576, EPI_ISL_7747577, EPI_ISL_7747578, EPI_ISL_7747579, EPI_ISL_7747580, EPI_ISL_7747581, EPI_ISL_7747582, EPI_ISL_7747583, EPI_ISL_7747584, EPI_ISL_7747585, EPI_ISL_7747586, EPI_ISL_7747587, EPI_ISL_7747588, EPI_ISL_7747589, EPI_ISL_7747590, EPI_ISL_7747591, EPI_ISL_7747592, EPI_ISL_7747593, EPI_ISL_7747594, EPI_ISL_7747595, EPI_ISL_7747596, EPI_ISL_7747597, EPI_ISL_7747598, EPI_ISL_7747599, EPI_ISL_7747600, EPI_ISL_7747601, EPI_ISL_7747602, EPI_ISL_7747603, EPI_ISL_7747604, EPI_ISL_7747605, EPI_ISL_7747606, EPI_ISL_7747607, EPI_ISL_7747608			
see above	National Health Laboratory Service, Kwazulu-Natal, South Africa	CERI, Centre for Epidemic Response and Innvoation, Stellenbosch University and KRISP, KZN Research Innovation and Sequencing Platform, UKZN.	Arisha Maharaj; Emmanuel SJ; Giandhari J; Giandhari Jennifer; Moir M; Naidoo Y; Naidoo Yeshnee; Nokukhanya Mdlalose; Pillay S; Pillay Sureshnee; Ramphal U; Ramphal Y; San JE; San James; Tegally H; Tegally Houriiyah; Tshiabuila D; Tshiabuila Derek; Upasana Ramphal; Wilkinson E; Wilkinson Eduan; Yajna Ramphal; de Oliveira T; de Oliveira Tulio; van Wyk S
EPI_ISL_4572309, EPI_ISL_4572310, EPI_ISL_4572311, EPI_ISL_4572312, EPI_ISL_4572313, EPI_ISL_4572314, EPI_ISL_4572315, EPI_ISL_4572316, EPI_ISL_4572317, EPI_ISL_4572318, EPI_ISL_4572326, EPI_ISL_4572332, EPI_ISL_4572354, EPI_ISL_4572362, EPI_ISL_4572377, EPI_ISL_4572380			
see above	National Health Laboratory Service, Kwazulu-Natal, South Africa	KRISP, KZN Research Innovation and Sequencing Platform	Giandhari Jennifer; Naidoo Yeshnee; Nokukhanya Mdlalose; Pillay Sureshnee; San James; Tegally Houriiyah; Tshiabuila Derek; Wilkinson Eduan; Yajna Ramphal; de Oliveira Tulio
EPI_ISL_6829556, EPI_ISL_6829557, EPI_ISL_6829558	National Health Laboratory Service, Kwazulu-Natal, South Africa	KRISP, KZN Research Innovation and Sequencing Platform	Arisha Maharaj; Giandhari J; Lessells R; Moir M; Naidoo Y; Nokukhanya M; Pillay S; Ramphal U; Ramphal Y; San JE; Tegally H; Tshiabuila D; Wilkinson E; de Oliveira T
EPI_ISL_5327838, EPI_ISL_5327839, EPI_ISL_5327840, EPI_ISL_5327841, EPI_ISL_5327842, EPI_ISL_5327843, EPI_ISL_5327844, EPI_ISL_5327845, EPI_ISL_5327846, EPI_ISL_5327847, EPI_ISL_5327848, EPI_ISL_5327849, EPI_ISL_5327850, EPI_ISL_5327851, EPI_ISL_5327852, EPI_ISL_5327853, EPI_ISL_5327854, EPI_ISL_5327855, EPI_ISL_5327856, EPI_ISL_5327857, EPI_ISL_5327858, EPI_ISL_5327859, EPI_ISL_5327860, EPI_ISL_5327861, EPI_ISL_5327862, EPI_ISL_5327863			
see above	National Health Laboratory Service, South Africa	CERI, Centre for Epidemic Response and Innvoation, Stellenbosch University and KRISP, KZN Research Innovation and Sequencing Platform, UKZN.	Emmanuel S; Giandhari J; Naidoo Yeshnee; Nokukhanya Mdlalose; Pillay S; Tegally H; Tshabuila Derek; Wilkinson E; Yajna Ramphal; de Oliveira T
EPI_ISL_1250424, EPI_ISL_1250425, EPI_ISL_1250427, EPI_ISL_1250428, EPI_ISL_1250430, EPI_ISL_1250431, EPI_ISL_1250432, EPI_ISL_1250434, EPI_ISL_1250436, EPI_ISL_1250437, EPI_ISL_1250439, EPI_ISL_1250441, EPI_ISL_1250442, EPI_ISL_1250444, EPI_ISL_1250446, EPI_ISL_1250447, EPI_ISL_1250448, EPI_ISL_1250450, EPI_ISL_1250452, EPI_ISL_1250453, EPI_ISL_1250455, EPI_ISL_1250456, EPI_ISL_1250458, EPI_ISL_1250460, EPI_ISL_1250462, EPI_ISL_1250463, EPI_ISL_1250465, EPI_ISL_1250466, EPI_ISL_1250468, EPI_ISL_1250469, EPI_ISL_1250472, EPI_ISL_1250474, EPI_ISL_1250476, EPI_ISL_1250477, EPI_ISL_1250479, EPI_ISL_1250480, EPI_ISL_1250482, EPI_ISL_1250483, EPI_ISL_1250485, EPI_ISL_1250486, EPI_ISL_1250488, EPI_ISL_1250490, EPI_ISL_1250491, EPI_ISL_1250493, EPI_ISL_1250494, EPI_ISL_1250496, EPI_ISL_1250497, EPI_ISL_1250499, EPI_ISL_1250501, EPI_ISL_1250502, EPI_ISL_1250504, EPI_ISL_1250505, EPI_ISL_1550912, EPI_ISL_1550913, EPI_ISL_1550914, EPI_ISL_1550915, EPI_ISL_1550916, EPI_ISL_1550917, EPI_ISL_1550918, EPI_ISL_1550919, EPI_ISL_1550920, EPI_ISL_1550921, EPI_ISL_1550922, EPI_ISL_1550923, EPI_ISL_1550924, EPI_ISL_1550925, EPI_ISL_1550926, EPI_ISL_1550927, EPI_ISL_1550928, EPI_ISL_1550929, EPI_ISL_1550930, EPI_ISL_1550931, EPI_ISL_1550932, EPI_ISL_1550933, EPI_ISL_1550934, EPI_ISL_1550935, EPI_ISL_1550936, EPI_ISL_1550937, EPI_ISL_1550938, EPI_ISL_1550939, EPI_ISL_1550940, EPI_ISL_1550941, EPI_ISL_1550942, EPI_ISL_1550943, EPI_ISL_1550944, EPI_ISL_1550945, EPI_ISL_1550946, EPI_ISL_1550947, EPI_ISL_1550948, EPI_ISL_1550949, EPI_ISL_1550950, EPI_ISL_1550951, EPI_ISL_1550952, EPI_ISL_1550953, EPI_ISL_1550954, EPI_ISL_1550955, EPI_ISL_1550956, EPI_ISL_1550957, EPI_ISL_1550958, EPI_ISL_1550959, EPI_ISL_1550960, EPI_ISL_1550961, EPI_ISL_1550962, EPI_ISL_1817755, EPI_ISL_1817756, EPI_ISL_1817757, EPI_ISL_1817758, EPI_ISL_1817759, EPI_ISL_1817760, EPI_ISL_1817761, EPI_ISL_1817762, EPI_ISL_1817763, EPI_ISL_1817764, EPI_ISL_1817765, EPI_ISL_1817766, EPI_ISL_1817767, EPI_ISL_1817768, EPI_ISL_1817769, EPI_ISL_1817770, EPI_ISL_1817771, EPI_ISL_1817772, EPI_ISL_1817773, EPI_ISL_1817774, EPI_ISL_1817775, EPI_ISL_1817776, EPI_ISL_1817777, EPI_ISL_1817778, EPI_ISL_1817779, EPI_ISL_1817780, EPI_ISL_1817781, EPI_ISL_1817782, EPI_ISL_1817783, EPI_ISL_1817784, EPI_ISL_1817785, EPI_ISL_1817786, EPI_ISL_1817787, EPI_ISL_1817788, EPI_ISL_1817789, EPI_ISL_1817790, EPI_ISL_1817791, EPI_ISL_1817792, EPI_ISL_1817793, EPI_ISL_1817794, EPI_ISL_1817795, EPI_ISL_1817796, EPI_ISL_1817797, EPI_ISL_1817798, EPI_ISL_1817799, EPI_ISL_1817800, EPI_ISL_1817801, EPI_ISL_1817802, EPI_ISL_1817803, EPI_ISL_1817804, EPI_ISL_1817805, EPI_ISL_1817806, EPI_ISL_1817807, EPI_ISL_1817808, EPI_ISL_1817809, EPI_ISL_1817810, EPI_ISL_1817811, EPI_ISL_1817812, EPI_ISL_1817813, EPI_ISL_1817814, EPI_ISL_1817815, EPI_ISL_1817816, EPI_ISL_1817817, EPI_ISL_1817818, EPI_ISL_1817819, EPI_ISL_1817820, EPI_ISL_1817821, EPI_ISL_1817822, EPI_ISL_1817823, EPI_ISL_1817824, EPI_ISL_1817825, EPI_ISL_1817826, EPI_ISL_1817827, EPI_ISL_1817828, EPI_ISL_1817829, EPI_ISL_1817830, EPI_ISL_1817831, EPI_ISL_1817832, EPI_ISL_1817833, EPI_ISL_1817834, EPI_ISL_1817835, EPI_ISL_1817836, EPI_ISL_1817837, EPI_ISL_1817838, EPI_ISL_1817839, EPI_ISL_1817840, EPI_ISL_1817841, EPI_ISL_1817842, EPI_ISL_1817843, EPI_ISL_1817844, EPI_ISL_1817845, EPI_ISL_1817846, EPI_ISL_1817848, EPI_ISL_1817849, EPI_ISL_1817850, EPI_ISL_1817851, EPI_ISL_1817852, EPI_ISL_1817853, EPI_ISL_1817854, EPI_ISL_1817855, EPI_ISL_1817856, EPI_ISL_1817857, EPI_ISL_1817858, EPI_ISL_1817859, EPI_ISL_1817860, EPI_ISL_1817861, EPI_ISL_1817862, EPI_ISL_1817863, EPI_ISL_2086228, EPI_ISL_2086229, EPI_ISL_2086230, EPI_ISL_2086231, EPI_ISL_2086232, EPI_ISL_2086233, EPI_ISL_2086234, EPI_ISL_2086235, EPI_ISL_2086236, EPI_ISL_2086237, EPI_ISL_2086238, EPI_ISL_2086239, EPI_ISL_2086240, EPI_ISL_2086241, EPI_ISL_2086242, EPI_ISL_2086243, EPI_ISL_2086244, EPI_ISL_2086245, EPI_ISL_2086246, EPI_ISL_2086247, EPI_ISL_2397000, EPI_ISL_2397001, EPI_ISL_2397002, EPI_ISL_2397003, EPI_ISL_2397004, EPI_ISL_2397005, EPI_ISL_2397006, EPI_ISL_2397007, EPI_ISL_2397008, EPI_ISL_2397009, EPI_ISL_2397010, EPI_ISL_2397011, EPI_ISL_2397012, EPI_ISL_2397013, EPI_ISL_2397014, EPI_ISL_2397015, EPI_ISL_2397016, EPI_ISL_2397017, EPI_ISL_2397018, EPI_ISL_2397019, EPI_ISL_2397020, EPI_ISL_2397021, EPI_ISL_2397022, EPI_ISL_2397023, EPI_ISL_2397024, EPI_ISL_2397025, EPI_ISL_2397026, EPI_ISL_2397027, EPI_ISL_2397028, EPI_ISL_2397029, EPI_ISL_2397030, EPI_ISL_2397031, EPI_ISL_2397032, EPI_ISL_2397033, EPI_ISL_2397034, EPI_ISL_2397035, EPI_ISL_2397036, EPI_ISL_2397037, EPI_ISL_2397038, EPI_ISL_2397039, EPI_ISL_2397040, EPI_ISL_2397041, EPI_ISL_2397042, EPI_ISL_2397043, EPI_ISL_2397044, EPI_ISL_2397045, EPI_ISL_2397046, EPI_ISL_2397047, EPI_ISL_2397048, EPI_ISL_2397049, EPI_ISL_2397050, EPI_ISL_2397051, EPI_ISL_2397052, EPI_ISL_2397053, EPI_ISL_2397054, EPI_ISL_2397055, EPI_ISL_2397056, EPI_ISL_2397057, EPI_ISL_2397058, EPI_ISL_2397059, EPI_ISL_2397060, EPI_ISL_2397061, EPI_ISL_2397062, EPI_ISL_2397063, EPI_ISL_2397064, EPI_ISL_2397065, EPI_ISL_2397066, EPI_ISL_2397067, EPI_ISL_2397068, EPI_ISL_2397069, EPI_ISL_2397070, EPI_ISL_2397071, EPI_ISL_2397072, EPI_ISL_2397073, EPI_ISL_2397074, EPI_ISL_2397075, EPI_ISL_2397076, EPI_ISL_2397077, EPI_ISL_2397078, EPI_ISL_2397079, EPI_ISL_2397080, EPI_ISL_2397081, EPI_ISL_2397082, EPI_ISL_2397083, EPI_ISL_2397084, EPI_ISL_2397085, EPI_ISL_2397086, EPI_ISL_2397087, EPI_ISL_2397088, EPI_ISL_2397089, EPI_ISL_2397090, EPI_ISL_2397091, EPI_ISL_2397092, EPI_ISL_2397093, EPI_ISL_2397094, EPI_ISL_2397095, EPI_ISL_2397096, EPI_ISL_2397097, EPI_ISL_2397098, EPI_ISL_2397099, EPI_ISL_2397100, EPI_ISL_2397101, EPI_ISL_2397102, EPI_ISL_2397103, EPI_ISL_2397104, EPI_ISL_2397105, EPI_ISL_2397106, EPI_ISL_2397107, EPI_ISL_2397108, EPI_ISL_2397109, EPI_ISL_2397110, EPI_ISL_2397111, EPI_ISL_2397112, EPI_ISL_2397113, EPI_ISL_2397114, EPI_ISL_2397115, EPI_ISL_2397116, EPI_ISL_2397117, EPI_ISL_2397118, EPI_ISL_2397119, EPI_ISL_2397120, EPI_ISL_2397121, EPI_ISL_2397122, EPI_ISL_2397123, EPI_ISL_2397124, EPI_ISL_2397125, EPI_ISL_2397126, EPI_ISL_2397127, EPI_ISL_2397128, EPI_ISL_2397129, EPI_ISL_2397130, EPI_ISL_2397131, EPI_ISL_2397132, EPI_ISL_2397133, EPI_ISL_2397134, EPI_ISL_2397135, EPI_ISL_2397136, EPI_ISL_2397137, EPI_ISL_2397138, EPI_ISL_2397139, EPI_ISL_2397140, EPI_ISL_2397141, EPI_ISL_2397142, EPI_ISL_2397143, EPI_ISL_2397144, EPI_ISL_2397145, EPI_ISL_2397146, EPI_ISL_2397147, EPI_ISL_2397148, EPI_ISL_2397149, EPI_ISL_2397150, EPI_ISL_2397151, EPI_ISL_2397152, EPI_ISL_2397153, EPI_ISL_2397154, EPI_ISL_2397155, EPI_ISL_2397156, EPI_ISL_2397157, EPI_ISL_2397158, EPI_ISL_2494729, EPI_ISL_2494753, EPI_ISL_2494755, EPI_ISL_2494770, EPI_ISL_2494771, EPI_ISL_2494772, EPI_ISL_2494773, EPI_ISL_2494775, EPI_ISL_2494778, EPI_ISL_2494779, EPI_ISL_2494780, EPI_ISL_2494781, EPI_ISL_2494784, EPI_ISL_2494785, EPI_ISL_2494786, EPI_ISL_2494787, EPI_ISL_2494789, EPI_ISL_2494790, EPI_ISL_2494791, EPI_ISL_2494792, EPI_ISL_2494793, EPI_ISL_2494795, EPI_ISL_2494796, EPI_ISL_2494798, EPI_ISL_2494799, EPI_ISL_2494800, EPI_ISL_2494801, EPI_ISL_2494802, EPI_ISL_2494803, EPI_ISL_2494804, EPI_ISL_2494805, EPI_ISL_2494806, EPI_ISL_2494807, EPI_ISL_2494808, EPI_ISL_2494809, EPI_ISL_2494810, EPI_ISL_2494811, EPI_ISL_2494812, EPI_ISL_2494813, EPI_ISL_2494814, EPI_ISL_2494815, EPI_ISL_2494816, EPI_ISL_2494817, EPI_ISL_2494818, EPI_ISL_2494819, EPI_ISL_2494820, EPI_ISL_2494821, EPI_ISL_2494823, EPI_ISL_2494824, EPI_ISL_2494825, EPI_ISL_2494826, EPI_ISL_2494827, EPI_ISL_2494828, EPI_ISL_2494829, EPI_ISL_2494832, EPI_ISL_2494833, EPI_ISL_2494834, EPI_ISL_2494835, EPI_ISL_2494836, EPI_ISL_2494837, EPI_ISL_2494838, EPI_ISL_2494839, EPI_ISL_2494840, EPI_ISL_2494841, EPI_ISL_2494842, EPI_ISL_2494843, EPI_ISL_2494845, EPI_ISL_2494846, EPI_ISL_2494847, EPI_ISL_2494848, EPI_ISL_2494849, EPI_ISL_2494850, EPI_ISL_2494851, EPI_ISL_2494852, EPI_ISL_2494853, EPI_ISL_2494854, EPI_ISL_2494855, EPI_ISL_2494856, EPI_ISL_2494857, EPI_ISL_2494858, EPI_ISL_2494860, EPI_ISL_2494861, EPI_ISL_2494863, EPI_ISL_2494865, EPI_ISL_2494866, EPI_ISL_2494869, EPI_ISL_2494873, EPI_ISL_2494875, EPI_ISL_2494876, EPI_ISL_2494877, EPI_ISL_2494883, EPI_ISL_2494886, EPI_ISL_2494887, EPI_ISL_2494888, EPI_ISL_2494890, EPI_ISL_2494892, EPI_ISL_2494893, EPI_ISL_2494894, EPI_ISL_2494895, EPI_ISL_2494896, EPI_ISL_2494897, EPI_ISL_2494904, EPI_ISL_2494905, EPI_ISL_2494908, EPI_ISL_2494909, EPI_ISL_2494910, EPI_ISL_2494911, EPI_ISL_2494923, EPI_ISL_2494927, EPI_ISL_2494930, EPI_ISL_2494935, EPI_ISL_2494936, EPI_ISL_2494939, EPI_ISL_2494940, EPI_ISL_2494945, EPI_ISL_2494948, EPI_ISL_2494959, EPI_ISL_2494965, EPI_ISL_2494978, EPI_ISL_2494998, EPI_ISL_2495028, EPI_ISL_2495032, EPI_ISL_2495046, EPI_ISL_2495047, EPI_ISL_2495049, EPI_ISL_2495050, EPI_ISL_2495051, EPI_ISL_2495052, EPI_ISL_2495053, EPI_ISL_3663498, EPI_ISL_3663499, EPI_ISL_3663500, EPI_ISL_3663501, EPI_ISL_3663502, EPI_ISL_3663503, EPI_ISL_3663504, EPI_ISL_3663505, EPI_ISL_3663506, EPI_ISL_3663507, EPI_ISL_3663508, EPI_ISL_3663509, EPI_ISL_3663510, EPI_ISL_3663511, EPI_ISL_3663512, EPI_ISL_3663513, EPI_ISL_3663514, EPI_ISL_3663515, EPI_ISL_3663516, EPI_ISL_3663517, EPI_ISL_3663518, EPI_ISL_3663519, EPI_ISL_3663520, EPI_ISL_3663521, EPI_ISL_3663522, EPI_ISL_3663523, EPI_ISL_3663524, EPI_ISL_3663525, EPI_ISL_3663526, EPI_ISL_3663527, EPI_ISL_3663528, EPI_ISL_3663529, EPI_ISL_3663530, EPI_ISL_3663531, EPI_ISL_3663532, EPI_ISL_3663533, EPI_ISL_3663534, EPI_ISL_3663535, EPI_ISL_3663536, EPI_ISL_3663537, EPI_ISL_3663538, EPI_ISL_3663539, EPI_ISL_3663540, EPI_ISL_3663541, EPI_ISL_3663542, EPI_ISL_3663543, EPI_ISL_3663544			
see above	National Health Laboratory Service, South Africa	KRISP, KZN Research Innovation and Sequencing Platform	Emmanuel SJ; Giandhari J; Giandhari Jennifer; Khan S; Lessells R; Maslo C; Mdlalose K; Naidoo Y; Naidoo Yeshnee; Pillay S; Pillay Sureshnee; Ramphal U; San James; Sitharam L; Tegally H; Tegally Houriiyah; Tshabuila Derek; Wilkinson E; Wilkinson Eduan; Yajna Ramphal; York D; de Oliveira T; de Oliveira Tulio
EPI_ISL_1048462, EPI_ISL_1048463, EPI_ISL_1048464, EPI_ISL_1048465, EPI_ISL_1048466, EPI_ISL_1048467, EPI_ISL_1048468, EPI_ISL_1048469, EPI_ISL_1048470, EPI_ISL_1048471, EPI_ISL_1048472, EPI_ISL_1048473, EPI_ISL_1048474, EPI_ISL_1048475, EPI_ISL_1048476, EPI_ISL_1048477, EPI_ISL_1048478, EPI_ISL_1048479, EPI_ISL_1048480, EPI_ISL_1048481, EPI_ISL_1048482, EPI_ISL_1048483,			

EPI_ISL_1048484, EPI_ISL_1048485, EPI_ISL_1048486, EPI_ISL_1048487, EPI_ISL_1048488, EPI_ISL_1048489, EPI_ISL_1048490, EPI_ISL_1048491, EPI_ISL_1048492, EPI_ISL_1048493, EPI_ISL_1048494, EPI_ISL_1048495, EPI_ISL_1048496, EPI_ISL_1048497, EPI_ISL_1048498, EPI_ISL_1048499, EPI_ISL_1048500, EPI_ISL_1048501, EPI_ISL_1048502, EPI_ISL_1048503, EPI_ISL_1048504, EPI_ISL_1048505, EPI_ISL_1048506, EPI_ISL_1048507, EPI_ISL_1048508, EPI_ISL_1048509, EPI_ISL_1048510, EPI_ISL_1048511, EPI_ISL_1048512, EPI_ISL_1048513, EPI_ISL_1048514, EPI_ISL_1048515, EPI_ISL_1048516, EPI_ISL_1048517, EPI_ISL_1048518, EPI_ISL_1048519, EPI_ISL_1048520, EPI_ISL_1048521, EPI_ISL_1048522, EPI_ISL_1048523, EPI_ISL_1048524, EPI_ISL_1048525, EPI_ISL_1048526, EPI_ISL_1048527, EPI_ISL_1048528, EPI_ISL_1048529, EPI_ISL_1048530, EPI_ISL_1048531, EPI_ISL_1048532, EPI_ISL_1048533, EPI_ISL_1048534, EPI_ISL_1048535, EPI_ISL_1048536, EPI_ISL_1048537, EPI_ISL_1048538, EPI_ISL_1048539, EPI_ISL_1048540, EPI_ISL_1048541, EPI_ISL_1048542, EPI_ISL_1048543, EPI_ISL_1048544, EPI_ISL_1048545, EPI_ISL_1048546, EPI_ISL_1048547, EPI_ISL_1048548, EPI_ISL_1048549, EPI_ISL_1048550, EPI_ISL_1048551, EPI_ISL_1048552, EPI_ISL_1048553, EPI_ISL_1048554, EPI_ISL_1048555, EPI_ISL_1048556, EPI_ISL_1048557, EPI_ISL_1048558, EPI_ISL_1048559, EPI_ISL_1048560, EPI_ISL_1048561, EPI_ISL_1048562, EPI_ISL_1048563, EPI_ISL_1048564, EPI_ISL_1048565, EPI_ISL_1048566, EPI_ISL_1048567, EPI_ISL_1048568, EPI_ISL_1371906, EPI_ISL_1371907, EPI_ISL_1371908, EPI_ISL_1371909, EPI_ISL_1371910, EPI_ISL_1371911, EPI_ISL_1371912, EPI_ISL_1371913, EPI_ISL_1371914, EPI_ISL_1371915, EPI_ISL_1371916, EPI_ISL_1371917, EPI_ISL_1371918, EPI_ISL_1371919, EPI_ISL_1371920, EPI_ISL_1371921, EPI_ISL_1371922, EPI_ISL_1371923, EPI_ISL_1371924, EPI_ISL_1371925, EPI_ISL_1371926, EPI_ISL_1371927, EPI_ISL_1371928, EPI_ISL_1371929, EPI_ISL_1371930, EPI_ISL_1371931, EPI_ISL_1371932, EPI_ISL_1371933, EPI_ISL_1371934, EPI_ISL_1371935, EPI_ISL_1371936, EPI_ISL_1371937, EPI_ISL_1371938, EPI_ISL_1371939, EPI_ISL_1371940, EPI_ISL_1371941, EPI_ISL_1371942, EPI_ISL_1371943, EPI_ISL_1371944, EPI_ISL_1371945, EPI_ISL_1371946, EPI_ISL_1371947, EPI_ISL_1371948, EPI_ISL_1371949, EPI_ISL_1371950, EPI_ISL_1371951, EPI_ISL_1371952, EPI_ISL_1371953, EPI_ISL_1371954, EPI_ISL_1371955, EPI_ISL_1371956, EPI_ISL_1371957, EPI_ISL_1371958, EPI_ISL_1371959, EPI_ISL_1371960, EPI_ISL_1371961, EPI_ISL_1371962, EPI_ISL_1371963, EPI_ISL_1371964, EPI_ISL_1371965, EPI_ISL_1371966, EPI_ISL_1371967, EPI_ISL_1371968, EPI_ISL_1371969, EPI_ISL_1371970, EPI_ISL_1371971, EPI_ISL_1371972, EPI_ISL_1371973, EPI_ISL_1371974, EPI_ISL_1371975, EPI_ISL_1371976, EPI_ISL_1371977, EPI_ISL_1371978, EPI_ISL_1371979, EPI_ISL_1371980, EPI_ISL_1371981, EPI_ISL_1371982, EPI_ISL_1371983, EPI_ISL_1371984, EPI_ISL_1371985, EPI_ISL_1371986, EPI_ISL_1371987, EPI_ISL_1371988, EPI_ISL_1371989, EPI_ISL_1371990, EPI_ISL_1371991, EPI_ISL_1371992, EPI_ISL_1371993, EPI_ISL_1371994, EPI_ISL_1371995, EPI_ISL_1371996, EPI_ISL_1371997, EPI_ISL_1371998, EPI_ISL_1371999, EPI_ISL_1372000, EPI_ISL_1372001, EPI_ISL_1372002, EPI_ISL_1372003, EPI_ISL_1372004, EPI_ISL_1372005, EPI_ISL_1372006, EPI_ISL_2617186, EPI_ISL_2617187, EPI_ISL_2617188, EPI_ISL_2617189, EPI_ISL_2617190, EPI_ISL_2617191, EPI_ISL_2617192, EPI_ISL_2617193, EPI_ISL_2617194, EPI_ISL_2617195, EPI_ISL_2617196, EPI_ISL_2617197, EPI_ISL_2617198, EPI_ISL_2617199, EPI_ISL_2617200, EPI_ISL_2617201, EPI_ISL_2617202, EPI_ISL_2617203, EPI_ISL_2617204, EPI_ISL_2617205, EPI_ISL_2617206, EPI_ISL_2617207, EPI_ISL_2617208, EPI_ISL_2617209, EPI_ISL_2617210, EPI_ISL_2617211, EPI_ISL_2617212, EPI_ISL_2617213, EPI_ISL_2617214, EPI_ISL_2617215, EPI_ISL_2617216, EPI_ISL_2688531, EPI_ISL_2688532, EPI_ISL_2688533, EPI_ISL_2688534, EPI_ISL_2688535, EPI_ISL_2688536, EPI_ISL_2688537, EPI_ISL_2688538, EPI_ISL_2688539, EPI_ISL_2688540, EPI_ISL_2688541, EPI_ISL_2688542, EPI_ISL_2688543, EPI_ISL_2688544, EPI_ISL_2688545, EPI_ISL_2688546, EPI_ISL_2688549, EPI_ISL_2688550, EPI_ISL_2688551, EPI_ISL_2688552, EPI_ISL_2688553, EPI_ISL_2688554, EPI_ISL_2688555, EPI_ISL_2688556, EPI_ISL_2688557, EPI_ISL_2688558, EPI_ISL_2688559, EPI_ISL_2688560, EPI_ISL_2688561, EPI_ISL_2688562, EPI_ISL_2688563, EPI_ISL_2688564, EPI_ISL_2688565, EPI_ISL_2688566, EPI_ISL_2688567, EPI_ISL_2688568, EPI_ISL_2688569, EPI_ISL_2688570, EPI_ISL_2688571, EPI_ISL_2688572, EPI_ISL_2688573, EPI_ISL_2688574, EPI_ISL_2688575, EPI_ISL_2899687, EPI_ISL_2899688, EPI_ISL_2899689, EPI_ISL_2899690, EPI_ISL_2899691, EPI_ISL_2899692, EPI_ISL_2899693, EPI_ISL_2899694, EPI_ISL_2899695, EPI_ISL_2899696, EPI_ISL_2899697, EPI_ISL_2899698, EPI_ISL_2899699, EPI_ISL_2899700, EPI_ISL_2899701, EPI_ISL_2899702, EPI_ISL_2899703, EPI_ISL_2899704, EPI_ISL_2899705, EPI_ISL_2899706, EPI_ISL_2899707, EPI_ISL_2899708, EPI_ISL_2899709, EPI_ISL_2899710, EPI_ISL_2899711, EPI_ISL_2899712, EPI_ISL_2899713, EPI_ISL_2899714, EPI_ISL_2899715, EPI_ISL_2899716, EPI_ISL_2899717, EPI_ISL_2899718, EPI_ISL_2899719, EPI_ISL_2899720, EPI_ISL_2899721, EPI_ISL_2899722, EPI_ISL_2899723, EPI_ISL_2899724, EPI_ISL_2899725, EPI_ISL_2899726, EPI_ISL_2899727, EPI_ISL_2899728, EPI_ISL_2899729, EPI_ISL_2899730, EPI_ISL_2899731, EPI_ISL_2899732, EPI_ISL_2899733, EPI_ISL_2899734, EPI_ISL_2899735, EPI_ISL_2899736, EPI_ISL_2899737, EPI_ISL_2899738, EPI_ISL_2899739, EPI_ISL_2899740, EPI_ISL_3132574, EPI_ISL_3132575, EPI_ISL_3132576, EPI_ISL_3132577, EPI_ISL_3132578, EPI_ISL_3132579, EPI_ISL_3132580, EPI_ISL_3132581, EPI_ISL_3132582, EPI_ISL_3132583, EPI_ISL_3132584, EPI_ISL_3132585, EPI_ISL_3132586, EPI_ISL_3132587, EPI_ISL_3132588, EPI_ISL_3132589, EPI_ISL_3132590, EPI_ISL_3132591, EPI_ISL_3132592, EPI_ISL_3132593, EPI_ISL_3132594, EPI_ISL_3132595, EPI_ISL_3132596, EPI_ISL_3132597, EPI_ISL_3132598, EPI_ISL_3132599, EPI_ISL_3132600, EPI_ISL_3132601, EPI_ISL_3132602, EPI_ISL_3132603, EPI_ISL_3132604, EPI_ISL_3132605, EPI_ISL_3132606, EPI_ISL_3132607, EPI_ISL_3132608, EPI_ISL_3132609, EPI_ISL_3132610, EPI_ISL_3132611, EPI_ISL_3132612, EPI_ISL_3132613, EPI_ISL_3132614, EPI_ISL_3132615, EPI_ISL_3132616, EPI_ISL_3132617, EPI_ISL_3132618, EPI_ISL_3132619, EPI_ISL_3132620, EPI_ISL_3132621, EPI_ISL_3132622, EPI_ISL_3730295, EPI_ISL_3730296, EPI_ISL_3730298, EPI_ISL_3730300, EPI_ISL_3730301, EPI_ISL_3730302, EPI_ISL_3730303, EPI_ISL_3730304, EPI_ISL_3730305, EPI_ISL_3730306, EPI_ISL_3730307, EPI_ISL_3730308, EPI_ISL_3730309, EPI_ISL_3730310, EPI_ISL_3730311, EPI_ISL_3730312, EPI_ISL_3730313, EPI_ISL_3730315, EPI_ISL_3730316, EPI_ISL_3730318, EPI_ISL_3730320, EPI_ISL_3730322, EPI_ISL_3730323, EPI_ISL_3730324, EPI_ISL_3730325, EPI_ISL_3730326, EPI_ISL_3730327, EPI_ISL_3730328, EPI_ISL_3730329, EPI_ISL_3730331, EPI_ISL_3730333, EPI_ISL_3730334, EPI_ISL_3730336, EPI_ISL_3730337, EPI_ISL_3730339, EPI_ISL_3730340, EPI_ISL_3730341, EPI_ISL_3730342, EPI_ISL_3730343, EPI_ISL_3730347, EPI_ISL_3730348, EPI_ISL_3730349, EPI_ISL_3730350, EPI_ISL_3730351, EPI_ISL_3730352, EPI_ISL_3730354, EPI_ISL_3730356, EPI_ISL_3730357, EPI_ISL_3730358, EPI_ISL_3730361, EPI_ISL_3730362, EPI_ISL_3730363, EPI_ISL_3730364, EPI_ISL_3730365, EPI_ISL_3730367, EPI_ISL_3730368, EPI_ISL_3730370, EPI_ISL_3730371, EPI_ISL_3799011, EPI_ISL_3799012, EPI_ISL_3799015, EPI_ISL_3799020, EPI_ISL_3799021, EPI_ISL_3799023, EPI_ISL_3799024, EPI_ISL_3799025, EPI_ISL_3799026, EPI_ISL_3799029, EPI_ISL_3799030, EPI_ISL_3799031, EPI_ISL_3799032, EPI_ISL_3799033, EPI_ISL_3799034, EPI_ISL_3799037, EPI_ISL_3799038, EPI_ISL_3799039, EPI_ISL_3799041, EPI_ISL_3799042, EPI_ISL_3799043, EPI_ISL_3799044, EPI_ISL_3799045, EPI_ISL_3799046, EPI_ISL_3799047, EPI_ISL_3799048, EPI_ISL_3799049, EPI_ISL_3799050, EPI_ISL_3799051, EPI_ISL_3799052, EPI_ISL_3799053, EPI_ISL_3799054, EPI_ISL_3799055, EPI_ISL_3799057, EPI_ISL_3799058, EPI_ISL_3799059, EPI_ISL_3799060, EPI_ISL_3799061, EPI_ISL_3799065, EPI_ISL_3799067, EPI_ISL_3799068, EPI_ISL_3799070, EPI_ISL_3799073, EPI_ISL_3799075, EPI_ISL_3799077, EPI_ISL_3799079, EPI_ISL_3799081, EPI_ISL_3799087, EPI_ISL_3799091, EPI_ISL_3799093, EPI_ISL_3799097, EPI_ISL_3799099, EPI_ISL_3799101, EPI_ISL_3799102, EPI_ISL_3799106, EPI_ISL_3799108, EPI_ISL_3799111, EPI_ISL_3799112, EPI_ISL_3799114, EPI_ISL_3799118, EPI_ISL_3799120, EPI_ISL_3799126, EPI_ISL_3799130, EPI_ISL_3799132, EPI_ISL_3799133, EPI_ISL_3799135, EPI_ISL_3799141, EPI_ISL_3799147, EPI_ISL_3799149, EPI_ISL_3799150, EPI_ISL_3799152, EPI_ISL_3799154, EPI_ISL_3799157, EPI_ISL_3799163, EPI_ISL_3799164, EPI_ISL_3799167, EPI_ISL_3799172, EPI_ISL_3799173, EPI_ISL_3799174, EPI_ISL_3799175, EPI_ISL_3799176, EPI_ISL_3799177, EPI_ISL_3799178, EPI_ISL_3799179, EPI_ISL_3799180, EPI_ISL_3799181, EPI_ISL_3799182, EPI_ISL_3799183, EPI_ISL_3799184, EPI_ISL_3799185, EPI_ISL_3939037, EPI_ISL_3939038, EPI_ISL_3939039, EPI_ISL_3939040, EPI_ISL_3939041, EPI_ISL_3939042, EPI_ISL_3939043, EPI_ISL_3939044, EPI_ISL_3939045, EPI_ISL_3939046, EPI_ISL_3939047, EPI_ISL_3939048, EPI_ISL_3939049, EPI_ISL_3939050, EPI_ISL_3939051, EPI_ISL_3939052, EPI_ISL_3939053, EPI_ISL_3939054, EPI_ISL_3939055, EPI_ISL_3939056, EPI_ISL_3939057, EPI_ISL_3939058, EPI_ISL_3939059, EPI_ISL_3939060, EPI_ISL_3939061, EPI_ISL_3939062, EPI_ISL_3939063, EPI_ISL_3939065, EPI_ISL_3939066, EPI_ISL_3939067, EPI_ISL_3939069, EPI_ISL_3939070, EPI_ISL_3939071, EPI_ISL_3939072, EPI_ISL_3939073, EPI_ISL_3939074, EPI_ISL_3939075, EPI_ISL_3939076, EPI_ISL_3939077, EPI_ISL_3939078, EPI_ISL_3939079, EPI_ISL_3939080, EPI_ISL_3939081, EPI_ISL_3939082, EPI_ISL_3939083, EPI_ISL_3939084, EPI_ISL_3939085, EPI_ISL_3939086, EPI_ISL_3939087, EPI_ISL_3939089, EPI_ISL_3939090, EPI_ISL_3939091, EPI_ISL_3939092, EPI_ISL_3939093, EPI_ISL_3939094, EPI_ISL_3939095, EPI_ISL_3939096, EPI_ISL_3939097, EPI_ISL_3939098, EPI_ISL_3939099, EPI_ISL_3939100, EPI_ISL_3939101, EPI_ISL_4121561, EPI_ISL_4121575, EPI_ISL_4121585, EPI_ISL_4121588, EPI_ISL_4121591, EPI_ISL_4121595, EPI_ISL_4121598, EPI_ISL_4121601, EPI_ISL_4121603, EPI_ISL_4121615, EPI_ISL_4121624, EPI_ISL_4121628, EPI_ISL_4121631, EPI_ISL_4121646, EPI_ISL_4121649, EPI_ISL_4121653, EPI_ISL_4121690, EPI_ISL_4121694, EPI_ISL_4121697, EPI_ISL_4121700, EPI_ISL_4121704, EPI_ISL_4121707, EPI_ISL_4121716, EPI_ISL_4121719, EPI_ISL_4121722, EPI_ISL_4121725, EPI_ISL_4121737, EPI_ISL_4121742, EPI_ISL_4121746, EPI_ISL_4121749, EPI_ISL_4121758, EPI_ISL_4121761, EPI_ISL_4121767, EPI_ISL_4121773, EPI_ISL_4121776, EPI_ISL_4121782, EPI_ISL_4121789, EPI_ISL_4121793, EPI_ISL_4336894, EPI_ISL_4336897, EPI_ISL_4336900, EPI_ISL_4336902, EPI_ISL_4336905, EPI_ISL_4336908, EPI_ISL_4336910, EPI_ISL_4336913, EPI_ISL_4336915, EPI_ISL_4336918, EPI_ISL_4336921, EPI_ISL_4336923, EPI_ISL_4336926, EPI_ISL_4336929, EPI_ISL_4336931, EPI_ISL_4336935, EPI_ISL_4336938, EPI_ISL_4336941, EPI_ISL_4336943, EPI_ISL_4336945, EPI_ISL_4336948, EPI_ISL_4336951, EPI_ISL_4336954, EPI_ISL_4336957, EPI_ISL_4336959			
see above	National Health Laboratory Service, South Africa	KRISP, KZn Research Innovation and Sequencing Platform	Emmanuel S; Emmanuel SJ; Giandhari J; Giandhari Jennifer; Khan S; Lessells R; Maslo C; Mdlalose K; Naidoo Yeshnee; Pillay S; Pillay Sureshnee; San James; Sitharam L; Tegally H; Tegally Houriiyah; Tshabuila Derek; Tshiabuila Derek; Wilkinson E; Wilkinson Eduan; Yajna Ramphal; York D; de Oliveira T; de Oliveira Tulio
EPI_ISL_4891491, EPI_ISL_4891508, EPI_ISL_4891550, EPI_ISL_4891582, EPI_ISL_4891589, EPI_ISL_4891599, EPI_ISL_4891608, EPI_ISL_4891609, EPI_ISL_4891628, EPI_ISL_4891673, EPI_ISL_4891697, EPI_ISL_4891706, EPI_ISL_4891860, EPI_ISL_4891870, EPI_ISL_4891871, EPI_ISL_4891872, EPI_ISL_4891873, EPI_ISL_4891874, EPI_ISL_4891876, EPI_ISL_4891877, EPI_ISL_4891878, EPI_ISL_4891888, EPI_ISL_4891889, EPI_ISL_7456449			
see above	National Institute For Communicable Diseases Of The National Health Laboratory Service	National Institute for Communicable Diseases of the National Health Laboratory Service	Amoako DG; Bhiman JN; Everatt J; Ismail A; Mahlangu B; Mnguni A; Mohale T; Ntuli N; Scheepers C; Wolter N
EPI_ISL_2433846, EPI_ISL_2447877, EPI_ISL_2447879, EPI_ISL_2447891, EPI_ISL_2447892, EPI_ISL_2447895, EPI_ISL_3451535, EPI_ISL_3451536, EPI_ISL_3451537, EPI_ISL_3451538, EPI_ISL_3451539, EPI_ISL_3451540, EPI_ISL_3451541, EPI_ISL_3451542, EPI_ISL_3451543, EPI_ISL_3451544, EPI_ISL_3451545, EPI_ISL_3451546, EPI_ISL_3451547, EPI_ISL_3451548, EPI_ISL_3451549, EPI_ISL_3451550, EPI_ISL_3451551, EPI_ISL_3451552, EPI_ISL_3451553, EPI_ISL_3451554, EPI_ISL_3451555, EPI_ISL_3451556, EPI_ISL_3451557, EPI_ISL_3451558, EPI_ISL_3451559, EPI_ISL_3451560, EPI_ISL_3451561, EPI_ISL_3451562, EPI_ISL_3451563, EPI_ISL_3451564, EPI_ISL_3451565, EPI_ISL_3451566, EPI_ISL_3451595, EPI_ISL_3451596, EPI_ISL_3451597, EPI_ISL_3451598, EPI_ISL_3451599, EPI_ISL_3451600, EPI_ISL_3451601, EPI_ISL_3451602, EPI_ISL_3451603, EPI_ISL_3451604, EPI_ISL_3451605, EPI_ISL_3451606, EPI_ISL_3451607, EPI_ISL_3451608, EPI_ISL_3451609, EPI_ISL_3451610, EPI_ISL_3451611, EPI_ISL_3451612, EPI_ISL_3451613, EPI_ISL_3451614, EPI_ISL_3451615, EPI_ISL_3451616, EPI_ISL_3643813, EPI_ISL_3643828, EPI_ISL_3643839, EPI_ISL_3643858, EPI_ISL_3643874, EPI_ISL_3643908, EPI_ISL_3643920, EPI_ISL_3644042, EPI_ISL_3644043, EPI_ISL_3644045, EPI_ISL_3644047, EPI_ISL_3644048, EPI_ISL_3717893, EPI_ISL_3717894, EPI_ISL_3717897, EPI_ISL_3717902, EPI_ISL_3717904, EPI_ISL_3717905, EPI_ISL_3717926, EPI_ISL_3717937, EPI_ISL_3717939, EPI_ISL_3717944, EPI_ISL_3717945, EPI_ISL_3717949, EPI_ISL_3717959, EPI_ISL_3717960, EPI_ISL_3717962, EPI_ISL_3717970, EPI_ISL_3717992, EPI_ISL_3717996, EPI_ISL_3718049, EPI_ISL_3718051, EPI_ISL_3718052, EPI_ISL_3718053, EPI_ISL_3718054, EPI_ISL_3718055, EPI_ISL_3718056, EPI_ISL_3718057, EPI_ISL_3718058, EPI_ISL_3718059, EPI_ISL_3718060, EPI_ISL_3718061, EPI_ISL_3718062, EPI_ISL_4029931, EPI_ISL_4029936, EPI_ISL_4029953, EPI_ISL_4029957, EPI_ISL_4029976, EPI_ISL_4029978, EPI_ISL_4030018, EPI_ISL_4030079, EPI_ISL_4030080, EPI_ISL_4030081, EPI_ISL_4030082, EPI_ISL_4030083, EPI_ISL_4030106, EPI_ISL_4030107, EPI_ISL_4030108, EPI_ISL_4030109, EPI_ISL_4030110, EPI_ISL_4030117, EPI_ISL_4253673, EPI_ISL_4253674, EPI_ISL_4253675, EPI_ISL_4253676, EPI_ISL_4253677, EPI_ISL_4253678, EPI_ISL_4253679, EPI_ISL_4253680, EPI_ISL_4253681, EPI_ISL_4253682, EPI_ISL_4253683, EPI_ISL_4253684, EPI_ISL_4253685, EPI_ISL_4253686, EPI_ISL_4253687, EPI_ISL_4253688, EPI_ISL_4253689, EPI_ISL_4651523, EPI_ISL_4651531, EPI_ISL_4651532, EPI_ISL_4651540, EPI_ISL_4651548, EPI_ISL_4651554, EPI_ISL_4651559, EPI_ISL_4651565, EPI_ISL_4651574, EPI_ISL_4651575, EPI_ISL_4651580, EPI_ISL_4651581, EPI_ISL_4651588, EPI_ISL_4651595, EPI_ISL_4651596, EPI_ISL_4651601, EPI_ISL_4651602, EPI_ISL_4651610, EPI_ISL_4651616, EPI_ISL_6204412, EPI_ISL_6886554, EPI_ISL_8002897, EPI_ISL_8002898, EPI_ISL_8051878			
see above	National Institute for Communicable Diseases of the National Health Laboratory Service	National Institute for Communicable Diseases of the National Health Laboratory Service	Amoako DG; Bhiman JN; Everatt J; Ismail A; Kekana D; Mahlangu B; Mnguni A; Mohale T; Ntuli N; Scheepers C; Wolter N
EPI_ISL_1239851, EPI_ISL_1239853, EPI_ISL_1239854, EPI_ISL_1239856, EPI_ISL_1239857, EPI_ISL_1239858, EPI_ISL_1239859, EPI_ISL_1239860, EPI_ISL_1239862, EPI_ISL_1239863, EPI_ISL_1239865, EPI_ISL_1239868, EPI_ISL_1239869, EPI_ISL_1239871, EPI_ISL_1239872, EPI_ISL_1239873, EPI_ISL_1239877, EPI_ISL_1239878, EPI_ISL_1239880, EPI_ISL_1239881			
see above	National Institute for Communicable Diseases,National Health Laboratory Services, Gauteng, South Africa	National Institute for Communicable Diseases of the National Health Laboratory Service	Amoako DG; Bhiman JN; Ismail A; Mahlangu B; Mohale T; Ntuli N; Scheepers C
EPI_ISL_2955500, EPI_ISL_3118697	Netcare	KRISP, KZn Research Innovation and Sequencing Platform	Brown MJ; Emmanuel SJ; Giandhari J; Lessells R; Naidoo Y; Omar S; Pillay S; Ramphal U; Tegally H; Wadula J; Wilkinson E; Yajna R; de Oliveira T
EPI_ISL_2770489	Netcare Kingsway	KRISP, KZN Research Innovation and Sequencing Platform	Giandhari Jennifer; Naidoo Yeshnee; Pillay Sureshnee; San James; Tegally Houriiyah; Tshabuila Derek; Wilkinson Eduan; Yajna Ramphal; de Oliveira Tulio
EPI_ISL_2693062	Netcare Kingsway Hospital	KRISP, KZN Research Innovation and Sequencing Platform	Giandhari Jennifer; Naidoo Yeshnee; Pillay Sureshnee; San James; Tegally Houriiyah; Tshabuila Derek; Wilkinson Eduan; Yajna Ramphal; de Oliveira Tulio
EPI_ISL_2841651, EPI_ISL_3132628	Netcare Kingsway Hospital	KRISP, KZn Research Innovation and Sequencing Platform	Giandhari Jennifer; Naidoo Yeshnee; Pillay Sureshnee; San James; Tegally Houriiyah; Tshabuila Derek; Wilkinson Eduan; Yajna Ramphal; de Oliveira Tulio
EPI_ISL_3132629	Netcare Margate	KRISP, KZn Research Innovation and Sequencing Platform	Giandhari Jennifer; Naidoo Yeshnee; Pillay Sureshnee; San James; Tegally Houriiyah; Tshabuila Derek; Wilkinson Eduan; Yajna Ramphal; de Oliveira Tulio
EPI_ISL_3132630	Netcare Parklands Hospital	KRISP, KZn Research Innovation and Sequencing Platform	Giandhari Jennifer; Naidoo Yeshnee; Pillay Sureshnee; San James; Tegally Houriiyah; Tshabuila Derek; Wilkinson Eduan; Yajna Ramphal; de Oliveira Tulio
EPI_ISL_3132632, EPI_ISL_3132633, EPI_ISL_3132634	Netcare St Augustines	KRISP, KZn Research Innovation and Sequencing Platform	Giandhari Jennifer; Naidoo Yeshnee; Pillay Sureshnee; San James; Tegally Houriiyah; Tshabuila Derek; Wilkinson Eduan; Yajna Ramphal; de Oliveira Tulio
EPI_ISL_2494975, EPI_ISL_2501091, EPI_ISL_2501092, EPI_ISL_2501093, EPI_ISL_2501094, EPI_ISL_2501095	Netcare St Augustines Hospital	KRISP, KZN Research Innovation and Sequencing Platform	Emmanuel SJ; Giandhari J; Khan S; Lessells R; Mdlalose K; Naidoo Y; Pillay S; Ramphal U; Sisonke Team; Tegally H; Wilkinson E; York D; de Oliveira T
EPI_ISL_2841650	Netcare The Bay Hospital	KRISP, KZn Research Innovation and Sequencing Platform	Giandhari Jennifer; Naidoo Yeshnee; Pillay Sureshnee; San James; Tegally Houriiyah; Tshabuila Derek; Wilkinson Eduan; Yajna Ramphal; de Oliveira Tulio
EPI_ISL_3132631	Netcare Umhlanga - Chronic patient	KRISP, KZn Research Innovation and Sequencing Platform	Giandhari Jennifer; Naidoo Yeshnee; Pillay Sureshnee; San James; Tegally Houriiyah; Tshabuila Derek; Wilkinson Eduan; Yajna Ramphal; de Oliveira Tulio
EPI_ISL_2727404, EPI_ISL_3730372	Netcare Umhlanga Hospital	KRISP, KZn Research Innovation and Sequencing Platform	Giandhari Jennifer; Naidoo Yeshnee; Pillay Sureshnee; San James; Tegally Houriiyah; Tshabuila Derek; Tshiabuila Derek; Wilkinson Eduan; Yajna Ramphal; de Oliveira Tulio
EPI_ISL_3838638	Private/Pathcare	National Institute for Communicable Diseases of the National Health Laboratory Service	Amoako DG; Bhiman JN; Everatt J; Ismail A; Mahlangu B; Mnguni A; Mohale T; Ntuli N; Scheepers C; Sisonke Team
EPI_ISL_7605682	ROB FERREIRA LABORATORY	National Institute for Communicable Diseases of the National Health Laboratory Service	Amoako DG; Bhiman JN; Everatt J; Ismail A; Mahlangu B; Mnguni A; Mohale T; Ntuli N; Scheepers C; Wolter N
EPI_ISL_2086220, EPI_ISL_2086221, EPI_ISL_2086222, EPI_ISL_2086223, EPI_ISL_2086224, EPI_ISL_2086225, EPI_ISL_2086226, EPI_ISL_2086227			
see above	ShipMed Inc	KRISP, KZN Research Innovation and Sequencing Platform	Emmanuel SJ; Giandhari J; Lessells R; Naidoo Y; Pillay S; Ramphal U; Tegally H; Wilkinson E; de Oliveira T
EPI_ISL_2271962	Tshepong Hospital	National Institute for Communicable Diseases of the National Health Laboratory Service	Amoako DG; Bhiman JN; Ismail A; Mahlangu B; Mohale T; Ntuli N; Scheepers C
EPI_ISL_1324122, EPI_ISL_1324128, EPI_ISL_1324129	UW Virology Lab	UW Virology Lab	Alexander Greninger; Hong Xie; Keith R Jerome; Lasata Shrestha; Margaret Mills; Meei-Li Huang; Michelle Lin; Noah Baker; Pavitra Roychoudhury; Saraswathi Sathees; Sean Ellis; Shah Mohamed Bakhash
EPI_ISL_2494712, EPI_ISL_2494713, EPI_ISL_2494741, EPI_ISL_2494744, EPI_ISL_2494752, EPI_ISL_2494759, EPI_ISL_2494764, EPI_ISL_2494769, EPI_ISL_2494831, EPI_ISL_2494864, EPI_ISL_2494901, EPI_ISL_2494903, EPI_ISL_2494913, EPI_ISL_2494916, EPI_ISL_2494917, EPI_ISL_2494918, EPI_ISL_2494920, EPI_ISL_2494921, EPI_ISL_2494922, EPI_ISL_2494925, EPI_ISL_2494929, EPI_ISL_2494932, EPI_ISL_2494934, EPI_ISL_2494938, EPI_ISL_2494950, EPI_ISL_2494953, EPI_ISL_2494956, EPI_ISL_2494957, EPI_ISL_2494958, EPI_ISL_2494963, EPI_ISL_2494964, EPI_ISL_2494969, EPI_ISL_2494971, EPI_ISL_2494977, EPI_ISL_2494979, EPI_ISL_2494982, EPI_ISL_2494985, EPI_ISL_2494989, EPI_ISL_2494993, EPI_ISL_2495003, EPI_ISL_2495054			
see above	Vaccines and Infectious Diseases Analytics Research Unit (VIDA)	KRISP, KZN Research Innovation and Sequencing Platform	Baillie Vicky; Giandhari Jennifer; Madhi Shabir; Naidoo Yeshnee; Pillay Sureshnee; San James; Tegally Houriiyah; Wilkinson Eduan; de Oliveira Tulio; du Plessis Jeanine
EPI_ISL_2360343, EPI_ISL_2360344, EPI_ISL_2360345, EPI_ISL_2360346, EPI_ISL_2360347, EPI_ISL_2360349, EPI_ISL_2360351, EPI_ISL_2360352, EPI_ISL_2360354, EPI_ISL_2360356, EPI_ISL_2360357, EPI_ISL_2360358, EPI_ISL_2360359, EPI_ISL_2360361, EPI_ISL_2360364, EPI_ISL_2360365, EPI_ISL_2360367, EPI_ISL_2360368, EPI_ISL_2360369, EPI_ISL_2360372, EPI_ISL_2360373, EPI_ISL_2360374, EPI_ISL_2360375, EPI_ISL_2360377, EPI_ISL_2360378, EPI_ISL_2360379, EPI_ISL_2360380, EPI_ISL_2360381, EPI_ISL_2360384, EPI_ISL_2360385, EPI_ISL_2360387, EPI_ISL_2360388, EPI_ISL_2360389, EPI_ISL_2360391, EPI_ISL_2360392, EPI_ISL_2360395, EPI_ISL_2360396, EPI_ISL_2360397, EPI_ISL_2360412, EPI_ISL_2360413, EPI_ISL_2360418, EPI_ISL_2360419, EPI_ISL_2360420, EPI_ISL_2360421, EPI_ISL_2360422, EPI_ISL_2360423, EPI_ISL_2360424, EPI_ISL_2360425, EPI_ISL_2360449, EPI_ISL_2360450, EPI_ISL_2360451, EPI_ISL_2360452, EPI_ISL_2360453, EPI_ISL_2360454, EPI_ISL_2360455, EPI_ISL_2360456, EPI_ISL_2360457, EPI_ISL_2360458, EPI_ISL_2360459, EPI_ISL_2360469, EPI_ISL_2360470, EPI_ISL_2360471, EPI_ISL_2360472, EPI_ISL_2360473, EPI_ISL_2360474, EPI_ISL_2360475, EPI_ISL_2360476, EPI_ISL_2360477, EPI_ISL_2360478, EPI_ISL_2360479, EPI_ISL_2360480, EPI_ISL_2360481, EPI_ISL_2360482, EPI_ISL_2360483, EPI_ISL_2360484, EPI_ISL_2360485, EPI_ISL_2360486, EPI_ISL_2360487, EPI_ISL_2360488, EPI_ISL_2360489, EPI_ISL_2360490, EPI_ISL_2360491, EPI_ISL_2360492, EPI_ISL_2360493, EPI_ISL_2360494, EPI_ISL_2360495, EPI_ISL_2360496, EPI_ISL_2360497, EPI_ISL_2360498, EPI_ISL_2360499, EPI_ISL_2360500, EPI_ISL_2360501, EPI_ISL_2360502, EPI_ISL_2360503, EPI_ISL_2360504, EPI_ISL_2360505, EPI_ISL_2360506, EPI_ISL_2360507, EPI_ISL_2360508, EPI_ISL_2360509, EPI_ISL_2360510, EPI_ISL_2360511, EPI_ISL_2360512, EPI_ISL_2360513, EPI_ISL_2360514, EPI_ISL_2360515, EPI_ISL_2360516, EPI_ISL_2360517, EPI_ISL_2360518, EPI_ISL_2360524, EPI_ISL_2360525, EPI_ISL_2360526, EPI_ISL_2360527, EPI_ISL_2360528, EPI_ISL_2360529, EPI_ISL_2360550, EPI_ISL_2360551, EPI_ISL_2360552, EPI_ISL_2360554, EPI_ISL_2360557, EPI_ISL_2360570, EPI_ISL_2360571, EPI_ISL_2360572, EPI_ISL_2360573, EPI_ISL_2360574, EPI_ISL_2360575, EPI_ISL_2360576, EPI_ISL_2360597, EPI_ISL_2360598, EPI_ISL_2360601, EPI_ISL_2360608, EPI_ISL_2360612, EPI_ISL_2360613, EPI_ISL_2360614, EPI_ISL_2360617, EPI_ISL_2360634, EPI_ISL_2360638, EPI_ISL_2360642, EPI_ISL_2360643, EPI_ISL_2360644, EPI_ISL_2360645, EPI_ISL_2360646, EPI_ISL_2360647, EPI_ISL_2360649, EPI_ISL_2360660, EPI_ISL_2360661, EPI_ISL_2360666, EPI_ISL_2360667, EPI_ISL_2360669, EPI_ISL_2360670, EPI_ISL_2360671, EPI_ISL_2360675, EPI_ISL_2360677, EPI_ISL_2360678, EPI_ISL_2360679, EPI_ISL_2360680, EPI_ISL_2360692, EPI_ISL_2360693, EPI_ISL_2360694, EPI_ISL_2360695, EPI_ISL_2360701, EPI_ISL_2360703, EPI_ISL_2360704, EPI_ISL_2360707, EPI_ISL_2360708, EPI_ISL_2360709, EPI_ISL_2360712, EPI_ISL_2360721, EPI_ISL_2360722, EPI_ISL_2360723, EPI_ISL_2360730, EPI_ISL_2360731, EPI_ISL_2360732, EPI_ISL_2360733, EPI_ISL_2360735, EPI_ISL_2360736, EPI_ISL_2360737, EPI_ISL_2360738, EPI_ISL_2360739, EPI_ISL_2360740, EPI_ISL_2360741, EPI_ISL_2360743, EPI_ISL_2360744, EPI_ISL_2360745, EPI_ISL_2360746, EPI_ISL_2360758, EPI_ISL_2360763, EPI_ISL_2360764, EPI_ISL_2360765, EPI_ISL_2360766, EPI_ISL_2360768, EPI_ISL_2360771, EPI_ISL_2360772, EPI_ISL_2360775, EPI_ISL_2360776, EPI_ISL_2360782, EPI_ISL_2360785, EPI_ISL_2360787, EPI_ISL_2360792, EPI_ISL_2360794, EPI_ISL_2360828, EPI_ISL_2360829, EPI_ISL_2360839, EPI_ISL_2360849, EPI_ISL_2360851, EPI_ISL_2360852, EPI_ISL_2360854, EPI_ISL_2360862, EPI_ISL_2360863, EPI_ISL_2360864, EPI_ISL_2360865, EPI_ISL_2360871, EPI_ISL_2360872, EPI_ISL_2360876, EPI_ISL_2360881, EPI_ISL_2360882, EPI_ISL_2360883, EPI_ISL_2360889, EPI_ISL_2360892, EPI_ISL_2360893, EPI_ISL_2360902, EPI_ISL_2360903, EPI_ISL_2360904, EPI_ISL_2360905, EPI_ISL_2360907, EPI_ISL_2360908, EPI_ISL_2360909, EPI_ISL_2360910, EPI_ISL_2360916, EPI_ISL_2360917, EPI_ISL_2360918, EPI_ISL_2360919, EPI_ISL_2360920, EPI_ISL_2360921, EPI_ISL_2360925, EPI_ISL_2360926, EPI_ISL_2360934, EPI_ISL_2360935, EPI_ISL_2360940, EPI_ISL_2360941, EPI_ISL_2360944, EPI_ISL_2360954, EPI_ISL_2360955, EPI_ISL_2360962, EPI_ISL_2360964, EPI_ISL_2360966, EPI_ISL_2361002, EPI_ISL_2361013, EPI_ISL_2361014, EPI_ISL_2361018, EPI_ISL_2361026, EPI_ISL_2361027, EPI_ISL_2361047, EPI_ISL_2361048, EPI_ISL_2361049, EPI_ISL_2361050, EPI_ISL_2361051, EPI_ISL_2361052, EPI_ISL_2361053, EPI_ISL_2361054, EPI_ISL_2361055, EPI_ISL_2361056, EPI_ISL_2361057, EPI_ISL_2361058, EPI_ISL_2361067, EPI_ISL_2361071, EPI_ISL_2361072, EPI_ISL_2361073, EPI_ISL_2361074, EPI_ISL_2361077, EPI_ISL_2361078, EPI_ISL_2361079, EPI_ISL_2361080, EPI_ISL_2361081, EPI_ISL_2361082, EPI_ISL_2361083, EPI_ISL_2361084, EPI_ISL_2361085, EPI_ISL_2361086, EPI_ISL_2361087, EPI_ISL_2361088, EPI_ISL_2494358, EPI_ISL_2494361, EPI_ISL_2494362, EPI_ISL_2494370, EPI_ISL_2494375, EPI_ISL_2494377, EPI_ISL_2494383, EPI_ISL_2494384, EPI_ISL_2494385, EPI_ISL_2494401, EPI_ISL_2494410, EPI_ISL_2494415, EPI_ISL_2494419, EPI_ISL_2494467, EPI_ISL_2494473, EPI_ISL_2494477, EPI_ISL_2494483, EPI_ISL_2494496, EPI_ISL_2494497, EPI_ISL_2494498, EPI_ISL_2494518, EPI_ISL_2494521, EPI_ISL_2494524, EPI_ISL_2494533, EPI_ISL_2494535, EPI_ISL_2494544, EPI_ISL_2494545, EPI_ISL_2494546, EPI_ISL_2494548, EPI_ISL_2494550, EPI_ISL_2494554, EPI_ISL_2494561, EPI_ISL_2494565, EPI_ISL_2494571, EPI_ISL_2494572, EPI_ISL_2494573, EPI_ISL_2494597, EPI_ISL_2494599, EPI_ISL_2494614, EPI_ISL_2494629			
see above	Vaccines and Infectious Diseases Analytics Research Unit (VIDA)	KRISP, KZn Research Innovation and Sequencing Platform	Baillie Vicky; Giandhari Jennifer; Madhi Shabir; Naidoo Yeshnee; Pillay Sureshnee; San James; Tegally Houriiyah; Tshabuila Derek; Wilkinson Eduan; de Oliveira Tulio; du Plessis Jeanine
EPI_ISL_6204413	Vermaak	National Institute for Communicable Diseases of the National Health Laboratory Service	Amoako DG; Bhiman JN; Everatt J; Ismail A; Mahlangu B; Mnguni A; Mohale T; Ntuli N; Scheepers C
